# CD49b identifies functionally and epigenetically distinct subsets of lineage-biased hematopoietic stem cells

**DOI:** 10.1016/j.stemcr.2022.05.014

**Published:** 2022-06-16

**Authors:** Ece Somuncular, Julia Hauenstein, Prajakta Khalkar, Anne-Sofie Johansson, Özge Dumral, Nicolai S. Frengen, Charlotte Gustafsson, Giuseppe Mocci, Tsu-Yi Su, Hugo Brouwer, Christine L. Trautmann, Michael Vanlandewijck, Stuart H. Orkin, Robert Månsson, Sidinh Luc

**Affiliations:** 1 Center for Hematology and Regenerative Medicine, Karolinska Institutet, Stockholm, Sweden; 2 Department of Medicine Huddinge, Karolinska Institutet, Stockholm, Sweden; 3 Department of Laboratory Medicine, Karolinska Institutet, Stockholm, Sweden; 4 Single Cell Core Facility of Flemingsberg Campus, Karolinska Institutet, Stockholm, Sweden; 5 Department of Immunology, Genetics and Pathology, Rudbeck Laboratory, Uppsala University, Uppsala, Sweden; 6 Dana-Farber/Boston Children’s Cancer and Blood Disorders Center, Harvard Medical School, Howard Hughes Medical Institute, Boston, MA, USA

**Keywords:** hematopoietic stem cells, HSC heterogeneity, lineage bias, single-cell transplantation, ATAC-seq, RNA-seq, epigenetic regulation, CD49b

## Abstract

Hematopoiesis is maintained by functionally diverse lineage-biased hematopoietic stem cells (HSCs). The functional significance of HSC heterogeneity and the regulatory mechanisms underlying lineage bias are not well understood. However, absolute purification of HSC subtypes with a pre-determined behavior remains challenging, highlighting the importance of continued efforts toward prospective isolation of homogeneous HSC subsets. In this study, we demonstrate that CD49b subdivides the most primitive HSC compartment into functionally distinct subtypes: CD49b^−^ HSCs are highly enriched for myeloid-biased and the most durable cells, while CD49b^+^ HSCs are enriched for multipotent cells with lymphoid bias and reduced self-renewal ability. We further demonstrate considerable transcriptional similarities between CD49b^−^ and CD49b^+^ HSCs but distinct differences in chromatin accessibility. Our studies highlight the diversity of HSC functional behaviors and provide insights into the molecular regulation of HSC heterogeneity through transcriptional and epigenetic mechanisms.

## Highlights

• CD49b^−^ HSCs are highly enriched for durable and long-term myeloid-biased HSCs
• CD49b^+^ HSCs are enriched for less durable cells with lymphoid bias
• CD49b^−^ and CD49b^+^ HSCs are transcriptionally similar but epigenetically distinct

## Teaser

Luc and colleagues have found that CD49b phenotypically separates primitive hematopoietic stem cells (HSCs) into functionally distinct lineage-biased subsets. RNA-seq analysis revealed highly overlapping gene expression profiles, whereas ATAC-seq analysis showed discrete chromatin accessibility patterns between the functionally different subsets. The findings establish a new approach to purify myeloid-biased long-term HSCs using CD49b by separating out epigenetically distinct lymphoid-biased HSCs.

## Introduction

The maintenance and replenishment of the hematopoietic system and its cells rely on rare bone marrow (BM)-resident hematopoietic stem cells (HSCs). Blood cell development is traditionally described as a hierarchical tree, with HSCs differentiating through progenitor stages to ultimately form the terminally differentiated hematopoietic cells (Wilkinson et al., 2020). In this model, HSCs are assumed to be multipotent and equipotent, thus producing all mature blood cells with no lineage preference (Eaves, 2015). However, HSCs exhibit distinct functional behaviors *in vivo*, with only a subset of them showing a lineage-balanced output consistent with traditionally described HSCs. The functionally heterogeneous HSCs have been classified according to the ratio of mature myeloid to lymphoid cells within the leukocyte fraction (Eaves, 2015). Later studies, which included the analysis of platelet and erythroid cells, led to the discovery of additional biased and restricted HSCs (Wilkinson et al., 2020). To date, HSCs categorized by myeloid-biased, platelet-biased, lineage-balanced, and lymphoid-biased repopulating patterns have been demonstrated (Carrelha et al., 2018; Challen et al., 2010; Dykstra et al., 2007; Morita et al., 2010; Müller-Sieburg et al., 2002; Muller-Sieburg et al., 2004; Sanjuan-Pla et al., 2013; Yamamoto et al., 2013, 2018). The behavior of distinct HSC subsets differs not only in lineage bias, but also in self-renewal ability (Eaves, 2015; Wilkinson et al., 2020). Myeloid-biased, platelet-biased, and lineage-balanced HSCs are recognized as durable HSCs that can sustain long-term (LT) hematopoiesis (Carrelha et al., 2018; Dykstra et al., 2007; Eaves, 2015; Morita et al., 2010; Sanjuan-Pla et al., 2013). Lymphoid-biased HSCs generally have finite self-renewal ability and likely overlap with intermediate-term (IT) HSCs and, thus, are not considered LT HSCs, but are still distinct from transiently self-renewing short-term (ST) HSCs and multipotent progenitors (MPPs) (Benveniste et al., 2010; Challen et al., 2010; Dykstra et al., 2007; Eaves, 2015; Kent et al., 2009; Morita et al., 2010). Altogether, these studies suggest that there is a large diversity of cells within the traditional HSC compartment, which exhibit lineage-bias differences and gradual differences in self-renewal potential and thereby durability.

Stem and progenitor cells are contained within the Lineage^−^Sca-1^+^c-Kit^+^ (LSK) compartment, but only a minor part of LSK cells are LT reconstituting HSCs. The combination of additional cell-surface markers has greatly improved the isolation of functional HSCs. However, HSCs are ultimately defined by their functional ability and cannot be identified by immunophenotype alone (Wilkinson et al., 2020). Lineage bias is, at least partly, thought to be intrinsically programmed. Further study of the mechanisms underlying HSC diversity is dependent upon the ability to connect HSC immunophenotype with functional behavior, highlighting the importance of prospective isolation of homogeneous HSC subsets (Eaves, 2015; Haas et al., 2018).

In this study, we have reassessed the phenotypic HSC compartment using cell-surface markers reported to identify HSCs, to explore whether different combinations of immunophenotypes can isolate functionally diverse HSC subsets. We found heterogeneous expression of CD49b in the phenotypic HSC fraction harboring the most primitive and durable cells (LSKCD34^−^CD48^−^CD150^hi^) (Morita et al., 2010). Phenotypically separated CD49b fractions were functionally distinct: CD49b^−^ cells were highly enriched for myeloid-biased HSCs and were the most durable cells, and CD49b^+^ cells were enriched for multipotent HSCs with lymphoid bias and less durability. Transcriptional profiling of CD49b^−^ and CD49b^+^ HSCs revealed high concordance, whereas chromatin accessibility analysis showed diverse profiles. Our studies demonstrate that CD49b can distinguish between functionally and epigenetically distinct multipotent HSCs with myeloid and lymphoid bias in the primitive HSC compartment.

## Results

### The HSC compartment can be further subfractionated with CD49b

**Figure 1:**
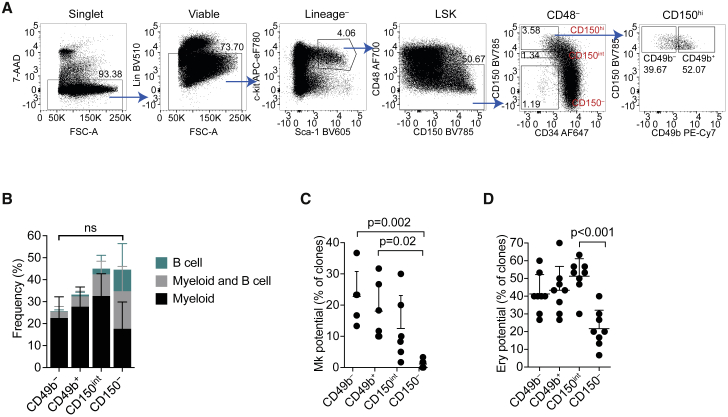
The HSC compartment can be further subfractionated with CD49b (A) Fluorescence-activated cell sorting (FACS) profile and gating strategy of phenotypic HSC subsets and further separation with CD49b in CD117-enriched BM cells. Frequencies of parent gates are shown. (B) *In vitro* differentiation potential of single sorted cells to myeloid (CD11b^+^Gr-1^+^ and/or CD11b^+^F4/80^+^) and B cells (B220^+^CD19^+^, n_*CD49b*_^−^ = 568 cells, n_*CD49b*_^+^ = 536 cells, n_*CD150*_^*int*^ = 401 cells, n_*CD150*_^−^ = 409 cells; nine replicates; five independent experiments). ns, not significant. (C) Megakaryocyte differentiation culture of single plated cells (n = 360 cells/population, six replicates, three independent experiments). (D) Erythroid colony-forming assay of CD49b^−^, CD49b^+^, CD150^int^, and CD150^−^ cells (n = 8 replicates/population, 30 cells per replicate, two independent experiments). Mean ± SD is shown in (B–D). Statistical significance in (B) was calculated based on total cloning frequency. See also Figure S1.

We tested previously reported HSC markers to explore further subfractionation, including CD41 and CD244 (Kiel et al., 2005), Flt-3 (Adolfsson et al., 2001), Tie-2 (Arai et al., 2004), CD201 (EPCR; Balazs et al., 2006), CD61 (Mann et al., 2018), CD86 (Shimazu et al., 2012), CD9 (Karlsson et al., 2013), Esam (Sudo et al., 2012), CD229 (Oguro et al., 2013), and CD49b (Wagers and Weissman, 2006) (Figure S1A). Since CD150 expression is positively correlated with self-renewal, we focused primarily on the LSKCD34^−^CD48^−^CD150^hi^ (CD150^hi^) fraction, enriched for myeloid-biased HSCs (Figure 1A). The LSKCD34^−^CD48^−^CD150^int^ (CD150^int^) cells, enriched for lineage-balanced HSCs, and the LSKCD34^−^CD48^−^CD150^−^ (CD150^−^) cells, with a lymphoid-biased phenotype, were included for comparison (Figure 1A) (Kent et al., 2009; Morita et al., 2010). Most markers had uniform expression patterns in the CD150^hi^ fraction except for CD229, CD41, and CD49b, which showed bimodal expression profiles (Figure S1A). Interestingly, CD49b has been suggested to mark ST HSCs, IT HSCs, and primed HSCs (Benveniste et al., 2010; Wagers and Weissman, 2006; Zhao et al., 2019). We found that previously identified CD49b^−^ and CD49b^+^ populations were heterogeneous for CD150 cell-surface expression, which could partly explain why CD49b marks cells with both transient and LT self-renewal ability (Figures S1B and S1C). We therefore hypothesized that CD49b might be a candidate marker to further enhance the isolation of functionally distinct HSCs within the CD150^hi^ compartment. Notably, the combination of CD229, CD41, and CD49b revealed further phenotypic subfractions within CD150^hi^ cells (Figure S1D). However, cell-cycle analysis by Ki-67 staining and cell-proliferation analysis by the 5-bromo-2′-deoxyuridine (BrdU) incorporation assay showed significant differences only between the CD49b^−^ and the CD49b^+^ subsets (Figures S1E–S1H). These data suggested that subfractionation with CD49b alone could be sufficient to isolate functionally distinct cells.

We therefore isolated LSKCD34^−^CD48^−^CD150^hi^CD49b^−^ (CD49b^−^) and LSKCD34^−^CD48^−^CD150^hi^CD49b^+^ (CD49b^+^) cells (Figures 1A and S1I) to assess *in vitro* differentiation abilities (Figures 1B–1D). In combined B cell and myeloid cell (B/M) cultures, all investigated subsets produced B cells and myeloid cells. However, CD49b^−^ and CD49b^+^ subsets mainly generated myeloid cells, consistent with the CD150^hi^ fraction containing myeloid-biased cells. In contrast, CD150^−^ cells efficiently produced B cells, in line with enrichment of lymphoid-biased cells in the CD150^−^ fraction (Figure 1B) (Kent et al., 2009; Morita et al., 2010). Furthermore, CD49b^−^ and CD49b^+^ subsets efficiently differentiated into megakaryocytes and erythroid cells, whereas the CD150^−^ fraction was the least efficient (Figures 1C and 1D). Collectively, these results suggested that CD49b^−^ and CD49b^+^ cells have similar *in vitro* differentiation abilities.

### CD49b^−^ and CD49b^+^ subsets have different cell-cycle and cell-proliferation kinetics

**Figure 2:**
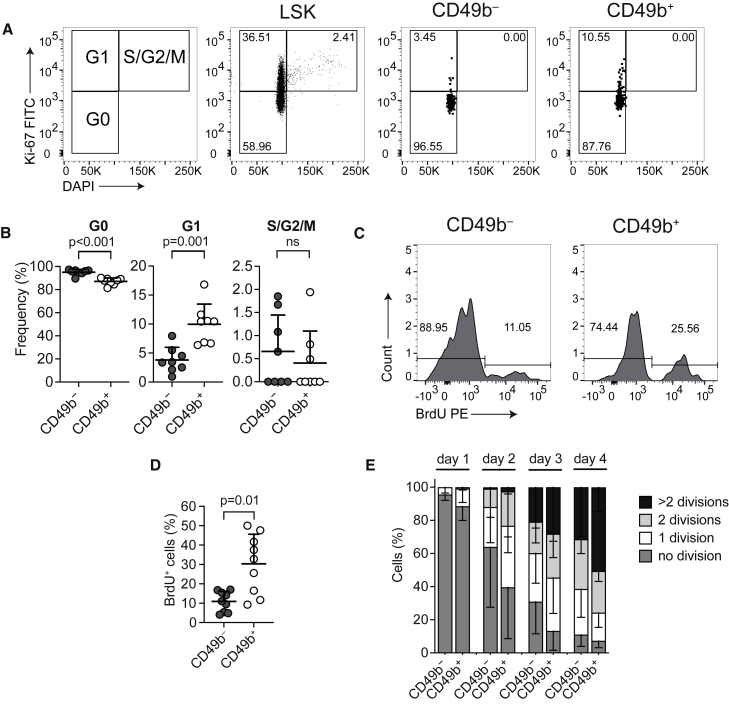
CD49b^−^ and CD49b^+^ subsets have different cell-cycle and cell-proliferation kinetics (A) Cell-cycle analysis of CD117-enriched BM cells. The frequency of G0, G1, and S/G2/M cells in a representative mouse is shown. (B) Frequency of CD49b^−^ and CD49b^+^ HSCs in G0, G1, and S/G2/M (n = 8 mice, two independent experiments). ns, not significant. (C) BrdU analysis of CD117-enriched BM cells. The frequency of BrdU^−^ and BrdU^+^ cells in a representative mouse is shown. (D) Frequency of BrdU^+^ CD49b^−^ and CD49b^+^ HSCs (n = 9 mice, three independent experiments). (E) Cell divisions from cultured single cells on days 1–4 (n_*CD49b*_^−^ = 347 cells, n_*CD49b*_^+^ = 370 cells, five replicates, three independent experiments). Mean ± SD is shown in (B), (D), and (E).

Despite the lack of functional differences between CD49b^−^ and CD49b^+^ cells *in vitro*, we observed a higher, but not statistically significant, cloning frequency from the CD49b^+^ subset in the B/M assay, compatible with cell-proliferation differences (Figures 1B and S1H). Cell-cycle analysis of CD49b^−^ and CD49b^+^ cells showed that both subsets were highly quiescent, but CD49b^−^ cells were more in G0 (95%) and less in G1 (4%) compared with CD49b^+^ cells (87% and 10%, respectively; Figures 2A and 2B). Consistent with the higher *in vitro* cloning frequency (Figure 1B), more CD49b^+^ cells (30%) incorporated BrdU compared with CD49b^−^ cells (11%) (Figures 2C and 2D). To further understand cell-proliferation kinetics, we tracked the cell divisions of CD49b^−^ and CD49b^+^ single cells (Figure 2E). One day after culture, most cells had still not divided (CD49b^−^, 96%; CD49b^+^, 89%), consistent with the quiescent nature of both subsets (Figure 2B). However, after 2 days, 60% of CD49b^+^ cells had already undergone ≥1 cell division, compared with only 36% of CD49b^−^ cells, indicating that CD49b^+^ cells entered the cell cycle faster. By 4 days, the numbers of cell divisions were comparable between the subsets.

Collectively, these findings suggested that, although both CD49b^−^ and CD49b^+^ subsets were highly quiescent, CD49b^+^ cells had a higher proliferation rate.

### CD49b^−^ and CD49b^+^ HSCs are the most durable subsets

**Figure 3:**
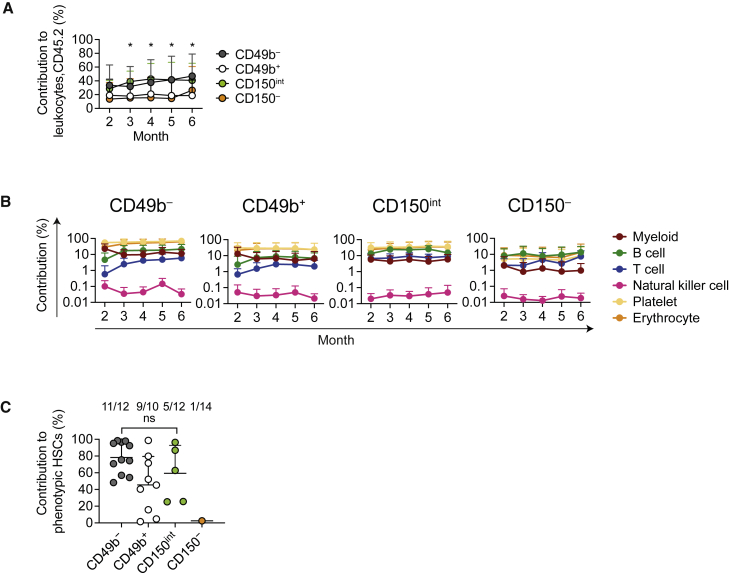
CD49b^−^ and CD49b^+^ HSCs are the most durable subsets (A and B) Total donor contribution (A) and donor contribution to platelets, erythrocytes, and myeloid, B, T, and natural killer cells (B) in peripheral blood (PB) from five cell transplantations (n_*CD49b*_^−^ = 28 mice, n_*CD49b*_^+^ = 22 mice, n_*CD150*_^*int*^ = 12 mice, n_*CD150*_^−^ = 13 mice, three independent experiments). (C) Donor contribution to phenotypic HSCs (LSKFlt-3^−^CD48^−^CD150^+^ or LSKCD48^−^CD150^+^), 5–6 months after five cell transplantations (n_*CD49b*_^−^ = 12 mice, n_*CD49b*_^+^ = 10 mice, n_*CD150*_^*int*^ = 12 mice, n_*CD150*_^−^ = 13 mice, three independent experiments). The numbers of reconstituted mice out of all mice analyzed are indicated. ns, not significant. Asterisks indicate statistically significant differences: p = 0.028, CD49b^−^ versus CD150^−^, and p = 0.05, CD150^int^ versus CD150^−^, month 2; p = 0.032, CD49b^−^ versus CD150^−^, and p = 0.006, CD150^int^ versus CD150^−^, month 3; p = 0.015, CD49b^−^ versus CD150^−^, and p = 0.005, CD150^int^ versus CD150^−^, month 4; p = 0.005, CD49b^−^ versus CD150^−^, and p = 0.013, CD150^int^ versus CD150^−^, month 5; and p = 0.016, CD49b^−^ versus CD49b^+^, month 6. Mean ± SD is shown in (A−C). See also Figure S2.

To evaluate the functional significance of the CD49b subfractions, we performed a competitive transplantation assay, in which five cells of the CD49b^−^, CD49b^+^, CD150^int^, or CD150^−^ subsets were transplanted. Donor HSC subsets were purified from the *Gata-1* eGFP mouse strain to detect platelets and erythrocytes (Figure S2A) (Drissen et al., 2016). Although the transplanted HSC subsets differed in total leukocyte contribution, they repopulated all blood cells (Figures 3A, 3B, and S2B). While the CD49b^−^, CD49b^+^, and CD150^int^ subsets could stably generate all blood lineages for 6 months, CD150^−^ transplanted mice exhibited transient myeloid repopulation but sustained high B and T cell repopulation over time, consistent with previous studies (Kent et al., 2009; Morita et al., 2010). Furthermore, donor-derived phenotypic HSCs were detected in most mice from the CD49b^−^ (92%) and CD49b^+^ (90%) groups, in 42% of mice from CD150^int^, but in only 7% of mice from CD150^−^ (Figures 3C and S2C). Mice from the CD49b^−^ and CD49b^+^ groups generated all types of mature BM cells, progenitor cells, and phenotypic HSCs at 5–6 months post-transplantation (Figures 3C and S2D–S2F). Collectively, these findings indicated that CD49b^−^ and CD49b^+^ subsets had the highest self-renewal potential.

### CD49b^−^ and CD49b^+^ HSCs reconstitute all blood lineages, but at different ratios

**Figure 4:**
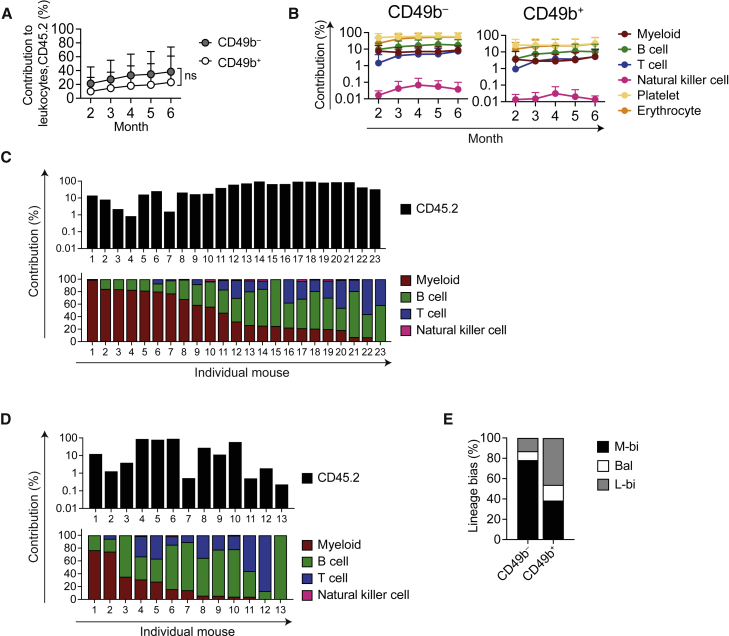
CD49b^−^ and CD49b^+^ HSCs reconstitute all blood lineages, but at different ratios (A and B) Total donor contribution (A) and donor contribution to platelets, erythrocytes, and myeloid, B, T, and natural killer cells (B) in PB from single-cell transplantation (n_*CD49b*_^−^ = 28 mice, n_*CD49b*_^+^ = 18 mice, five independent experiments). ns, not significant. (C and D) Total donor contribution (top) and relative contribution to myeloid, B, T, and natural killer cells (bottom) in PB of CD49b^−^ (C) or CD49b^+^ (D) single-cell-transplanted mice, 5–6 months post-transplantation (n_*CD49b*_^−^ = 23 mice, n_*CD49b*_^+^ = 13 mice, five independent experiments). (E) Lineage bias categorization of single-cell-transplanted mice 5–6 months post-transplantation (n_*CD49b*_^−^ = 23 mice, n_*CD49b*_^+^ = 13 mice, five independent experiments). M-bi, myeloid-biased; Bal, balanced; L-bi, lymphoid-biased. Mean ± SD is shown (A and B). See also Figures S3–S5.

To evaluate the HSC behavior at the clonal level, we performed single-cell transplantation experiments. A total of 139 mice were transplanted with single CD49b^−^ (61 mice) or CD49b^+^ (78 mice) cells, and 48% of the CD49b^−^ and 28% of the CD49b^+^ cells reconstituted the recipients (Figure S3). Both subsets were able to repopulate all leukocyte, platelet, and erythrocyte lineages for 6 months (Figures 4A and 4B). The repopulation profiles of individual mice revealed five major patterns from CD49b^−^ cells where the dominant groups were characterized by high platelet (P), erythroid (E), and myeloid contributions (M > L) and multilineage contributions (M-L) (Figures S3 and S4A). Notably, mice with only P or PE repopulation patterns were found only in CD49b^−^ cells (Figures S3E, S3F, and S4A). Conversely, CD49b^+^ cells could be grouped into four patterns with predominantly higher B lymphoid reconstitution (L > M) or ST/transient repopulation patterns (Figure S4B). Thus, we assessed the lineage bias by analyzing the proportional contribution to B, T, natural killer (NK), and myeloid cells in donor leukocytes (Figures 4C and 4D). Single-cell-transplanted CD49b^−^ mice showed a high myeloid contribution, and CD49b^+^ mice exhibited predominantly lymphoid contribution and some lymphoid-restricted patterns. Based on blood profiles from unmanipulated mice, a lineage-balanced pattern can be categorized by the ratio of lymphoid to myeloid cell contribution (L/M) of 6.0 ± 2.0 (Figure S4C) (Müller-Sieburg et al., 2002). Thus, we categorized the repopulation patterns from the CD49b^−^ and CD49b^+^ single-cell-transplanted mice as myeloid-biased (L/M < 4), lymphoid-biased (L/M > 8), or lineage-balanced (L/M ≥ 4 and ≤ 8). CD49b^−^-transplanted mice were predominantly classified as myeloid-biased (M-bi; 78%), whereas the most common classification in CD49b^+^-transplanted mice was lymphoid-biased (L-bi; 46%, Figure 4E). Furthermore, CD49b^−^ generated more myeloid cells in the BM compared with CD49b^+^, consistent with the M-bi repopulation pattern in peripheral blood (PB), while the lymphoid cell contributions in CD49b^−^ and CD49b^+^ cells were comparable (Figure S4D). These findings suggested that both CD49b^−^ and CD49b^+^ subsets were multipotent but with different lineage biases.

### CD49b^−^ HSCs are the most durable stem cells

**Figure 5:**
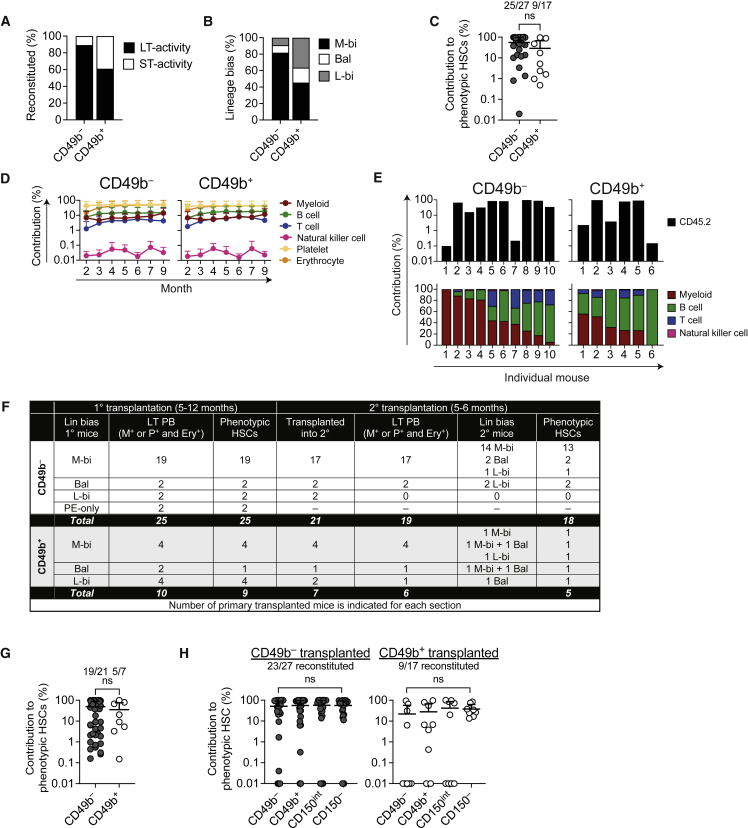
CD49b^−^ HSCs are the most durable stem cells (A) Short-term (ST) or long-term (LT) HSC activity of reconstituted single-cell-transplanted mice (n_*CD49b*_^−^ = 28 mice, n_*CD49b*_^+^ = 18 mice, five independent experiments). LT defined as myeloid (M)^+^ or platelet (P)^+^ and erythrocyte (Ery)^+^ in PB, 5–6 months post-transplantation. (B) Lineage bias distribution of single-cell-transplanted mice with LT activity in (A) (n_*CD49b*_^−^ = 25 mice, n_*CD49b*_^+^ = 11 mice, five independent experiments). (C) Donor contribution to phenotypic HSCs (LSKFlt-3^−^CD48^−^CD150^+^ or LSKCD48^−^CD150^+^) 5–12 months after single-cell transplantation (n_*CD49b*_^−^ = 27 mice, n_*CD49b*_^+^ = 17 mice, five independent experiments). ns, not significant. (D) Donor contribution to platelets, erythrocytes, and myeloid, B, T, and natural killer cells in PB from single-cell transplantation (n_*CD49b*_^−^ = 10 mice, n_*CD49b*_^+^ = 6 mice, two independent experiments). (E) Total donor contribution (top) and relative contribution to myeloid, B, T, and natural killer cells (bottom) in PB of CD49b^−^ and CD49b^+^ single-cell-transplanted mice, 9 months post-transplantation (n_*CD49b*_^−^ = 10 mice, n_*CD49b*_^+^ = 6 mice, two independent experiments). (F) Overview of CD49b^−^ and CD49b^+^ primary reconstituted single-cell-transplanted mice with LT activity from (A). The categorized lineage bias and number of mice with donor contribution to PB and phenotypic HSCs (LSKFlt-3^−^CD48^−^CD150^+^ or LSKCD48^−^CD150^+^) 5–12 months post-transplantation, in primary and secondary transplantation, are indicated. (G) Donor contribution to phenotypic HSCs (LSKFlt-3^−^CD48^−^CD150^+^ or LSKCD48^−^CD150^+^) in secondary transplantation with whole BM cells, 5–6 months post-transplantation (n_*CD49b*_^−^ = 50, n_*CD49b*_^+^ = 8 mice, five independent experiments). ns, not significant. (H) Donor contribution to phenotypic lineage-biased HSC subsets, 5–12 months post-transplantation, from single-cell-transplanted mice (n_*CD49b*_^−^ = 27 mice, n_*CD49b*_^+^ = 17 mice, five independent experiments). The number of reconstituted primary donor mice out of all mice analyzed is indicated in (C), (G) and (H). Mean ± SD is shown (C, D, G, H). ns, not significant. See also Figures S4 and S5.

An L-bi repopulation pattern is generally associated with declining myeloid repopulation and limited self-renewal ability due to a lack of LT HSC activity (Kent et al., 2009; Morita et al., 2010). We therefore assessed the LT (≥5–6 months) and ST (<5–6 months) repopulating ability of CD49b^−^ and CD49b^+^ subsets based on the presence of myeloid cells or platelets and erythrocytes in PB. Eighty-nine percent of reconstituted mice transplanted with single CD49b^−^ cells exhibited LT repopulating ability, of which 82% were M-bi. In contrast, 61% of CD49b^+^-reconstituted mice showed LT activity, of which 36% were L-bi (Figures 5A and 5B). As expected, no CD49b^+^ cells with lymphoid-restricted patterns and LT activity were found (Figures 4D and 5A). Furthermore, both CD49b^−^ and CD49b^+^ subsets generated phenotypic HSCs and downstream BM progenitor populations, but less efficiently in CD49b^+^ compared with CD49b^−^ cells (Figures 5C, S4E, and S4F). These data showed that CD49b^−^ cells were more durable than CD49b^+^ cells.

To assess whether LT repopulating activity decreased over time, we followed a group of single-cell-transplanted mice to 9 months. Both the repopulation level and the pattern were similar to the results at 5–6 months post-transplantation (Figures 4B–4D, 5D, and 5E), suggesting no decline of self-renewal potential in CD49b^−^ and CD49b^+^ subsets over time.

To conclusively compare the self-renewal ability of CD49b^−^ and CD49b^+^ cells, mice with phenotypic HSC repopulation were secondarily transplanted. Overall, the repopulation pattern in PB was preserved from primary to secondary recipients (Figures 5F and S5A). The repopulation efficiency of CD49b^−^-transplanted mice was high, with all reconstituted mice with LT activity (Figure 5A) showing LT repopulation in PB and BM (25/25). Most of these mice further repopulated secondary recipients in PB and BM (18/21). In contrast, the repopulation efficiency of CD49b^+^ cells in both primary (9/10) and secondary (5/7) transplantation was lower, but they were still capable of multilineage repopulation (Figures 5F, 5G, S5A, and S5B).

An M-bi classification was highly correlated with positive and robust repopulation. In contrast, an L-bi classification was correlated with declining repopulation efficiency from primary to secondary transplantation. Although these findings indicated that L-bi cells were capable of propagating through serial transplantation with multilineage repopulation (Figures 5F, 5G, S5A, and S5B), our results nonetheless suggested that such cells were infrequent.

Given the difference in durability and lineage bias, we evaluated the hierarchical relationship between the CD49b subsets by assessing their ability to form phenotypic HSC subsets (CD49b^−^, CD49b^+^, CD150^int^, and CD150^−^). While CD49b^−^ cells were potent in generating all phenotypically defined HSC subsets, CD49b^+^ cells were less efficient, but nevertheless able to produce the same subsets (Figures 5H and S5C). Secondary transplantation confirmed that most of the phenotypic donor-derived CD49b^−^ and CD49b^+^ cells were functional HSCs (Figures S5D and S5E).

Collectively, these results showed that both CD49b^−^ and CD49b^+^ cells could sustain LT multilineage hematopoiesis with preserved lineage bias but at different efficiencies.

### The CD49b^−^ and CD49b^+^ subsets are transcriptionally similar but epigenetically distinct

**Figure 6:**
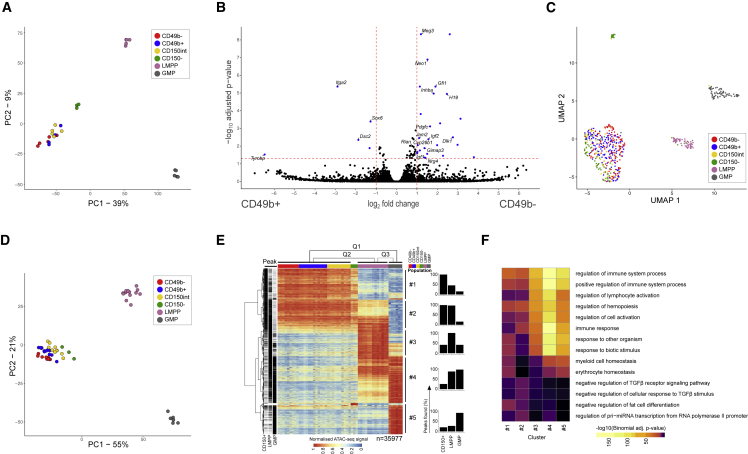
The CD49b^−^ and CD49b^+^ subsets are transcriptionally similar but epigenetically distinct (A) PCA of bulk RNA-seq data (n_*CD49b*_^−^ = 5, n_*CD49b*_^+^ = 4, n_*CD150*_^*int*^ = 5, n_*CD150*_^−^ = 3, n_LMPP_ = 5, n_GMP_ = 5 replicates, five independent experiments). (B) Volcano plot of differential expression between CD49b^−^ and CD49b^+^ subsets. Differentially expressed genes (p_adj_ < 0.05, |fold change| > 2) are marked in blue, with selected genes annotated. (C) UMAP visualization of single-cell RNA-seq data colored by cell population (n_*CD49b*_^−^ = 135, n_*CD49b*_^+^ = 146, n_*CD150*_^*int*^ = 59, n_*CD150*_^−^ = 77, n_LMPP_ = 57, n_GMP_ = 74 cells, two independent experiments). (D) PCA of ATAC-seq data (n_*CD49b*_^−^ = 9, n_*CD49b*_^+^ = 12, n_*CD150*_^*int*^ = 10, n_*CD150*_^−^ = 3, n_LMPP_ = 13, n_GMP_ = 6 replicates, seven independent experiments). (E) Heatmap (left) of row-normalized chromatin accessibility for differential regions (p_adj_ < 1 × 10^−4^, |fold change| > 2) in pairwise comparisons between CD150^+^, LMPP, and GMP populations. Regions are divided into five clusters based on hierarchical clustering. Bar graphs (right) show how many peaks were called in each cluster. (F) GO enrichment analysis of clusters 1–5. The top three significantly enriched terms for each cluster from the GO biological process are shown. See also Figures S6 and S7.

To assess the molecular basis of the diverse functional properties of CD49b subsets, we performed RNA sequencing (RNA-seq) on stem- and progenitor cell subsets and downstream lymphoid-primed MPPs (LMPPs) and granulocyte-monocyte progenitors (GMPs) (Table S1; Figures S6A and S6B). The investigated cells expressed the expected population-associated genes. Principal components analysis (PCA) showed that CD150^+^ (CD49b^−^, CD49b^+^, and CD150^int^), CD150^−^, LMPP, and GMP populations were well separated (Figures 6A and S6C), but CD150^+^ subsets did not cluster distinctly, suggesting high transcriptional overlap. Indeed, few significant differences were found between the CD49b^−^ and the CD49b^+^ subsets (Figure 6B). However, genes involved in preserving HSC dormancy and stemness properties, such as *Gfi1* (Hock et al., 2004) and *Dlk1-Meg3* (Qian et al., 2016), were upregulated in CD49b^−^ cells, consistent with their superior self-renewal potential. Only five genes, including *Itga2* (*CD49b*), were significantly upregulated in CD49b^+^ cells (Figure 6B). Consistent with bulk RNA-seq data, uniform manifold approximation and projection (UMAP) analysis of single-cell RNA-seq (scRNA-seq) could not resolve the subsets, and only eight genes, including *Gfi1*, were differentially expressed between CD49b^−^ and CD49b^+^ cells (Figures 6C, S6D, and S7A).

To identify epigenetic changes associated with gene expression differences, we analyzed open chromatin using assay for transposase-accessible chromatin sequencing (ATAC-seq, Table S2, Figures S6E and S6F) (Corces et al., 2017). Chromatin accessibility at population-specific genes followed the expected patterns (Figures S6G and S6H). PCA revealed distinct LMPP and GMP clusters, whereas CD150^+^ and CD150^−^ populations clustered closely together, but, in contrast to RNA-seq data, formed distinguishable groups (Figure 6D), indicating that transcriptionally similar CD49b^−^ and CD49b^+^ subsets differ on the chromatin level.

To assess chromatin accessibility changes from HSCs to progenitors, we performed pairwise comparisons between the CD150^+^, the LMPP, and the GMP populations, which created five clusters with significant differences (Figure 6E). Most regions with high accessibility in CD150^+^ populations (clusters 1–2) became inaccessible at progenitor stages. Conversely, regions accessible in LMPPs (cluster 3) and GMPs (clusters 4–5) were mainly inaccessible in the CD150^+^ group. Gene ontology (GO) analysis showed that high-accessibility regions in progenitors (clusters 3–5) were associated with myeloid and immune cell regulation, whereas regions in the CD150^+^ group (clusters 1–2) were associated with transforming growth factor β signaling, involved in regulation of HSC quiescence (Figure 6F) (Wang et al., 2018). These findings suggested that generation of downstream progenitors from CD150^+^ HSCs is associated with extensive chromatin remodeling.

### The CD49b^−^ and CD49b^+^ subsets exhibit distinct epigenetic changes

**Figure 7:**
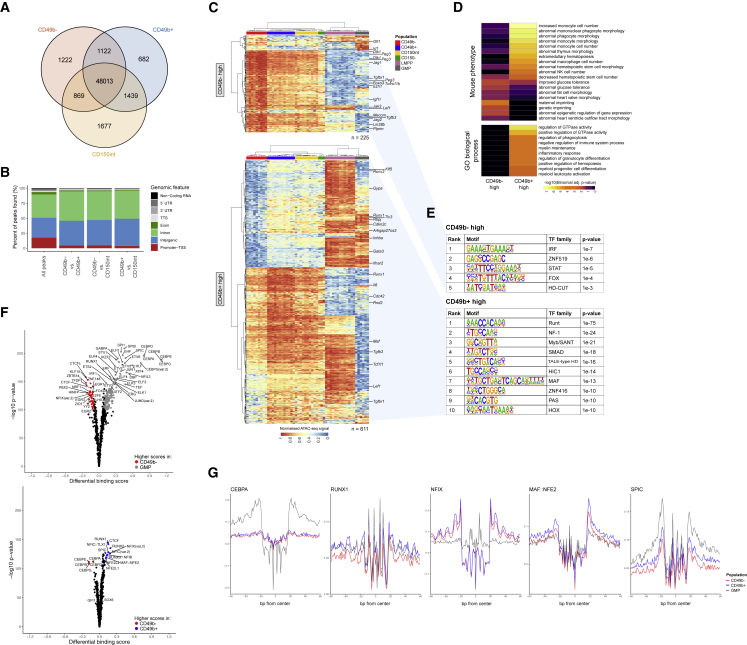
The CD49b^−^ and CD49b^+^ subsets exhibit distinct epigenetic changes (A) Overlap of open chromatin regions between CD150^+^ populations. Peaks with a normalized read count of >1.5 in more than one-third of samples were considered found. (B) Genomic feature distribution of regions with differential accessibility (p_adj_ < 0.01) and all regions as a reference ("all peaks"). (C) Heatmap of row-normalized chromatin accessibility for regions with differential accessibility (p_adj_ < 0.01) between CD49b^−^ (top; n = 225 peaks) and CD49b^+^ (bottom; n = 611 peaks) subsets. For selected regions the nearest gene is indicated. (D) GO enrichment analysis of regions with differential accessibility between CD49b^−^ and CD49b^+^ subsets. The top 10 significantly enriched terms from the mouse phenotype and GO biological process are shown. (E) Transcription factor (TF) families with enriched binding motifs (q <0.01, top 10) in regions with increased accessibility in CD49b^−^ (top) or CD49b^+^ (bottom) cells. (F) Volcano plots of differential TF binding. Transcription factors with differential binding activity (differential binding score >0.1, p < 1 × 10^−100^) are colored and selectively annotated. (G) Aggregated footprint plots for TFs with differential binding. See also Figure S7.

Since the CD150^+^ subsets formed distinct but closely related clusters (Figure 6D), we assessed the overlap of open chromatin. Most accessible regions were shared between the populations (87%; Figure 7A). Differentially accessible regions were preferentially localized in non-promoter regions (Figure 7B). There was a general increase in open chromatin regions in CD49b^+^ compared with CD49b^−^ cells (Figure 7C). Most regions with higher accessibility in CD49b^−^ cells had reduced accessibility in CD49b^+^ and downstream populations. Furthermore, the overall chromatin accessibilities in CD49b^+^, CD150^int^, and CD150^−^ populations were similar, indicating that the CD49b^−^ subset is developmentally upstream of the other populations. Consistent with RNA-seq data (Figure 6B), regions with increased accessibility in the CD49b^−^ cells were found proximal to *Gfi1* (Hock et al., 2004) and *Dlk1* (Qian et al., 2016) (Figure S7B). Conversely, in CD49b^+^ cells we found regions with higher accessibility adjacent to the hematopoietic regulators *Runx1*, *Runx3*, and *Maf* (Mevel et al., 2019; Sarrazin et al., 2009), but their expression was not significantly changed (Figures 7C and S7B). Gene ontology analysis (Figure 7D) showed that regions with increased accessibility in CD49b^−^ were associated with pathways involved in genetic imprinting, which regulates HSC quiescence (Qian et al., 2016). In contrast, processes regulating hematopoietic cell numbers, differentiation, activation, and GTPase activity (Mulloy et al., 2010) were associated with regions with higher accessibility in CD49b^+^. These findings are consistent with the cell-cycle active and proliferative nature of CD49b^+^ cells compared with the more quiescent CD49b^−^ cells.

To identify potential transcription factors (TFs) responsible for chromatin accessibility differences between the CD49b subsets, we performed motif enrichment analysis (Figure 7E). TF binding sites (TFBSs) associated with the FOX (Tothova and Gilliland, 2007) and STAT (Dorritie et al., 2014) families were among the most enriched motifs in CD49b^−^. Conversely, in CD49b^+^ cells, HOX (Alharbi et al., 2013) and RUNX (Mevel et al., 2019) family TFBSs were enriched (Figure 7E). Moreover, we performed a genome-wide analysis of TF occupancy by footprinting analysis (Figures 7F and 7G). Differential TF binding plots showed large differences in TF binding between CD49b^−^ and GMPs, which included the myeloid-associated C/EBP-family and SPI1 (PU.1) TFs (Tenen et al., 1997). In contrast, CD49b^−^ and CD49b^+^ had few TF binding differences. Consistent with the motif enrichment analysis, RUNX (RUNX1–3) (Mevel et al., 2019), NF-1 (including NFIB, NFIC, NFIX) (Harris et al., 2015), SPIC (Li et al., 2015), and MAF:NFE2 (Gasiorek and Blank, 2015) sites had significantly higher binding scores in CD49b^+^ cells (Figures 7F and 7G). Altogether, our findings indicated that differential functions of CD49b^−^ and CD49b^+^ cells may to a large extent be regulated by the same set of TFs.

## Discussion

It is well recognized that the HSC population is functionally diverse, with subtypes differing in propensity of blood cell differentiation and in self-renewal ability and lifespan (Eaves, 2015; Wilkinson et al., 2020). Although HSC heterogeneity is recognized, insight into the molecular mechanisms underlying HSC diversity is lacking due to limitations in purifying homogeneous HSC subtypes. Here, we have reassessed the expression of cell-surface markers suggested to define HSCs to explore functional heterogeneity. We identified CD49b as a candidate marker to subfractionate the phenotypic LSKCD34^−^CD48^−^CD150^hi^ compartment, enriched for M-bi and functional HSCs (Morita et al., 2010; Wilkinson et al., 2020). The CD49b^−^ subset greatly improved the purity of M-bi cells and cells with the highest self-renewal activity. However, the L-bi phenotype was most common in CD49b^+^ cells, with a subset exhibiting multilineage LT HSC activity. Notably, the CD49b^+^ L-bi HSCs in this study are distinct from the previously described CD150^−^ L-bi HSCs and γ and δ cells, which all showed a lymphoid-dominant repopulation pattern and limited self-renewal ability consistent with loss of LT HSC activity (Dykstra et al., 2007; Kent et al., 2009; Morita et al., 2010). Within the CD49b^+^ fraction such lymphoid dominant cells were categorized as ST/transient cells. Furthermore, CD49b^+^ cells described here were potent in generating platelets, erythrocytes, and lymphoid cells, but had low myeloid contribution, resulting in a lymphoid bias in the leukocyte compartment. Of note, the decline in platelet and erythrocyte reconstitution was associated with ST activity in lymphoid-dominant cells. There are several studies with findings compatible with the existence of multipotent LT L-bi HSCs (Challen et al., 2010; Dykstra et al., 2011; Oguro et al., 2013; Yamamoto et al., 2013). Our results, however, suggested that LT L-bi HSCs are infrequent in young adult mice, and although CD49b^+^ cells could sustain LT repopulation, they are less durable than CD49b^−^ cells.

CD49b has previously been used to identify and characterize ST (Wagers and Weissman, 2006) and IT HSCs (Benveniste et al., 2010) with finite self-renewal ability, indicating that CD49b expression is associated with reduced durability. Paradoxically, it has also been used to distinguish between reserve (CD49b^−^) and primed (CD49b^+^) HSCs (Zhao et al., 2019). While the previous studies showed opposing results in self-renewal ability of CD49b^+^ cells, lineage bias was not investigated. We show that the contradictory findings could partly be explained by the lack of CD150 in the immunophenotyping strategies, which greatly enriches for LT HSCs (Kiel et al., 2005). Although there is a degree of overlap, the CD49b^+^ cells in this study, which were identified from the primitive LSKCD34^−^CD48^−^CD150^hi^ compartment, exhibit distinct differences compared with the CD49b^+^ cells in previous studies (Benveniste et al., 2010; Zhao et al., 2019), particularly in lineage bias. Our findings show that CD49b^+^ are mainly defining L-bi cells and reconcile previous studies by showing that CD49b marks both HSCs and ST/transient cells.

The CD49b^−^ subset was able to efficiently generate all other HSC subsets. The CD49b^+^ subset was less effective in generating both stem- and progenitor cells, but was nevertheless still capable of giving rise to all phenotypically defined HSC subsets. This suggested that CD49b^−^ are hierarchically superior to CD49b^+^ cells, but also that a degree of interconversion may occur, which remains to be confirmed through functional analysis.

Our data indicated that a degree of functional heterogeneity remains, especially within the CD49b^+^ subset. Although we were unable to detect any significant functional differences *in vitro* with CD41 and CD229 subfractionation, it remains to be determined whether these subfractions can resolve the residual functional heterogeneity *in vivo*.

Insights into the molecular mechanisms underlying HSC heterogeneity are largely lacking. Genome-wide expression analysis of the HSC population has previously shown heterogeneity among phenotypic HSCs, suggesting that transcriptional profiling may distinguish functionally diverse HSC subsets (Challen et al., 2010; Haas et al., 2018; Wilson et al., 2015). Surprisingly, we observed high transcriptional overlap on both the bulk and the single-cell level between functionally different CD49b^−^ and CD49b^+^ cells. These findings suggested that functional differences between the HSC subsets may be determined by only a few genes or that RNA-seq could not reveal combinatorial consequences of small gene expression changes. To investigate the epigenetic changes associated with the subtle gene expression differences, we surveyed the genome-wide chromatin accessibility landscape of CD49b^−^ and CD49b^+^ cells. We observed distinct profiles, which, in agreement with previous studies, differed predominantly in promoter distal regions (Martin et al., 2021; Yu et al., 2017). We found a general increase in open chromatin associated with processes of an activated and proliferative cellular state in CD49b^+^. Conversely, in CD49b^−^ cells, open chromatin regions were associated with processes involved in quiescence and dormancy. These results implied that CD49b^−^ and CD49b^+^ cells may have different epigenetic configurations priming them for the distinct *in vivo* functional behavior. These findings highlight the need to unfold specific regulators and epigenetic mechanisms that directly affect HSC function and diversity.

Collectively, we have shown that CD49b can be used to further enrich LT M-bi HSCs to high purity, by segregating a subset of multipotent CD49b^+^ cells with lymphoid bias. Although L-bi cells were commonly associated with finite self-renewal, a small number of them could sustain LT, which correlated with the persistence of platelet and erythroid repopulation. Despite diverse functional characteristics, CD49b^−^ and CD49b^+^ HSCs were transcriptionally similar but epigenetically different. Overall, our studies highlight the different facets of the complex structure of the HSC compartment, composed of diverse HSCs with distinct functional behaviors that are likely regulated through epigenetic mechanisms as they sustain life-long hematopoiesis.

## Experimental procedures

See supplemental information for details.

### Animals

Female and male C57BL/6J mice (8–17 weeks) were used. Gata-1 eGFP (Drissen et al., 2016) mice were backcrossed more than eight generations to a C57BL/6J background. All experiments were approved by the regional ethics committee.

### Hematopoietic cell preparation

BM cell suspensions were prepared by bone crushing. Cells were Fc-blocked and stained with antibodies against cell-surface antigens (Table S3). For HSC detection, BM cells were enriched by CD117 immunomagnetic separation (Miltenyi Biotec) before antibody staining. Platelets, erythrocytes, and leukocytes were isolated from PB samples followed by antibody staining as described previously (Carrelha et al., 2018; Luc et al., 2016). See Table S4 for immunophenotypes.

### Transplantation experiments

Test cells were intravenously injected into lethally irradiated CD45.1 mice with 200,000–250,000 BM CD45.1^+^ support cells. PB analysis was periodically done 6–9 months post-transplantation.

### *In vitro* assays

Myeloid and B cell potential was evaluated with OP9 co-culture assay (Luc et al., 2012). Megakaryocyte potential was evaluated by manually plating one cell/well into 60-well plates and assessed after 13 days. Erythroid potential was evaluated by plating 30 HSCs in complete methylcellulose (GF M3434; STEMCELL Technologies) and evaluated after 12 days with 2,7-diaminofluorene staining (Merck) (Luc et al., 2012). Cell division kinetics was assessed by tracking cell divisions of single cells on days 1–4 post-sorting (Luc et al., 2016). See Table S5 for culture conditions.

### Cell-cycle and proliferation assays

Ki-67 staining was done with a Cytofix/Cytoperm Kit (BD Biosciences). One dose of BrdU was given by intraperitoneal injection (50 μg/g body weight, BD), followed by oral administration (800 μg/mL, Merck) for 3 days. BrdU visualization was performed with a BrdU Flow Kit (BD Biosciences).

### RNA-seq and ATAC-seq

Bulk RNA-seq was performed with 250–500 sorted cells. Libraries were paired-end sequenced (2 × 41 cycles) on NextSeq 500 (Illumina). scRNA-seq, with SmartSeq2, was performed by sorting single cells into 384-well plates, and libraries were sequenced on HiSeq3000 (Illumina) using dual indexing and single 50-bp reads as previously described (Vanlandewijck and Betsholtz, 2018).

ATAC-seq was performed with 500 sorted cells using the Omni-ATAC protocol with modifications (Corces et al., 2017). Libraries were paired-end sequenced (2 × 41 cycles) using NextSeq 550 (Illumina).

### Statistical methods

Statistical analysis was done in GraphPad Prism v.9.2.0. Non-parametric tests were performed using the Mann-Whitney test or the Kruskal-Wallis with Dunn’s multiple comparison test. The means ± SD are shown.

## Author contributions

E.S., A.-S.J., Ö.D., and S.L. performed cell and mouse experiments. P.K. performed ATAC-seq. N.S.F. and C.G. performed RNA-seq. G.M. and M.V. performed and analyzed scRNA-seq. J.H. performed bioinformatics analysis. T.-Y.S., H.B., and C.L.T. assisted in experiments. S.L. designed the project and wrote the paper together with R.M., E.S., and J.H. S.H.O., R.M., and S.L. supervised the work. All authors reviewed the manuscript before submission.

## Conflicts of interests

The authors declare no competing interests.

## Data Availability

RNA-seq and ATAC-seq data have been deposited in the European Nucleotide Archive (ENA). The accession number for the RNA-seq and ATAC-seq data reported in this paper is ENA: PRJEB47791.

## Supplemental information

Document S1. Figures S1–S7, Tables S3–S5, and supplemental experimental procedures

Table S1. Bulk RNA-seq samples, related to Figure 6

Table S2. ATAC-seq samples, related to Figure 6

Document S2. Article plus supplemental information
