# Supplementary material for: CD49b identifies functionally and epigenetically distinct subsets of lineage-biased hematopoietic stem cells
Source: Stem Cell Reports. 2022 Jun 16;17(7):1546–60. doi: 10.1016/j.stemcr.2022.05.014 (PMC9287668; doi:10.1016/j.stemcr.2022.05.014)
Supplement: Document S1. Figures S1–S7, Tables S3–S5, and supplemental experimental procedures [file mmc1.pdf]

Stem Cell Reports, Volume *17*

Supplemental Information

CD49b identifies functionally and epigenetically distinct subsets of lineage-biased hematopoietic stem cells

Ece Somuncular, Julia Hauenstein, Prajakta Khalkar, Anne-Sofie Johansson, Özge Dumral, Nicolai S. Frengen, Charlotte Gustafsson, Giuseppe Mocci, Tsu-Yi Su, Hugo Brouwer, Christine L. Trautmann, Michael Vanlandewijck, Stuart H. Orkin, Robert Månsson, and Sidinh Luc

Figure S1, related to Figure 1

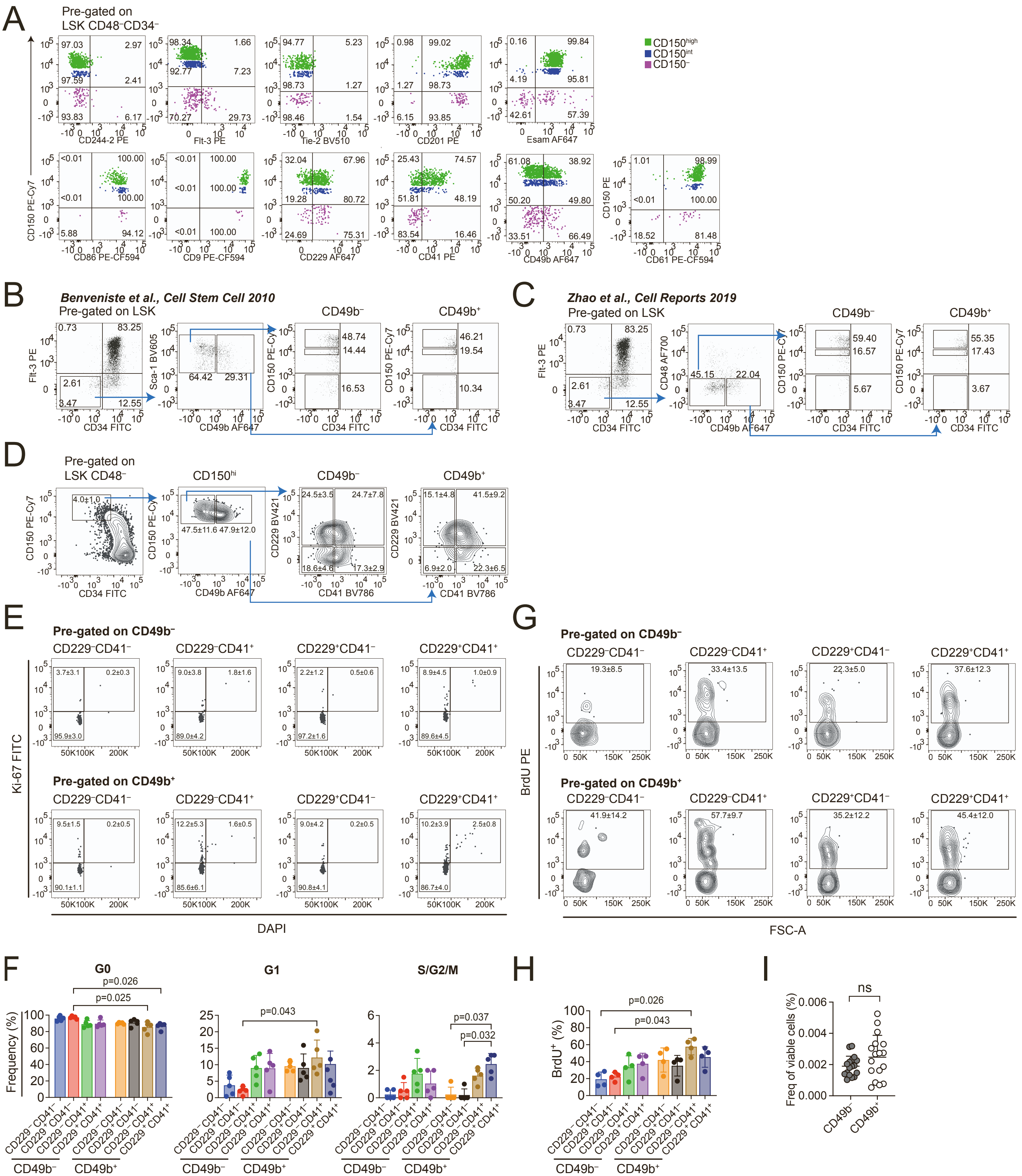

Figure S1, related to Figure 1

The hematopoietic stem cell compartment can be phenotypically subfractionated with CD49b

(A) Flow cytometry profiles of cell surface markers tested for further separation of phenotypic hematopoietic stem cell subsets defined as $Lineage^{–}Sca\text{-}1^{+}c\text{-}Kit^{+}$ (LSK) $CD48^{–}CD34^{–}CD150^{high}$ ($CD150^{hi}$), $LSKCD48^{–}CD34^{–}CD150^{intermediate}$ ($CD150^{int}$) and $LSKCD48^{–}CD34^{–}CD150^{–}$ ($CD150^{–}$), from c-Kit-enriched BM cells. All plots have been pre-gated on LSK $CD48^{–}CD34^{–}$ cells. The tested markers are shown on the x-axis. Frequencies of parent gates are shown.

(B) Flow cytometry profiles and gating strategy as shown in Benveniste *et al.* of long-term HSCs ($Lin^{–}Sca\text{-}1^{+}c\text{-}Kit^{+}CD34^{–}Flt\text{-}3^{–}CD49b^{–}$) and intermediate-term HSCs ($Lin^{–}Sca\text{-}1^{+}c\text{-}Kit^{+}CD34^{–}Flt\text{-}3^{–}CD49b^{+}$) and the subsequent analysis of CD150 expression.

(C) Flow cytometry profiles and gating strategy as shown in Zhao *et al.* of reserve HSCs ($Lin^{–}Sca\text{-}1^{+}c\text{-}Kit^{+}CD34^{–}Flt\text{-}3^{–}CD48^{–}CD49b^{–}$) and primed HSCs ($Lin^{–}Sca\text{-}1^{+}c\text{-}Kit^{+}CD34^{–}Flt\text{-}3^{–}CD48^{–}CD49b^{+}$) and the subsequent analysis of CD150 expression.

(D) Flow cytometry profiles showing further separation of $CD49b^{–}$ and $CD49b^{+}$ subsets with CD229 and CD41. Frequencies from parent gates are shown as mean ± SD from two experiments.

(E) Flow cytometry profiles of cell cycle analysis by Ki-67 staining. Frequencies are shown as mean ± SD from 5 biological replicates.

(F) Frequency of cells in G0, G1 and S/G2/M. Data collected from 5 biological replicates, one experiment.

(G) Flow cytometry profiles of BrdU labeling. Frequencies are shown as mean ± SD from 4 biological replicates.

(H) Frequency of proliferating ($BrdU^{+}$) cells. Frequencies are shown as mean ± SD from 4 biological replicates, one experiment.

(I) Total frequency of $CD49b^{–}$ and $CD49b^{+}$ HSCs in unfractionated BM (n = 17 mice, from 3 independent experiments). Data is presented as means ± SD. The statistical analysis was performed with Kruskal-Wallis with Dunn's multiple comparison test in (C) and (E) and Mann-Whitney test in (I). ns = not significant.

Figure S2, related to Figure 3

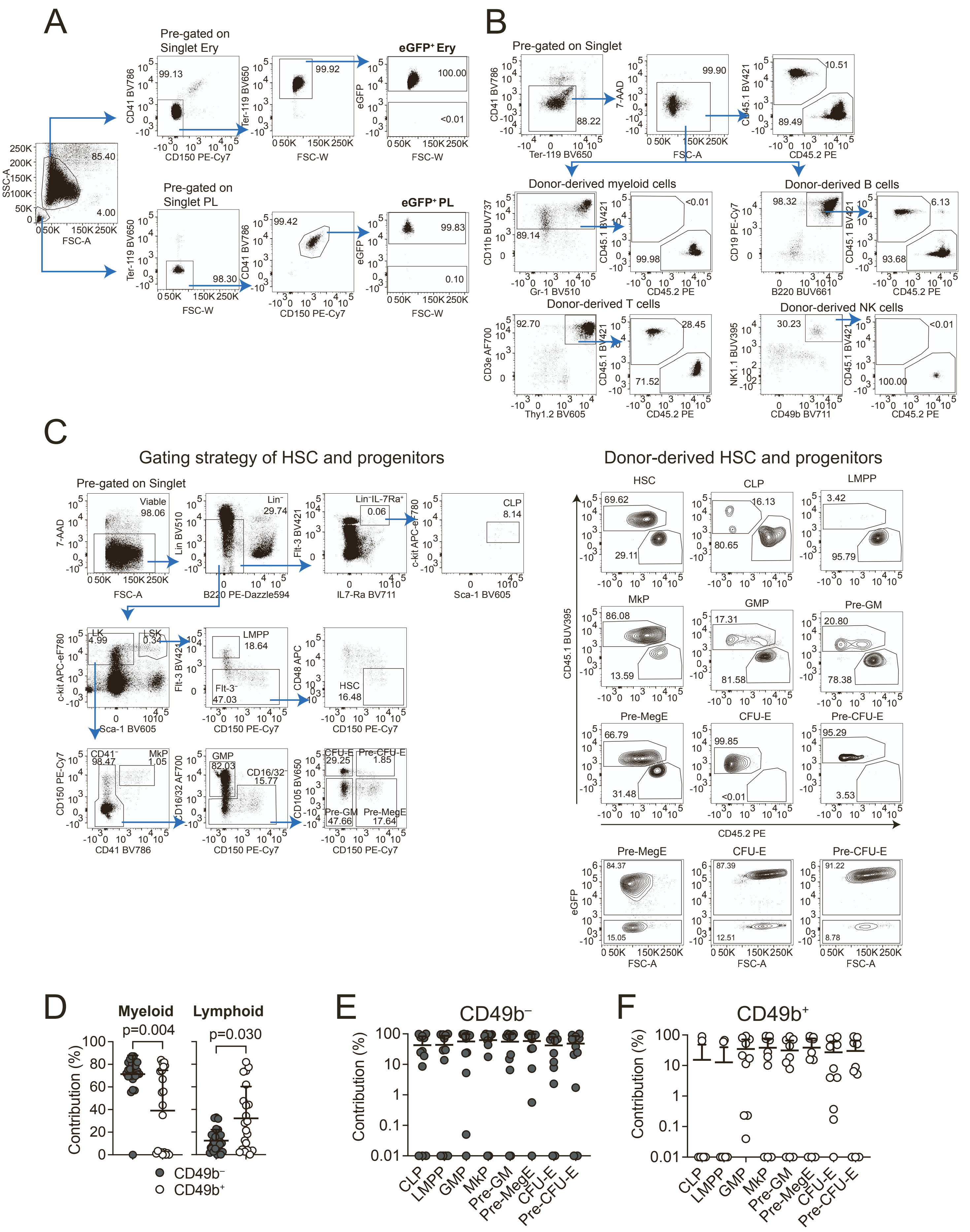

Figure S2, related to Figure 3

Analysis of hematopoietic populations from CD49b^{-} and CD49b^{+} transplanted mice

(A) Flow cytometry profiles and gating strategy for platelet-erythrocyte analysis from a representative peripheral blood sample. Donor-derived platelets (PL) were phenotypically defined as Ter-119^{-}CD41^{+}CD150^{+}*Gata-1*-eGFP^{+} and erythrocytes (Ery) as CD41^{-}CD150^{-}Ter-119^{+}*Gata-1*-eGFP^{+}. Frequencies of parent gates are shown.

(B) Flow cytometry profiles and gating strategy for mature blood lineage analysis in peripheral blood and BM. A representative peripheral blood sample is shown. Total donor-derived cells were phenotypically defined as Ter-119^{-}CD41^{-}CD45.2^{+}, donor-derived myeloid cells as Ter-119^{-}CD41^{-}B220^{-}CD19^{-}NK1.1^{-}CD49b^{-}CD3e^{-}Thy1.2^{-}CD11b^{+}CD45.2^{+}, donor-derived B cells as Ter-119^{-}CD41^{-}CD11b^{-}Gr-1^{-}CD3e^{-}Thy1.2^{-}NK1.1^{-}CD49b^{-}B220^{+}CD19^{+}CD45.2^{+}, donor-derived T cells as Ter-119^{-}CD41^{-}CD11b^{-}Gr-1^{-}B220^{-}CD19^{-}NK1.1^{-}CD49b^{-}CD3e^{+}Thy1.2^{+} CD45.2^{+} and donor-derived NK cells as Ter-119^{-}CD41^{-}CD11b^{-}Gr-1^{-}B220^{-}CD19^{-}CD3e^{-} Thy1.2^{-}NK1.1^{+}CD49b^{+}CD45.2^{+}. Frequencies of parent gates are shown.

(C) Flow cytometry profiles and gating strategy for HSC and progenitor analysis from two representative unfractionated BM samples. A representative mouse showing gating strategy and donor reconstitution with CD45.2^{+}, and a second mouse demonstrating donor reconstitution of Pre-Meg-E, and Pre-CFU-E, CFU-E with Gata-1 eGFP^{+} expression. Donor-derived common lymphoid progenitor (CLP) were phenotypically defined as Lin^{-} B220^{low}Sca-1^{+}c-Kit^{+}Flt-3^{+}IL-7Ra^{+}CD45.2^{+}, lymphoid-primed multipotent progenitor (LMPP) as Lin^{-}Sca-1^{+}c-Kit^{+} (LSK) Flt-3^{hi}CD45.2^{+}, megakaryocyte progenitor (MkP) as Lin^{-}Sca-1^{-}c-Kit^{+} (LK) CD150^{+}CD41^{+}CD45.2^{+}, granulocyte-monocyte progenitor (GMP) as LKCD41^{-}CD150^{-}CD16/32^{+} CD45.2^{+}, Pre-granulocyte-monocyte (Pre-GM) as LKCD41^{-}CD16/32^{-}CD150^{-}CD105^{-}CD45.2^{+}, Pre-megakaryocyte-erythrocyte (Pre-MegE) as LKCD41^{-}CD16/32^{-}CD150^{+}CD105^{-}CD45.2^{+} or alternatively as LKCD41^{-}CD16/32^{-}CD150^{+}CD105^{-}eGFP^{+}, Pre-colony forming unit erythroid (Pre-CFU-E) as LKCD41^{-}CD16/32^{-}CD150^{+}CD105^{+}CD45.2^{+} or alternatively as LKCD41^{-}CD16/32^{-}CD150^{+}CD105^{+} eGFP^{+}, Colony forming unit erythroid (CFU-E) as LKCD41^{-}CD16/32^{-}CD150^{-}CD105^{+}CD45.2^{+} or alternatively as LKCD41^{-}CD16/32^{-}CD150^{-}CD105^{+}eGFP^{+}, Hematopoietic stem cell (HSC) as LSKFlt-3^{-} CD48^{-}CD150^{+}CD45.2^{+} or alternatively LSKCD48^{-}CD150^{+}CD45.2^{+}. Frequencies of parent gates are shown.

(D) Frequency of donor contribution to myeloid cells and lymphoid cells (B cells, T cells and NK cells) in the BM, 5-6 months following transplantation of 5 cells of CD49b^{-} and CD49b^{+} HSC populations ($n_{CD49b^{-}}$ = 27 mice and $n_{CD49b^{+}}$ = 21 mice, from 3 experiments).

(E-F) Frequency of donor contribution to progenitor cells, CLP, LMPP, GMP, MkP, PreGM, PreMegE, CFU-E and Pre-CFU-E in the BM, 5-6 months following transplantation of 5 cells of CD49b^{-} (E) and CD49b^{+} (F) HSC populations ($n_{CD49b^{-}}$ = 12 mice and $n_{CD49b^{+}}$ = 10 mice from 2 experiments).

Data in (D-F) are presented as means $\pm$ SD. The statistical analysis was performed with Mann-Whitney test in (D).

Figure S3, related to Figure 4

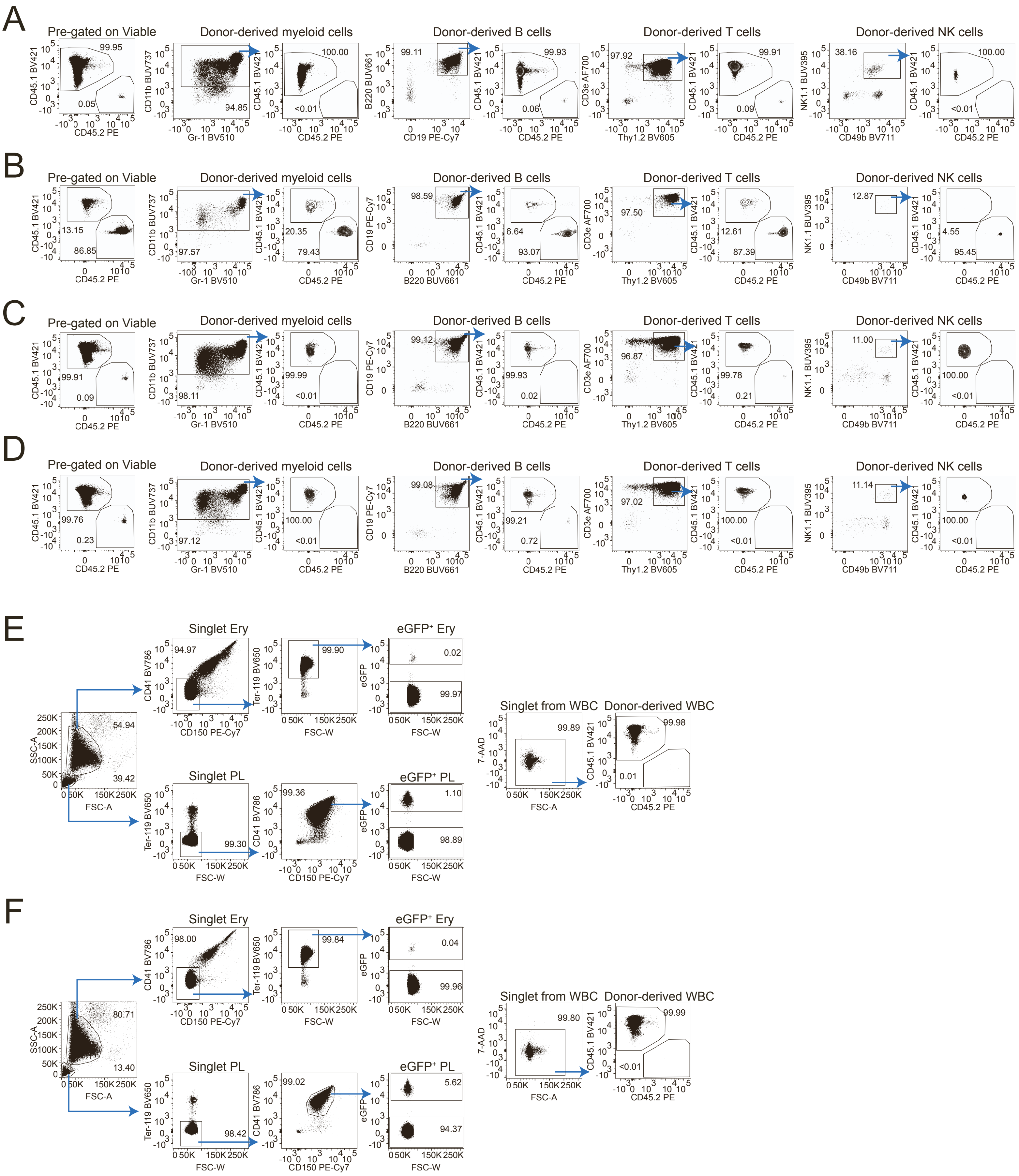

Figure S3, related to Figure 4

Examples of positively reconstituted mice in in single cell transplantation analyses

(A-D) Flow cytometry profiles of mature blood lineage analysis in peripheral blood showing representative single cell transplanted mice, defined as positively reconstituted but with different reconstitution profiles.

(A) A transplanted mouse with low reconstitution level to only B and T cells (see mouse ID = 014_013 in Figure S4B).

(B) A transplanted mouse with high reconstitution level to myeloid cells, B cells, T cells and NK cells (see mouse ID = 036_004 in Figure S4A).

(C) A transplanted mouse with low reconstitution level to only T cells (see mouse ID = 036_019 in Figure S4B).

(D) A transplanted mouse with low reconstitution level to only B cells, (see mouse ID = 036_044 in Figure S4B).

(E-F) Flow cytometry profiles of platelet-erythrocyte and white blood cell (WBC) analysis showing single cell transplanted mice, which were positively reconstituted for platelets and erythrocytes only. Mouse ID = 033_005 (E) and mouse ID = 033_037 (F) from Figure S4A are shown.

Total donor-derived cells were phenotypically defined as Ter-119$^-$CD41$^-$CD45.2$^+$, donor-derived B cells as Ter-119$^-$CD41$^-$CD11b$^-$Gr-1$^-$CD3e$^-$Thy1.2$^-$NK1.1$^-$CD49b$^-$B220$^+$CD19$^+$ CD45.2$^+$, donor-derived NK cells as Ter-119$^-$CD41$^-$CD11b$^-$Gr-1$^-$B220$^-$CD19$^-$CD3e$^-$Thy1.2$^-$ NK1.1$^+$CD49b$^+$CD45.2$^+$, donor-derived T cells as Ter-119$^-$CD41$^-$CD11b$^-$Gr-1$^-$B220$^-$CD19$^-$ NK1.1$^-$CD49b$^-$CD3e$^+$Thy1.2$^+$CD45.2$^+$ and donor-derived myeloid cells as Ter-119$^-$CD41$^-$ B220$^-$CD19$^-$NK1.1$^-$CD49b$^-$CD3e$^-$Thy1.2$^-$CD11b$^+$ CD45.2$^+$. Donor-derived platelets (PL) were phenotypically defined as Ter-119$^-$CD41$^+$CD150$^+$ *Gata-1*-eGFP$^+$ and erythrocytes (Ery) as CD41$^-$CD150$^-$Ter-119$^+$*Gata-1*-eGFP$^+$. Frequencies of parent gates are shown.

Figure S4, related to Figure 4 and 5

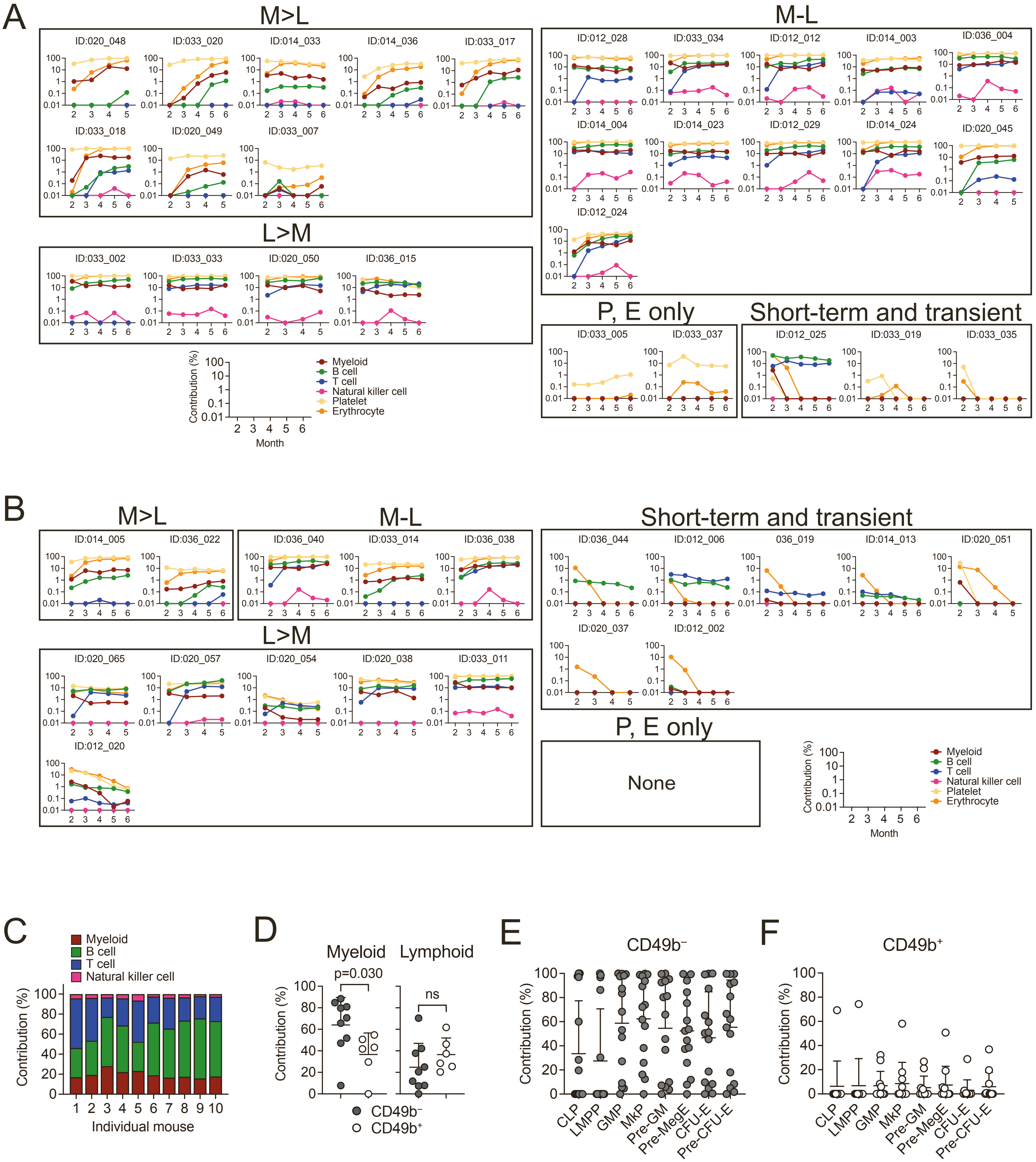

Figure S4, related to Figures 4 and 5

Repopulation profiles of single cell transplanted mice

(A-B) Repopulation patterns of single cell transplanted mice with LSKCD48^{-}CD34^{-}CD150^{hi} CD49b^{-} (CD49b^{-}) cells in (A) and LSKCD48^{-}CD34^{-}CD150^{hi}CD49b^{+} (CD49b^{+}) cells in (B), showing frequency of total donor contribution to myeloid, B cell, T cell, natural killer cell, platelet, and erythrocyte lineages in peripheral blood up to 6 months post-transplantation. Single cell transplanted mice were grouped according to their repopulation profiles, into myeloid reconstitution higher than lymphoid reconstitution (M>L), similar reconstitution levels of myeloid and lymphoid (M-L), lymphoid reconstitution higher than myeloid reconstitution (L>M), platelet and erythrocyte only reconstitution (P, E only) and short-term and transient reconstitution.

(C) Blood cell composition in the peripheral blood of 12-18-week-old unmanipulated mice (*n* = 10 mice from 7 experiments).

(D) Frequency of donor contribution to myeloid cells and lymphoid cells (B cells, T cells and NK cells) in the BM, 5-6 months following single cell transplantation of CD49b^{-} and CD49b^{+} cells ($n_{CD49b^{-}}$ = 9 mice and $n_{CD49b^{+}}$ = 6 mice, from 2 independent experiments).

(E-F) Frequency of donor contribution to progenitor cells, CLP, LMPP, GMP, MkP, PreGM, PreMegE, CFU-E and Pre-CFU-E in the BM, 5-6 months following single cell transplantation of CD49b^{-} (E) and CD49b^{+} (F) cells ($n_{CD49b^{-}}$ = 15 mice and $n_{CD49b^{+}}$ = 11 mice, from 3 independent experiments).

Data in (D-F) are presented as means ± SD. Frequencies of parent gates are shown in FACS plots. Statistical analysis was performed with a Mann-Whitney test in (D). ns = not significant.

Figure S5, related to Figure 4 and 5

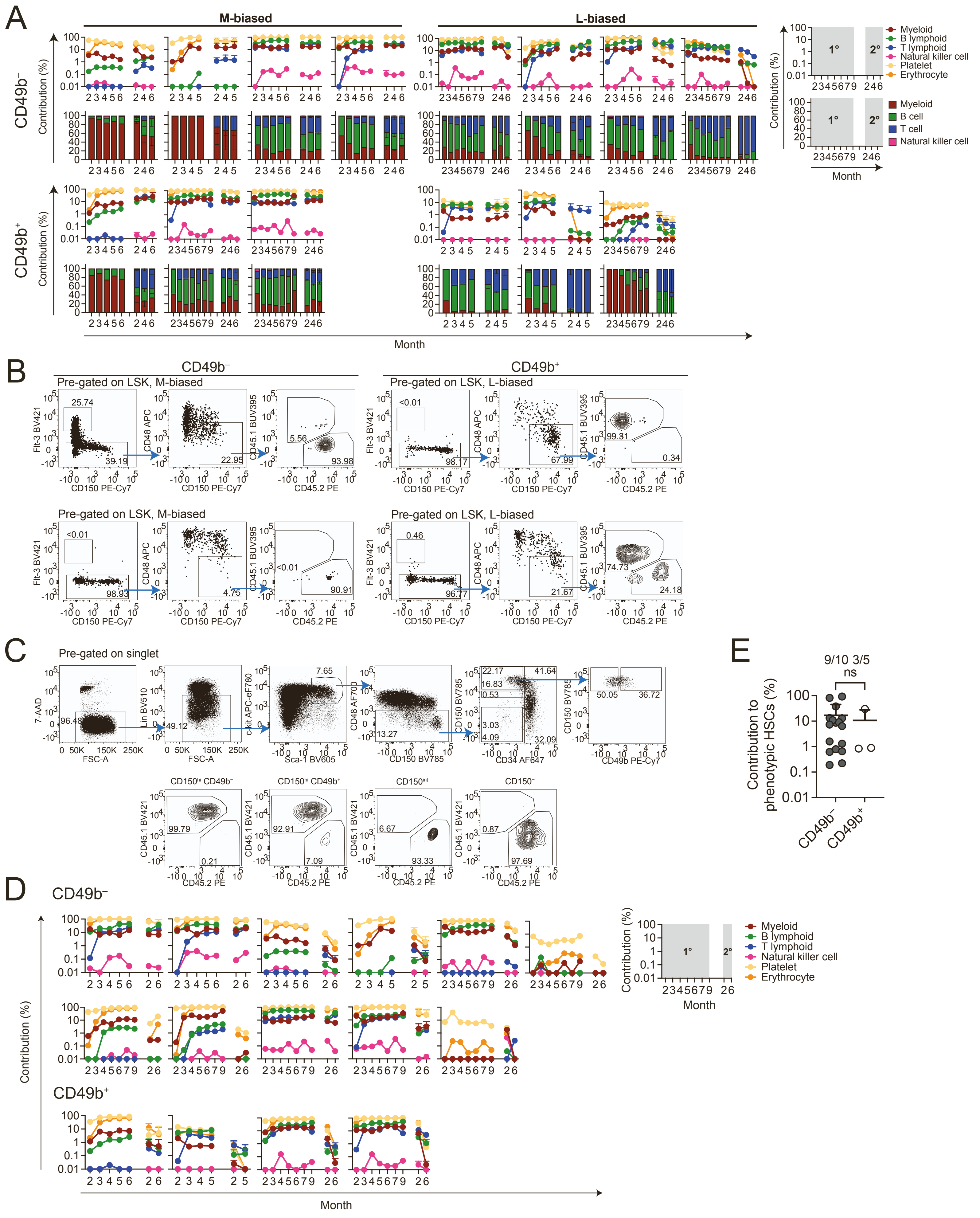

Figure S5, related to Figures 4 and 5

Repopulation of blood and bone marrow hematopoietic cells in primary and secondary single cell transplantations

(A) Frequency of total donor contribution to myeloid, B cell, T cell, natural killer cell, platelet and erythrocyte lineages in peripheral blood (top) and their corresponding relative contribution to M, B, T and NK within the donor leukocyte fraction (bottom), up to 5-9 months following single-cell transplantation of $CD49b^{-}$ and $CD49b^{+}$ populations, as well as secondary transplantation. Secondary transplantation was done with 10 million whole BM cells from the primary donor, transplanted into 1-5 secondary recipients per primary donor. Each graph represents data from one individual mouse in primary transplantation, with myeloid bias (M-biased) or lymphoid bias (L-biased) as indicated.

(B) Flow cytometry profiles of donor-derived phenotypic hematopoietic stem cells (HSC) in BM, from representative M-biased or L-biased secondary transplanted mice. Donor-derived phenotypic HSCs were defined as LSKFlt-3^{-}CD48^{-}CD150^{+}CD45.2^{+}. FACS plots were pre-gated on LSK. Frequencies of parent gates are shown. On average 2 million events were recorded per sample and ≥10 events were used to determine whether mice were positively reconstituted for HSCs.

(C) Flow cytometry profiles and gating strategy for phenotypic lineage-biased HSC subsets, from a representative c-Kit-enriched BM sample. Donor-derived HSC subsets were phenotypically defined as LSKCD48^{-}CD34^{-}CD150^{hi}CD$49b^{-}$CD45.2^{+}, LSKCD48^{-}CD34^{-}CD150^{hi}CD$49b^{+}$CD45.2^{+}, LSKCD48^{-}CD34^{-}CD150^{int}CD45.2^{+} and LSKCD48^{-}CD34^{-}CD150^{-} CD45.2^{+}.

(D) Frequency of total donor contribution to myeloid, B cell, T cell, natural killer cell, platelet and erythrocyte lineages in peripheral blood, in single-cell transplantation of $CD49b^{-}$ and $CD49b^{+}$ populations, as well as secondary transplantation, up to 5-9 months post-transplantation. Secondary transplantation was done with 30-100 sorted donor-derived $CD49b^{-}$ or $CD49b^{+}$ cells per secondary recipient and 1-5 secondary recipients per primary donor. Each graph represents data from one individual mouse from primary transplantation.

(E) Donor contribution to phenotypic HSCs (LSKCD48^{-}CD150^{+}) in secondary transplantation of 30-100 sorted $CD49b^{-}$ and $CD49b^{+}$ cells, 5-6 months post-transplantation ($n_{CD49b^{-}}$ = 17, $n_{CD49b^{+}}$ = 3 mice, 5 independent experiments). The number of reconstituted primary donor mice out of all mice analyzed is indicated above the graph.

Data in (A, top), (D) and (E) are presented as means ± SD. Frequencies of parent gates are shown in FACS plots. Statistical analysis was performed with a Mann-Whitney test in (E). ns = not significant.

Figure S6, related to Figure 6

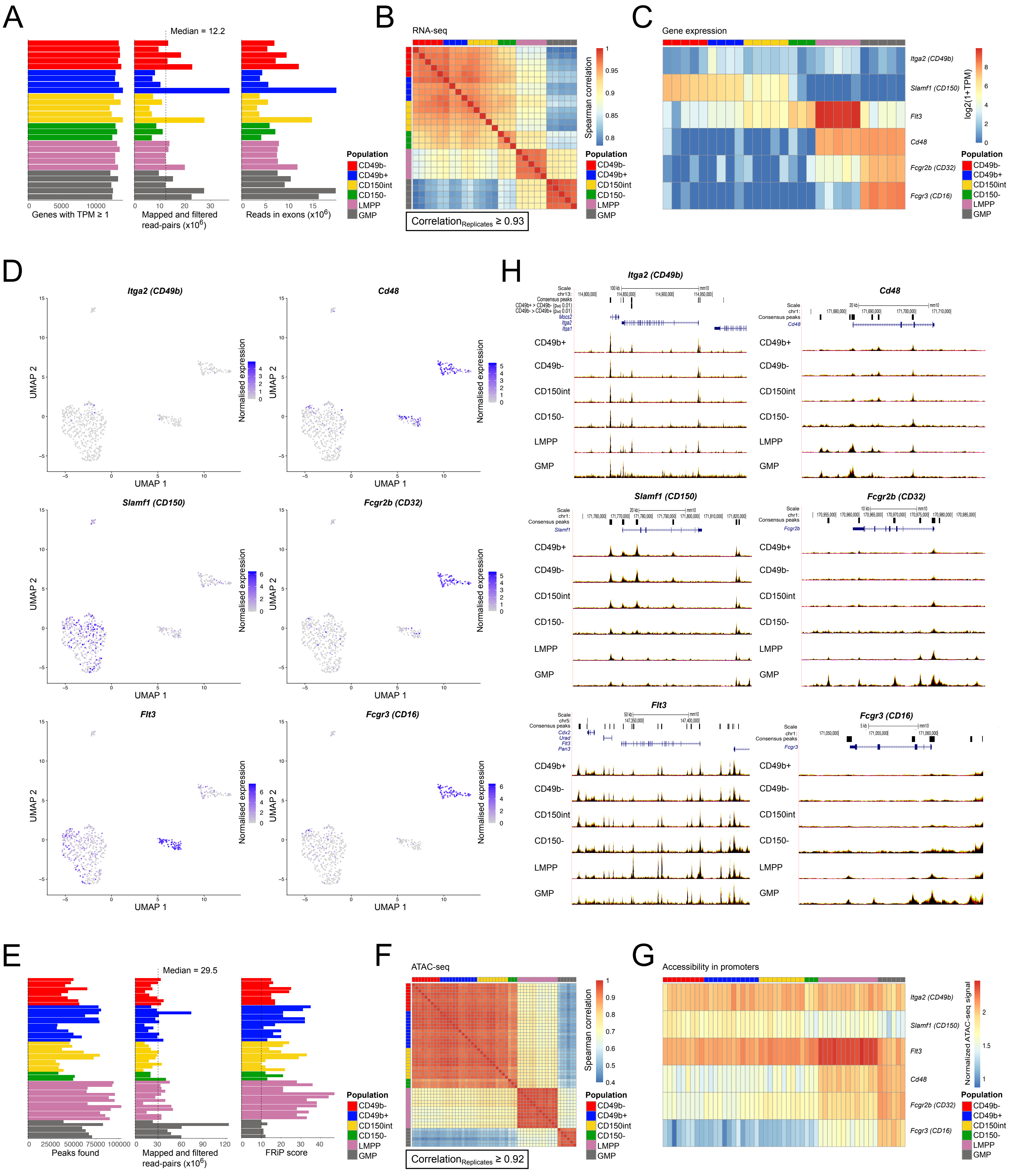

Figure S6, related to Figure 6

RNA- and ATAC-sequencing samples, and gene expression and chromatin accessibility of selected genes

(A) Distribution of number of expressed genes (≥1 TPM), number of mapped and filtered read-pairs and number of reads in exons among individual RNA-seq samples.

(B) Spearman correlation between RNA-seq samples. Expressed genes with ≥1 TPM in at least 3 samples are included. Correlation is calculated on $\log_{10}$ transformed and quantile normalized data.

(C) Heatmap of gene expression for selected, characteristic surface markers for the studied cell populations by bulk RNA-seq analysis.

(D) UMAP visualization of gene expression of selected, characteristic surface markers by single cell RNA-seq. The color intensity represents the expression level.

(E) Distribution of number of found consensus peaks, number of mapped and filtered read-pairs and fraction of reads in peaks (FRiP) score among individual ATAC-seq samples.

(F) Spearman correlation between ATAC-seq samples. Peaks with a normalized read count >1.5 in more than one third of any population are included. Correlation is calculated on $\log_{10}$ transformed and quantile normalized data.

(G) Heatmap of chromatin accessibility in the promoter (based on the GENCODE VM23 Comprehensive Transcript Set) of selected, characteristic surface markers for the studied cell populations. Signal corresponds to $\log_{10}$ transformed and quantile normalized read counts.

(H) UCSC browser tracks of ATAC-seq signal for selected, characteristic surface markers for the studied cell populations. Signal was scaled to 1 million mapped reads in peaks and for each cell population tracks of the median signal (± SD) are shown.

Figure S7, related to Figure 6 and 7

A

Gene name	Up in	log_2 fold change	adjusted p-value
Slc16a1	CD49b+	-2.09	0.002
Iqgap3	CD49b+	-1.91	0.007
Galnt6	CD49b+	-1.04	0.007
Mxd4	CD49b+	-2.36	0.020
6330416G13Rik	CD49b+	-3.38	0.046
Gfi1	CD49b-	3.21	0.025
Rrp12	CD49b-	3.12	0.037
Lrrc20	CD49b-	1.93	0.040

B

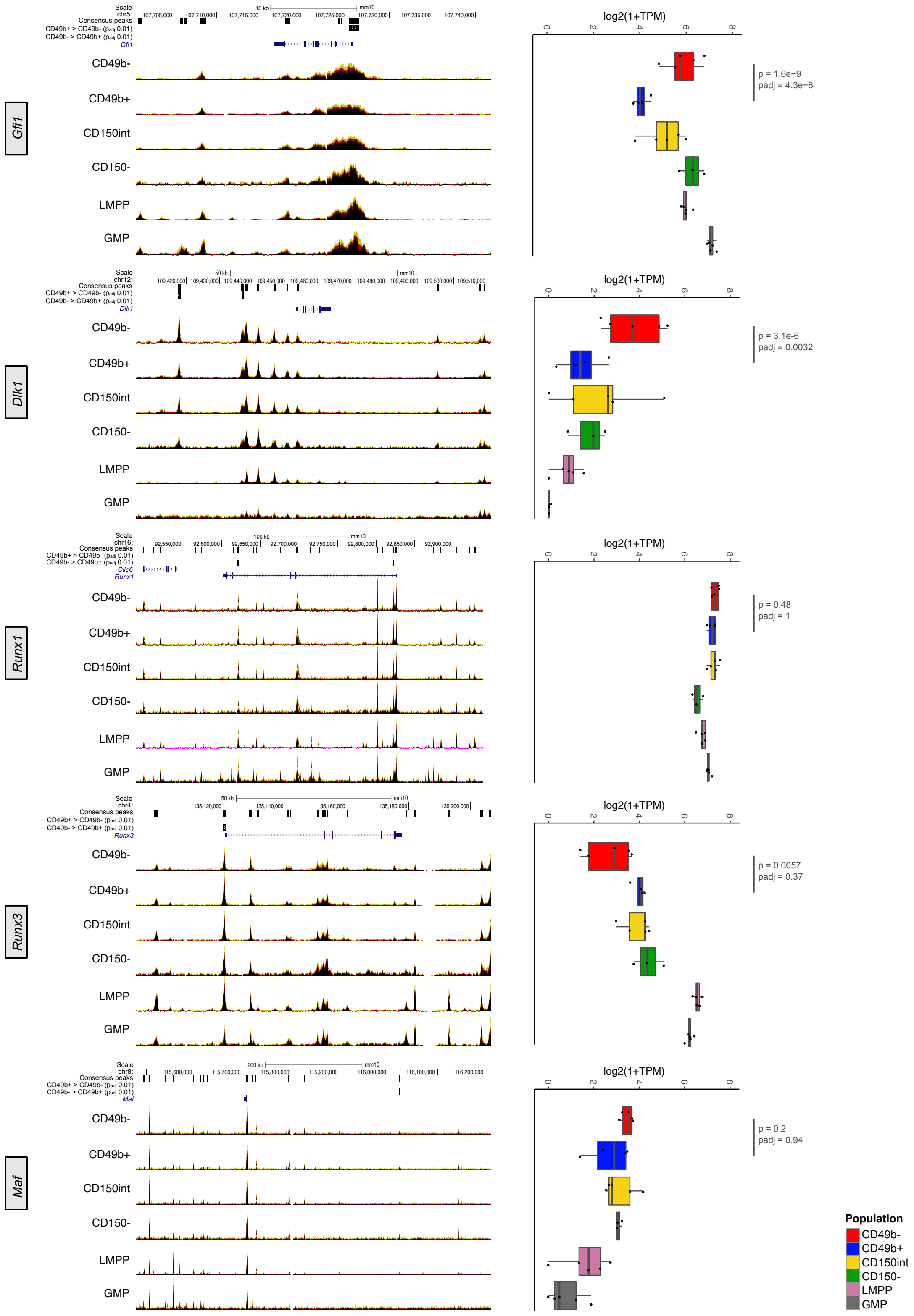

Figure S7, related to Figures 6 and 7

Differential gene expression and chromatin accessibility of select genes

(A) Differential expression analysis between $CD49b^-$ and $CD49b^+$ cells using the likelihood-ratio test for scRNA-seq data (logFC > 1, padj < 0.05).

(B) UCSC browser tracks of ATAC-seq signal for selected regions with differential accessibility between $CD49b^-$ and $CD49b^+$ cells (left) and RNA expression of the genes proximal to the differential regions (right). RNA expression in individual samples is shown as dots and boxplots show the distribution in each population.

Table S3. Antibody list, related to Figures 1-7 and Experimental procedures

ANTIBODIES	SOURCE	IDENTIFIER
Anti-mouse CD105 (Endoglin) BV650 (clone MJ7/18)	BD Biosciences	Cat#: 740609
Anti-mouse CD117 (c-kit) APC-eF780 (clone 2B8)	ThermoFisher Scientific	Cat#: 47-1171-82
Anti-mouse CD11b (Mac-1) BUV395 (clone M1/70)	BD Biosciences	Cat#: 563553
Anti-mouse CD11b (Mac-1) BUV737 (clone M1/70)	BD Biosciences	Cat#: 564443
Anti-mouse/Human CD11b (Mac-1) BV510 (clone M1/70)	BioLegend	Cat#: 101263
Anti-mouse CD11b (Mac-1) eF450 (clone M1/70)	ThermoFisher Scientific	Cat#: 48-0112-82
Anti-mouse CD11b (Mac-1) PE (clone M1/70)	BD Biosciences	Cat#: 553311
Anti-mouse CD127 (IL7-Ra) BV711 (clone A7R34)	BioLegend	Cat#: 135035
Anti-mouse CD135 (Flt-3) PE (clone A2F10)	BioLegend	Cat#: 135306
Anti-mouse CD135 (Flt-3) BV421 (clone A2F10.1)	BD Biosciences	Cat#: 562898
Anti-mouse CD150 (SLAM) BV785 (clone TC15-12F12.2)	BioLegend	Cat#: 115937
Anti-mouse CD150 (SLAM) PE (clone TC15-12F12.2)	BioLegend	Cat#: 115904
Anti-mouse CD150 (SLAM) PE-Cy7 (clone TC15-12F12.2)	BioLegend	Cat#: 115914
Anti-mouse CD16/32 AF700 (clone 93)	ThermoFisher Scientific	Cat#: 56-0161-82
Anti-mouse CD16/32 (Fc-block) Purified (clone 2.4G2)	BD Biosciences	Cat#: 553142
Anti-mouse CD19 PE-Cy7 (clone 6D5)	BioLegend	Cat#: 115520
Anti-mouse CD19 PerCP-Cy5.5 (clone 1D3)	ThermoFisher Scientific	Cat#: 45-0193-82
Anti-mouse CD201 (EPCR) PE (clone eBio1560)	ThermoFisher Scientific	Cat#: 12201282
Anti-mouse CD202b (Tie-2) Biotin (clone TEK4)	BioLegend	Cat#: 124006
Anti-mouse CD229 (Ly9) APC (clone Ly9ab3)	BioLegend	Cat#: 122907
Anti-mouse CD244.2 (2B4) PE (clone eBio244F4)	ThermoFisher Scientific	Cat#: 12-2441-82
Anti-mouse CD34 FITC (clone RAM34)	ThermoFisher Scientific	Cat#: 11-0341-85
Anti-mouse CD34 AF647 (clone RAM34)	BD Biosciences	Cat#: 560230
Anti-mouse CD3e AF700 (clone 500A2)	BD Biosciences	Cat#: 557984
Anti-mouse CD3e BV510 (clone 145-2C11)	BioLegend	Cat#: 100353
Anti-mouse CD3e BUV395 (clone 145-2C11)	BD Biosciences	Cat#: 563565
Anti-mouse CD3e eF450 (clone 145-2C11)	ThermoFisher Scientific	Cat#: 48-0031-82
Anti-mouse CD4 BUV395 (clone RM4-5)	BD Biosciences	Cat#: 740208
Anti-mouse CD41 BV786 (clone MWReg30)	BD Biosciences	Cat#: 740903
Anti-mouse CD41 PE (clone eBioMWReg30)	ThermoFisher Scientific	Cat#: 12-0411-83
Anti-mouse CD45.1 (Ly5.1) BV421 (clone A20)	BD Biosciences	Cat#: 563983
Anti-mouse CD45.1 (Ly5.1) BUV395 (clone A20)	BD Biosciences	Cat#: 565212
Anti-mouse CD45.2 (Ly5.2) PE (clone 104)	BioLegend	Cat#: 109808
Anti-mouse CD45.2 (Ly5.2) APC-Fire750 (clone 104)	BioLegend	Cat#: 109852
Anti-mouse CD45R/B220 BUV395 (clone RA3-6B2)	BD Biosciences	Cat#: 563793
Anti-mouse CD45R/B220 BUV661 (clone RA3-6B2)	BD Biosciences	Cat#: 565077
Anti-mouse CD45R/B220 eF450 (clone RA3-6B2)	ThermoFisher Scientific	Cat#: 48-0452-82
Anti-mouse CD45R/B220 FITC (clone RA3-6B2)	BD Biosciences	Cat#: 553088
Anti-mouse/Human CD45R/B220 PE-Dazzle 594 (clone RA3-6B2)	BioLegend	Cat#: 103258

Anti-mouse/Human CD45R/B220 BV510 (clone RA3-6B2)	BioLegend	Cat#: 103248
Anti-mouse CD48 AF700 (clone HM48-1)	BioLegend	Cat#: 103426
Anti-mouse CD48 APC (clone HM48-1)	BioLegend	Cat#: 103412
Anti-mouse CD48 PerCP-Cy5.5 (clone HM48-1)	BioLegend	Cat#: 103422
Anti-mouse CD49b PE-Cy7 (clone HMα2)	BioLegend	Cat#: 103518
Anti-mouse CD49b AF647 (clone HMα2)	BioLegend	Cat#: 103511
Anti-mouse CD49b Biotin (clone HMa2)	ThermoFisher Scientific	Cat#: 13-0491-85
Anti-mouse CD49b BV711 (clone HMα2)	BD Biosciences	Cat#: 740704
Anti-mouse CD5 BV510 (clone 53-7.3)	BioLegend	Cat#: 100627
Anti-mouse CD5 BUV395 (clone 53-7.3)	BD Biosciences	Cat#: 740206
Anti-mouse CD5 eF450 (clone 53-7.3)	ThermoFisher Scientific	Cat#: 48-0051-82
Anti-mouse CD61 Biotin (clone 2C9.G2)	BD Biosciences	Cat#: 553345
Anti-mouse CD86 Biotin (clone GL-1)	BioLegend	Cat#: 105003
Anti-mouse CD8a BUV395 (clone 53-6.7)	BD Biosciences	Cat#: 563786
Anti-mouse CD9 Biotin (clone MZ3)	BioLegend	Cat#: 124803
Anti-mouse Esam APC (clone 1G8/ESAM)	BioLegend	Cat#: 136207
Anti-mouse F4/80 APC (clone BM8)	BioLegend	Cat#: 123116
Anti-mouse F4/80 APC (clone BM8)	ThermoFisher Scientific	Cat#: 17-4801-82
Anti-mouse Gr-1 BV510 (clone RB6-8C5)	BioLegend	Cat#: 108437
Anti-mouse Gr-1 BUV395 (clone RB6-8C5)	BD Biosciences	Cat#: 563849
Anti-mouse Gr-1 (Ly-6G and Ly-6C) APC (clone RB6-8C5)	BioLegend	Cat#: 108412
Anti-mouse Gr-1 (Ly-6G) eF450 (clone RB6-8C5)	ThermoFisher Scientific	Cat#: 48-5931-82
Anti-mouse Gr-1 (Ly-6G/Ly-6C) PE (clone RB6-8C5)	BD Biosciences	Cat#: 553128
Anti-mouse NK1.1 BUV395 (clone PK136)	BD Biosciences	Cat#: 564144
Anti-mouse NK1.1 FITC (clone PK136)	ThermoFisher Scientific	Cat#: 11-5941-82
Anti-mouse Sca-1 (Ly-6A/E) BV605 (clone D7)	BioLegend	Cat#: 108134
Anti-mouse Sca-1 (Ly-6A/E) AF700 (clone D7)	ThermoFisher Scientific	Cat#: 56-5981-82
Anti-mouse Sca-1 (Ly-6A/E) PE-Cy7 (clone E13-161.7)	BioLegend	Cat#: 122514
Anti-mouse Ter-119 BV510 (clone TER-119)	BD Biosciences	Cat#: 563995
Anti-mouse Ter-119 BV650 (clone TER-119)	BD Biosciences	Cat#: 747739
Anti-mouse Ter-119 BUV395 (clone TER-119)	BD Biosciences	Cat#: 563827
Anti-mouse Ter-119 eF450 (clone TER-119)	ThermoFisher Scientific	Cat#: 48-5921-82
Anti-mouse Thy1.2 BV605 (clone 53-2.1)	BioLegend	Cat#: 140318
Anti-mouse Streptavidin BV570	BioLegend	Cat#: 405227
Anti-mouse Streptavidin PE-TexasRed	BD Biosciences	Cat#: 551487
7-AAD	BD Biosciences	Cat#: 559925
DAPI	ThermoFisher Scientific	Cat#: D3571
Sytox Blue	ThermoFisher Scientific	Cat#: S34857
Ki-67 FITC	BD Biosciences	Cat#: 556026
BrdU PE	BD Biosciences	Cat#: 556029

Table S4. Immunophenotype of hematopoietic populations, related to Figures 1-7

**OP9 readout analysis**	
B cells	CD19^{+}B220^{+}
Myeloid cells	CD11b^{+}Gr-1^{+} and/or CD11b^{+}F4/80^{+}
**Peripheral blood analysis**	
B cells	CD41^{-} Ter119^{-} CD3e^{-} Thy1.2^{-} NK1.1^{-} CD49b^{-} CD11b^{-} Gr-1^{-} **B220^{+} CD19^{+}**
Myeloid cells	CD41^{-} Ter119^{-} CD3e^{-} Thy1.2^{-} NK1.1^{-} CD49b^{-} B220^{-} CD19^{-} **CD11b^{+}**
T cells	CD41^{-} Ter119^{-} NK1.1^{-} CD49b^{-} CD11b^{-} Gr-1^{-} B220^{-} CD19^{-} **CD3e^{+} Thy1.2^{+}**
NK cells	CD41^{-} Ter119^{-} B220^{-} CD19^{-} CD11b^{-} Gr-1^{-} CD3e^{-} Thy1.2^{-} **NK1.1^{+} CD49b^{+}**
Platelets	Ter119^{-} **CD41^{+} CD150^{+}**
Erythrocytes	CD41^{-} CD150^{-} **Ter-119^{+}**
**Bone marrow stem- and progenitor analysis**	
Lymphoid-primed multipotent progenitor (LMPP)	Lin^{-} Sca-1^{+} c-kit^{+} (LSK) Flt3^{hi}
Common lymphoid progenitor (CLP)	Lin^{-} B220^{low} Sca-1^{low} c-kit^{low} Flt-3^{hi} IL-7Ra^{+}
Megakaryocyte progenitor (MkP)	Lin^{-} Sca-1^{-} c-kit^{+} (LK) CD150^{+} CD41^{+}
Granulocyte-monocyte progenitor (GMP)	LK CD41^{-} CD150^{-} CD16/32^{+}
Pre-granulocyte-monocyte (Pre-GM)	LK CD41^{-} CD16/32^{-} CD150^{-} CD105^{-}
Pre-megakaryocyte-erythrocyte (Pre-MegE)	LK CD41^{-} CD16/32^{-} CD150^{+} CD105^{-}
Pre-colony forming unit erythroid (Pre-CFU-E)	LK CD41^{-} CD16/32^{-} CD150^{+} CD105^{+}
Colony forming unit erythroid (CFU-E)	LK CD41^{-} CD16/32^{-} CD150^{-} CD105^{+}
Hematopoietic stem cell (HSC)	LSK Flt-3^{-} CD48^{-} CD150^{+} or LSK CD48^{-} CD150^{+}
**Hematopoietic stem cell analysis**	
CD49b^{-} subset	LSK CD48$^{-/low}$ CD34$^{-/low}$ CD150^{hi} CD49b^{-}
CD49b^{+} subset	LSK CD48$^{-/low}$ CD34$^{-/low}$ CD150^{hi} CD49b^{+}
CD150^{hi} subset	LSK CD48$^{-/low}$ CD34$^{-/low}$ CD150^{hi}
CD150^{int} subset	LSK CD48$^{-/low}$ CD34$^{-/low}$ CD150^{int}
CD150^{-} subset	LSK CD48$^{-/low}$ CD34$^{-/low}$ CD150^{-}

Table S5. Cell culture conditions for in vitro assays, related to Figures 1-2

**Megakaryocyte liquid culture assay**	**Company**	**Catalogue number**	**Final concentration**
x-vivo15 with Gentamicin and L-glutamine	Lonza	BE02-060F	
BIT 9500	Stem Cell Technologies	09500	20% v/v
Fetal bovine serum (FBS)	Cytiva Hyclone	SH30071	10% v/v
β-mercaptoethanol	Merck	M7154-100ML	0.1mM
rmSCF	R&D Systems	455-MC	25 ng/ml
rmFlt3-L	Peprotech	250-31L	25 ng/ml
rmTpo	R&D Systems	488-TO	25 ng/ml
rmIL-3	R&D Systems	403-ML	10 ng/ml
**Cell division assay**	**Company**	**Catalogue number**	**Final concentration**
x-vivo15 with Gentamicin and L-glutamine	Lonza	BE02-060F	
Fetal bovine serum (FBS)	Cytiva Hyclone	SH30071	10% v/v
β-mercaptoethanol	Merck	M7154-100ML	0.1mM
rmSCF	Peprotech	250-03	50 ng/ml
rhFlt3-L	Peprotech	300-19	50 ng/ml
rhTpo	Peprotech	300-18	50 ng/ml
rmIL-3	Peprotech	213-13	20 ng/ml
**OP9 co-culture assay**	**Company**	**Catalogue number**	**Final concentration**
Opti-MEM with GlutaMAX	Gibco	51985-026	
Fetal bovine serum (FBS)	Cytiva Hyclone	SH30071	10% v/v
β-mercaptoethanol	Merck	M7154-100ML	0.1mM
Penicillin-Streptomycin	Gibco	15140122	1% v/v
rmSCF	R&D Systems or Peprotech	455-MC or 250-03	25 ng/ml
rmFlt3-L or rhFlt3-L	Peprotech	250-31L or 300-19	25 ng/ml
rmIL-7 or rhIL-7	R&D Systems or Peprotech	407-ML or 200-07	20 ng/ml

SUPPLEMENTAL EXPERIMENTAL PROCEDURES

Experimental animals

Animals were bred and maintained at the Preclinical Laboratory, Karolinska University Hospital and all experiments were approved by the regional ethical committee, Linköping ethical committee (ethical number 882). Females and males between 8-17 weeks old were used and were on a C57BL/6J background. B6.SJL-*Ptprc*^{a}*Pepc*^{b}/BoyCrl and B6.SJL-*Ptprc*^{a}*Pepc*^{b}/BoyJ mice (CD45.1) were used as primary and secondary recipients in transplantation experiments. Gata-1 eGFP[1] mice (CD45.2) were backcrossed >8 generations to a C57BL/6J background and were used as donor mice in transplantation experiments.

Hematopoietic cell preparation and staining

Bone marrow (BM) cell suspensions were prepared by crushing forelimbs, hindlimbs and hip bones into Phosphate-Buffered Saline (PBS, Gibco) with 5% FCS (Gibco) and 2 mM Ethylenediaminetetraacetic acid (EDTA, Merck).

Unfractionated BM cells were either Fc-blocked with purified CD16/32 (BD Biosciences) or pre-stained with fluorophore-conjugated CD16/32 antibody and subsequently stained with antibodies against cell surface marker antigens (Table S3). For detection of hematopoietic stem cells (HSCs), unfractionated BM cells were enriched using CD117 MicroBeads (Miltenyi Biotec) followed by immunomagnetic separation of the cells and subsequently stained with antibodies against cell-surface markers. Immunophenotype definitions of hematopoietic populations are described in Table S4.

Peripheral blood (PB) was collected from the tail vein into lithium heparin coated microvette tubes (Sarstedt) followed by platelet and erythrocyte isolation as performed in Carrelha *et al*[2]. Leukocytes were subsequently isolated from PB samples and stained with antibodies against cell surface antigens as described previously[3].

Flow cytometry experiments

Cell sorting experiments were performed using FACSAria™ Fusion or BD FACSAria™ III cell sorters (BD Biosciences) with a mean cell sorting purity of 95.3%±2.3%. Flow cytometry analysis was performed on LSR Fortessa™ or FACSymphony™ A5. Fluorescence minus one (FMO) controls were included in all flow cytometry experiments. Gates were set using FMO controls, backgating of the populations of interest or using internal controls (known negative and positive populations for the markers). Post-acquisition data analyses were done using the FlowJo software version 10 (BD Biosciences).

In single cell transplantation and single cell *in vitro* differentiation experiments, verification of single cell deposition into 96-well or 60-well plates was performed by sorting fluorescent beads.

Transplantation experiments

In all transplantation experiments, single cells or 5 cells were intravenously injected into lethally irradiated CD45.1 mice. The full irradiation dose was given as two split doses (2 x 600cGy). Donor and recipient mice were sex matched. Single cell transplantation experiments were performed as described in Carrelha *et al*[2]. In primary transplantations, single or five HSCs were transplanted with 200,000-250,000 BM support cells (CD45.1). Secondary transplantations were performed with reconstituted primary recipient mice using 10×10^{6} unfractionated BM or 30-100 sorted CD49b^{-} or CD49b^{+} HSCs with 200,000 BM support cells. Cells from one primary donor mouse were transplanted into 1-5 lethally irradiated secondary recipients. Peripheral blood analyses from transplanted mice were periodically performed between 2- and 6-months post-transplantation and up to 9 months in extended long-term experiments.

Reconstitution threshold levels and lineage bias

The total donor reconstitution level was calculated based on the frequency of CD45.2^{+} events in total white blood cells (CD45.1 + CD45.2 cells). The transplanted mice were considered reconstituted when the total donor contribution in white blood cells (CD45.2^{+}) or in platelets (Gata-1 eGFP^{+}) in the peripheral blood was $\geq$0.1% and represented by $\geq$10 events in the donor gate. The single cell transplantation efficiency was calculated based on the number of reconstituted mice at 2 months post-transplantation and the total number of mice that was transplanted. Mice that were found dead or sacrificed due to animal welfare reasons before 2 months post-transplantation were excluded from the total number of mice that was transplanted.

The reconstitution of B, T, NK and myeloid cells was calculated based on the frequency of CD45.2^{+} events within each blood cell lineage. The reconstitution of platelets and erythrocytes was calculated based

on the frequency of eGFP$^+$ cells within the blood lineages (Figure S2). Mice were considered positive for specific blood cell lineages when donor lineage reconstitution was ≥0.01% and represented by ≥10 events in the donor gate. Long-term (LT; ≥5-6 months) or short-term (ST; <5-6 months) repopulating activity was based on the presence or absence of myeloid or platelet and erythrocyte repopulation in the peripheral blood (≥0.01% CD45.2$^+$ CD11b$^+$ or eGFP$^+$CD41$^+$CD150$^+$ and eGFP$^+$Ter-119$^+$), 5-6 months following transplantation.

Relative donor reconstitution levels were calculated based on the frequency of B, T, NK and myeloid cells within CD45.2$^+$ cells. Lineage bias from single cell transplanted mice was categorized according to the ratio of lymphoid (L; including B, T and NK cells) to myeloid (M) cell contribution (L/M) in the peripheral blood at 5-6 months post-transplantation. A lineage-balanced pattern has a L/M ratio of 6.0±2.0[4]. Lineage-bias was therefore classified as myeloid-biased (L/M <4), lymphoid-biased (L/M >8) or lineage-balanced (L/M ≥4 and ≥8).

In HSC repopulation analyses on average 2 million events were recorded per sample and ≥10 events were used to determine whether mice were positively reconstituted for HSCs (Figures S2C and S5B).

In vitro assays

To assess myeloid and B cell lineage potentials of HSC subsets *in vitro,* OP9 co-culture experiments were performed with the OP9 cell line as described previously[5], and assessed after 3 weeks of culture.

Megakaryocyte and erythrocyte potentials of HSC subsets were evaluated as previously described[5]. Briefly, megakaryocyte potential was evaluated by manually plating 1 cell/well into 60-well MicroWell MiniTrays (Nunc, ThermoScientific) and assessed after 13 days of culture by an inverted microscope for the presence or absence of megakaryocytic cells in the cultures. Erythroid potential was evaluated by plating 30 HSCs in complete methylcellulose (GF M3434; StemCell Technologies). Cultures were evaluated for erythroid colonies after 12 days with 2,7-diaminofluorene staining (Merck) as previously described[5].

Cell division kinetics of single cell sorted HSC subsets were performed as previously described[3]. Briefly, single cells were sorted directly into 60-well MicroWell MiniTrays. The number of cells and their cell divisions in the wells were regularly scored using an inverted microscope at 24-, 48-, 72- and 96-hours post-sorting.

Cell culture conditions for different *in vitro* assays are described in Table S5.

Cell cycle and cell proliferation assays

Cell cycle analysis by Ki-67 staining was performed with the BD Cytofix/Cytoperm Kit (BD Biosciences). Cell proliferation analysis was performed by the 5-Bromo-2'-deoxyuridine (BrdU) incorporation assay[3] with one dose of intraperitoneal injection of BrdU (50ug/g bodyweight), followed by administration of BrdU (Merck) in the drinking water (800ug/ml) for three days following the intraperitoneal injection. BrdU visualization was performed with the BD BrdU Flow Kit (BD Biosciences) according to the manufacturer's instructions.

RNA-sequencing

For RNA-sequencing, 250 or 500 cells from CD45.1 mice were FACS-sorted into 5 or 10 ul of Single cell lysis solution containing DNAse I (Single cell lysis kit, Invitrogen/ThermoFisher Scientific) and after 15 minutes incubation the reaction was stopped by adding stop solution according to the manufacturer's protocol. cDNA synthesis for strand specific RNA-sequencing libraries was done on RNA from 250-500 cells using NEBNext Ultra II RNA First Strand Synthesis (New England BioLabs) and NEBNext Ultra II Directional RNA Second Strand Synthesis (New England BioLabs) modules, in combination with QIAseq FastSelect rRNA HMR kit (Qiagen) for rRNA block. Custom made Tn5 (transposase) and replacement index primers were used for library preparation. Libraries were pooled and paired-end sequenced (2 x 41 cycles) using the NextSeq 500 system (Illumina, San Diego, CA). Paired-end reads were mapped to the mm10 reference genome using STAR (v.2.5.2b)[6]. Data from technical replicates, derived from the same biological sample, were merged when available. Quantification of reads in exons was done using HOMER[7]. Data was normalized for sequencing depth by converting to transcripts per million (TPM) in R. Genes with ≥1 TPM in ≥3 samples were considered expressed and used for analysis. For PCA analysis and visualization of gene expression in heatmaps and boxplots $\log_2$(TPM+1) values were used. Correlation between samples was calculated using $\log_{10}$ transformed and quantile normalized data. Differential

expression analysis was done using edgeR[8], basing statistical significance on the absolute fold change and Benjamini-Hochberg adjusted p-value (p_{adj}) from edgeR's exact test.

Single-cell cDNA libraries were prepared according to the previously described Smart Seq2 protocol[9]. In brief, mRNA was transcribed into cDNA using oligo(dT) primer and SuperScript II reverse transcriptase (ThermoFisher Scientific). Second strand cDNA was synthetized using a template switching oligo. The synthetized cDNA was then amplified by polymerase chain reaction (PCR) for 25–26 cycles. Purified cDNA was quality controlled (QC) by fragment analysis on a 2100 Bioanalyzer with a DNA High Sensitivity chip (Agilent Biotechnologies). When the sample passed the QC, the cDNA was fragmented and tagged (tagmented) using Tn5 transposase, and each single well was uniquely indexed using the Illumina Nextera XT index kits (Set A–D). Thereafter, the uniquely indexed cDNA libraries from one 384-well plate were pooled into one sample to be sequenced on one lane of a HiSeq3000 sequencer (Illumina), using dual indexing and single 50 base-pair reads. Reads were demultiplexed, aligned to the mm10 reference genome using Tophat2 and deduplicated using samtools. Subsequent analysis was done in R using the Seurat package (v.4.1.0). Cells with an RNA count < 50,000 or >750,000 reads, with >10% mitochondrial reads, or with >10% contribution from the ERCC spike-in controls were excluded from analysis. Genes were filtered to have a read count >150 across all included cells. Data was then normalized using Seurat´s LogNormalize method with a scale factor of 500,000. PCA was run on the 2,000 most variable genes and the top 10 principal components were used as input to create UMAP plots. The likelihood-ratio test was used for differential analysis.

ATAC-sequencing

ATAC-seq was performed using the Omni-ATAC protocol with modifications[10]. Five hundred cells from CD45.1 mice were FACS sorted into tubes containing 12.5ul 2x TD buffer (Illumina), 5.75ul PBS and 2.5ul of water. After sorting 0.25µl each of digitonin and TWEEN 20 (Merck) was added, followed by 1.25ul of Tn5 transposase (Illumina). Samples were then incubated for 30 minutes at 37°C with agitation at 300 rpm and purified using a MinElute PCR Purification Kit (Qiagen). Library fragments were amplified using 1xNEBnext PCR master mix (New England BioLabs) and 0.1 µM of custom PCR primers. Libraries were pooled and paired-end sequenced (2 x 41 cycles) using the NextSeq 550 system (Illumina, San Diego, CA). The nf-core ATAC-sequencing pipeline (v.1.0.0)[11] was used to align reads to the mm10 reference genome, filter aligned reads and for quality control. Data from technical replicates, derived from the same biological sample, was merged when available. For visualization, read coverage was normalized to 10^6 mapped reads in peaks and tracks with the median read coverage ± standard deviation was created for each population to allow for visualization using the UCSC genome browser[12]. Peak calling, quantification of reads in consensus peaks and peak annotation were done using HOMER[7], with options "-region -size 100 -minDist 200" for peak calling. Read counts were $\log_{10}$ transformed and quantile normalized in R. Peaks were considered to be found in a population if they had a normalized read count >1.5 in more than one third of the samples. Peaks were filtered to exclude those not found in any analyzed population. For specific analysis of the $CD150^+$ populations, peaks not found in any $CD150^+$ population were additionally excluded. Log_{10} transformed and quantile normalized data was used for PCA analysis, visualization of accessibility in heatmaps and calculation of correlation between samples. DESeq2[13] was used for differential analysis on the filtered peak sets, basing statistical significance on the absolute fold change and Benjamini-Hochberg adjusted p-value (p_{adj}) from the nbinomWaldTest. Enriched binding motifs in peaks with increased accessibility were identified with the HOMER program findMotifGenome.pl using a custom background of peaks with decreased accessibility in the respective population. Motifs found in less than 10% of peaks were excluded. Gene ontology analysis GREAT[14] (v.4.0.4) was used with online default settings. For footprinting analysis, the aligned and filtered samples of each population were down sampled to the same depth and merged, and the TOBIAS framework[15] was used for all further analysis with default settings and the JASPAR 2020 CORE vertebrate collection as TF motif database[16].

Statistical methods

Statistical analysis not explicitly described above was done in GraphPad Prism v.9.2.0 for Mac OS. Non-parametric tests were performed using the Mann-Whitney test or the Kruskal-Wallis with Dunn's multiple comparison test. Generally, at least 3 biological replicates were included in each test group, and the mean ± SD are shown. A biological replicate constitutes cells from one mouse or from a pool of mice. Statistically significant differences are indicated in all figures with exact P-values, unless stated otherwise.

Supplemental references

1. Drissen R, Buza-Vidas N, Woll P, Thongjuea S, Gambardella A, Giustacchini A, et al. Distinct myeloid progenitor differentiation pathways identified through single cell RNA sequencing. Nat Immunol. 2016 Jun;17(6):666–76.

2. Carrelha J, Meng Y, Kettyle LM, Luis TC, Norfo R, Alcolea V, et al. Hierarchically related lineage-restricted fates of multipotent haematopoietic stem cells. Nature. 2018 Feb 1;554(7690):106–11.

3. Luc S, Huang J, McEldoon JL, Somuncular E, Li D, Rhodes C, et al. Bcl11a Deficiency Leads to Hematopoietic Stem Cell Defects with an Aging-like Phenotype. Cell Reports. 2016 Sep 20;16(12):3181–94.

4. Müller-Sieburg CE, Cho RH, Thoman M, Adkins B, Sieburg HB. Deterministic regulation of hematopoietic stem cell self-renewal and differentiation. Blood. 2002 Aug 15;100(4):1302–9.

5. Luc S, Luis TC, Boukarabila H, Macaulay IC, Buza-Vidas N, Bouriez-Jones T, et al. The Earliest Thymic T Cell Progenitors Sustain B Cell and Myeloid Lineage Potentials. Nat Immunol. 2012 Apr;13(4):412–9.

6. Dobin A, Davis CA, Schlesinger F, Drenkow J, Zaleski C, Jha S, et al. STAR: ultrafast universal RNA-seq aligner. Bioinformatics. 2013;29(1):15–21.

7. Heinz S, Benner C, Spann N, Bertolino E, Lin YC, Laslo P, et al. Simple Combinations of Lineage-Determining Transcription Factors Prime cis-Regulatory Elements Required for Macrophage and B Cell Identities. Mol Cell. 2010;38(4):576–89.

8. Robinson MD, McCarthy DJ, Smyth GK. edgeR: a Bioconductor package for differential expression analysis of digital gene expression data. Bioinformatics. 2010;26(1):139–40.

9. Picelli S, Björklund ÅK, Faridani OR, Sagasser S, Winberg G, Sandberg R. Smart-seq2 for sensitive full-length transcriptome profiling in single cells. Nat Methods. 2013;10(11):1096–8.

10. Corces MR, Trevino AE, Hamilton EG, Greenside PG, Sinnott-Armstrong NA, Vesuna S, et al. An improved ATAC-seq protocol reduces background and enables interrogation of frozen tissues. Nat Methods. 2017 Aug 28;14(10):959–62.

11. Ewels PA, Peltzer A, Fillinger S, Patel H, Alneberg J, Wilm A, et al. The nf-core framework for community-curated bioinformatics pipelines. Nat Biotechnol. 2020;38(3):276–8.

12. Kent WJ, Sugnet CW, Furey TS, Roskin KM, Pringle TH, Zahler AM, et al. The Human Genome Browser at UCSC. Genome Res. 2002;12(6):996–1006.

13. Love MI, Huber W, Anders S. Moderated estimation of fold change and dispersion for RNA-seq data with DESeq2. Genome Biol. 2014;15(12):550.

14. McLean CY, Bristor D, Hiller M, Clarke SL, Schaar BT, Lowe CB, et al. GREAT improves functional interpretation of cis-regulatory regions. Nat Biotechnol. 2010;28(5):495–501.

15. Bentsen M, Goymann P, Schultheis H, Klee K, Petrova A, Wiegandt R, et al. ATAC-seq footprinting unravels kinetics of transcription factor binding during zygotic genome activation. Nat Commun. 2020;11(1):4267.

16. Fornes O, Castro-Mondragon JA, Khan A, Lee R van der, Zhang X, Richmond PA, et al. JASPAR 2020: update of the open-access database of transcription factor binding profiles. Nucleic Acids Res. 2020;48(D1):D87–92.
