# Supplementary material for: CD49b identifies functionally and epigenetically distinct subsets of lineage-biased hematopoietic stem cells
Source: Stem Cell Reports. 2022 Jun 16;17(7):1546–60. doi: 10.1016/j.stemcr.2022.05.014 (PMC9287668; doi:10.1016/j.stemcr.2022.05.014)
Supplement: Document S2. Article plus supplemental information [file mmc4.pdf]

Stem Cell Reports
Article
OPEN ACCESS

CD49b identifies functionally and epigenetically distinct subsets of lineage-biased hematopoietic stem cells

Ece Somuncular,[1,2] Julia Hauenstein,[1,3,7] Prajakta Khalkar,[1,2,7] Anne-Sofie Johansson,[1,2] Özge Dumral,[1,2] Nicolai S. Frengen,[1,3] Charlotte Gustafsson,[1,3] Giuseppe Mocci,[2,4] Tsu-Yi Su,[1,2] Hugo Brouwer,[1] Christine L. Trautmann,[1] Michael Vanlandewijck,[2,4,5] Stuart H. Orkin,[6] Robert Månsson,[1,3] and Sidinh Luc[1,2,*]

[1]Center for Hematology and Regenerative Medicine, Karolinska Institutet, Stockholm, Sweden
[2]Department of Medicine Huddinge, Karolinska Institutet, Stockholm, Sweden
[3]Department of Laboratory Medicine, Karolinska Institutet, Stockholm, Sweden
[4]Single Cell Core Facility of Flemingsberg Campus, Karolinska Institutet, Stockholm, Sweden
[5]Department of Immunology, Genetics and Pathology, Rudbeck Laboratory, Uppsala University, Uppsala, Sweden
[6]Dana-Farber/Boston Children's Cancer and Blood Disorders Center, Harvard Medical School, Howard Hughes Medical Institute, Boston, MA, USA
[7]These authors contributed equally
*Correspondence: Sidinh.Luc@ki.se
https://doi.org/10.1016/j.stemcr.2022.05.014

SUMMARY

Hematopoiesis is maintained by functionally diverse lineage-biased hematopoietic stem cells (HSCs). The functional significance of HSC heterogeneity and the regulatory mechanisms underlying lineage bias are not well understood. However, absolute purification of HSC subtypes with a pre-determined behavior remains challenging, highlighting the importance of continued efforts toward prospective isolation of homogeneous HSC subsets. In this study, we demonstrate that CD49b subdivides the most primitive HSC compartment into functionally distinct subtypes: CD49b$^-$ HSCs are highly enriched for myeloid-biased and the most durable cells, while CD49b$^+$ HSCs are enriched for multipotent cells with lymphoid bias and reduced self-renewal ability. We further demonstrate considerable transcriptional similarities between CD49b$^-$ and CD49b$^+$ HSCs but distinct differences in chromatin accessibility. Our studies highlight the diversity of HSC functional behaviors and provide insights into the molecular regulation of HSC heterogeneity through transcriptional and epigenetic mechanisms.

INTRODUCTION

The maintenance and replenishment of the hematopoietic system and its cells rely on rare bone marrow (BM)-resident hematopoietic stem cells (HSCs). Blood cell development is traditionally described as a hierarchical tree, with HSCs differentiating through progenitor stages to ultimately form the terminally differentiated hematopoietic cells (Wilkinson et al., 2020). In this model, HSCs are assumed to be multipotent and equipotent, thus producing all mature blood cells with no lineage preference (Eaves, 2015). However, HSCs exhibit distinct functional behaviors *in vivo*, with only a subset of them showing a lineage-balanced output consistent with traditionally described HSCs. The functionally heterogeneous HSCs have been classified according to the ratio of mature myeloid to lymphoid cells within the leukocyte fraction (Eaves, 2015). Later studies, which included the analysis of platelet and erythroid cells, led to the discovery of additional biased and restricted HSCs (Wilkinson et al., 2020). To date, HSCs categorized by myeloid-biased, platelet-biased, lineage-balanced, and lymphoid-biased repopulating patterns have been demonstrated (Carrelha et al., 2018; Challen et al., 2010; Dykstra et al., 2007; Morita et al., 2010; Müller-Sieburg et al., 2002; Muller-Sieburg et al., 2004; Sanjuan-Pla et al., 2013; Yamamoto et al., 2013, 2018). The behavior of distinct HSC subsets differs not only in lineage bias, but also in self-renewal ability (Eaves, 2015; Wilkinson et al., 2020). Myeloid-biased, platelet-biased, and lineage-balanced HSCs are recognized as durable HSCs that can sustain long-term (LT) hematopoiesis (Carrelha et al., 2018; Dykstra et al., 2007; Eaves, 2015; Morita et al., 2010; Sanjuan-Pla et al., 2013). Lymphoid-biased HSCs generally have finite self-renewal ability and likely overlap with intermediate-term (IT) HSCs and, thus, are not considered LT HSCs, but are still distinct from transiently self-renewing short-term (ST) HSCs and multipotent progenitors (MPPs) (Benveniste et al., 2010; Challen et al., 2010; Dykstra et al., 2007; Eaves, 2015; Kent et al., 2009; Morita et al., 2010). Altogether, these studies suggest that there is a large diversity of cells within the traditional HSC compartment, which exhibit lineage-bias differences and gradual differences in self-renewal potential and thereby durability.

Stem and progenitor cells are contained within the Lineage$^-$Sca-1$^+$c-Kit$^+$ (LSK) compartment, but only a minor part of LSK cells are LT reconstituting HSCs. The combination of additional cell-surface markers has greatly improved the isolation of functional HSCs. However, HSCs are ultimately defined by their functional ability and cannot be identified by immunophenotype alone (Wilkinson et al.,

This is an open access article under the CC BY license (http://creativecommons.org/licenses/by/4.0/).

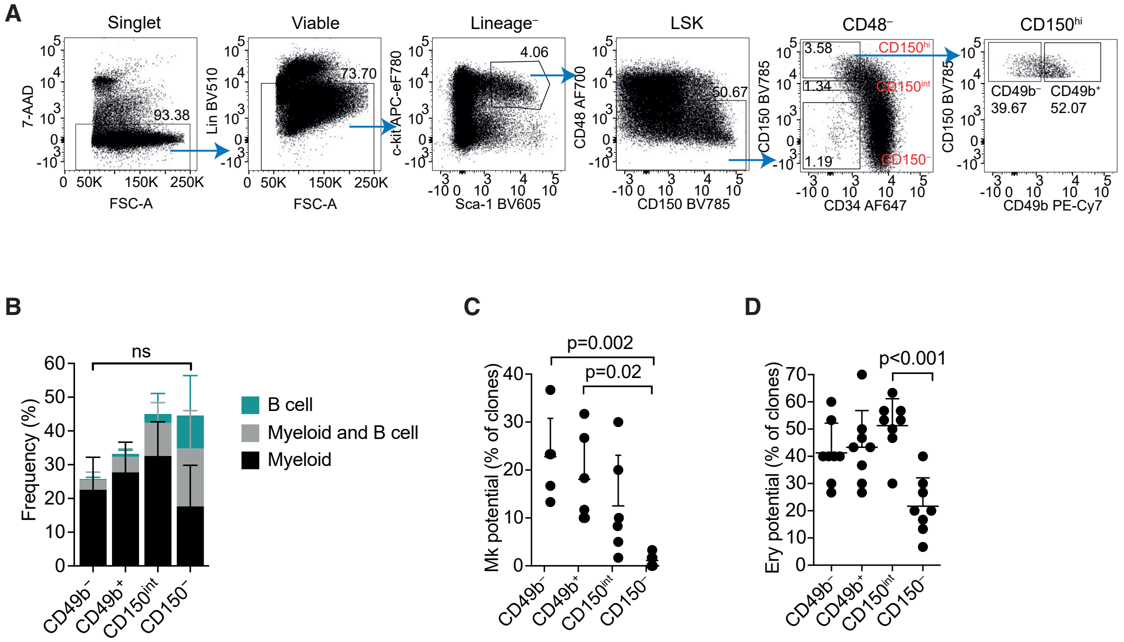

Figure 1. The HSC compartment can be further subfractionated with CD49b

(A) Fluorescence-activated cell sorting (FACS) profile and gating strategy of phenotypic HSC subsets and further separation with CD49b in CD117-enriched BM cells. Frequencies of parent gates are shown.

(B) *In vitro* differentiation potential of single sorted cells to myeloid (CD11b$^+$Gr-1$^+$ and/or CD11b$^+$F4/80$^+$) and B cells (B220$^+$CD19$^+$, n_{CD49b^-} = 568 cells, n_{CD49b^+} = 536 cells, $n_{CD150^{int}}$ = 401 cells, n_{CD150^-} = 409 cells; nine replicates; five independent experiments). ns, not significant.

(C) Megakaryocyte differentiation culture of single plated cells (n = 360 cells/population, six replicates, three independent experiments).

(D) Erythroid colony-forming assay of CD49b$^-$, CD49b$^+$, CD150^{int}, and CD150$^-$ cells (n = 8 replicates/population, 30 cells per replicate, two independent experiments). Mean ± SD is shown in (B–D). Statistical significance in (B) was calculated based on total cloning frequency. See also Figure S1.

2020). Lineage bias is, at least partly, thought to be intrinsically programmed. Further study of the mechanisms underlying HSC diversity is dependent upon the ability to connect HSC immunophenotype with functional behavior, highlighting the importance of prospective isolation of homogeneous HSC subsets (Eaves, 2015; Haas et al., 2018).

In this study, we have reassessed the phenotypic HSC compartment using cell-surface markers reported to identify HSCs, to explore whether different combinations of immunophenotypes can isolate functionally diverse HSC subsets. We found heterogeneous expression of CD49b in the phenotypic HSC fraction harboring the most primitive and durable cells (LSKCD34$^-$CD48$^-$CD150^{hi}) (Morita et al., 2010). Phenotypically separated CD49b fractions were functionally distinct: CD49b$^-$ cells were highly enriched for myeloid-biased HSCs and were the most durable cells, and CD49b$^+$ cells were enriched for multipotent HSCs with lymphoid bias and less durability. Transcriptional profiling of CD49b$^-$ and CD49b$^+$ HSCs revealed high concordance, whereas chromatin accessibility analysis showed diverse profiles. Our studies demonstrate that CD49b can distinguish between functionally and epigenetically distinct multipotent HSCs with myeloid and lymphoid bias in the primitive HSC compartment.

RESULTS

The HSC compartment can be further subfractionated with CD49b

We tested previously reported HSC markers to explore further subfractionation, including CD41 and CD244 (Kiel et al., 2005), Flt-3 (Adolfsson et al., 2001), Tie-2 (Arai et al., 2004), CD201 (EPCR; Balazs et al., 2006), CD61 (Mann et al., 2018), CD86 (Shimazu et al., 2012), CD9 (Karlsson et al., 2013), Esam (Sudo et al., 2012), CD229 (Oguro et al., 2013), and CD49b (Wagers and Weissman, 2006) (Figure S1A). Since CD150 expression is positively correlated with self-renewal, we focused primarily on the LSKCD34$^-$CD48$^-$CD150^{hi} (CD150^{hi}) fraction, enriched for myeloid-biased HSCs (Figure 1A). The LSKCD34$^-$CD48$^-$CD150^{int}

(CD150^{int}) cells, enriched for lineage-balanced HSCs, and the LSKCD34^{-}CD48^{-}CD150^{-} (CD150^{-}) cells, with a lymphoid-biased phenotype, were included for comparison (Figure 1A) (Kent et al., 2009; Morita et al., 2010). Most markers had uniform expression patterns in the CD150^{hi} fraction except for CD229, CD41, and CD49b, which showed bimodal expression profiles (Figure S1A). Interestingly, CD49b has been suggested to mark ST HSCs, IT HSCs, and primed HSCs (Benveniste et al., 2010; Wagers and Weissman, 2006; Zhao et al., 2019). We found that previously identified CD49b^{-} and CD49b^{+} populations were heterogeneous for CD150 cell-surface expression, which could partly explain why CD49b marks cells with both transient and LT self-renewal ability (Figures S1B and S1C). We therefore hypothesized that CD49b might be a candidate marker to further enhance the isolation of functionally distinct HSCs within the CD150^{hi} compartment. Notably, the combination of CD229, CD41, and CD49b revealed further phenotypic subfractions within CD150^{hi} cells (Figure S1D). However, cell-cycle analysis by Ki-67 staining and cell-proliferation analysis by the 5-bromo-2′-deoxyuridine (BrdU) incorporation assay showed significant differences only between the CD49b^{-} and the CD49b^{+} subsets (Figures S1E–S1H). These data suggested that subfractionation with CD49b alone could be sufficient to isolate functionally distinct cells.

We therefore isolated LSKCD34^{-}CD48^{-}CD150^{hi}CD49b^{-} (CD49b^{-}) and LSKCD34^{-}CD48^{-}CD150^{hi}CD49b^{+} (CD49b^{+}) cells (Figures 1A and S1I) to assess *in vitro* differentiation abilities (Figures 1B–1D). In combined B cell and myeloid cell (B/M) cultures, all investigated subsets produced B cells and myeloid cells. However, CD49b^{-} and CD49b^{+} subsets mainly generated myeloid cells, consistent with the CD150^{hi} fraction containing myeloid-biased cells. In contrast, CD150^{-} cells efficiently produced B cells, in line with enrichment of lymphoid-biased cells in the CD150^{-} fraction (Figure 1B) (Kent et al., 2009; Morita et al., 2010). Furthermore, CD49b^{-} and CD49b^{+} subsets efficiently differentiated into megakaryocytes and erythroid cells, whereas the CD150^{-} fraction was the least efficient (Figures 1C and 1D). Collectively, these results suggested that CD49b^{-} and CD49b^{+} cells have similar *in vitro* differentiation abilities.

CD49b^{-} and CD49b^{+} subsets have different cell-cycle and cell-proliferation kinetics

Despite the lack of functional differences between CD49b^{-} and CD49b^{+} cells *in vitro*, we observed a higher, but not statistically significant, cloning frequency from the CD49b^{+} subset in the B/M assay, compatible with cell-proliferation differences (Figures 1B and S1H). Cell-cycle analysis of CD49b^{-} and CD49b^{+} cells showed that both subsets were highly quiescent, but CD49b^{-} cells were more in G0 (95%) and less in G1 (4%) compared with CD49b^{+} cells (87% and 10%, respectively; Figures 2A and 2B). Consistent with the higher *in vitro* cloning frequency (Figure 1B), more CD49b^{+} cells (30%) incorporated BrdU compared with CD49b^{-} cells (11%) (Figures 2C and 2D). To further understand cell-proliferation kinetics, we tracked the cell divisions of CD49b^{-} and CD49b^{+} single cells (Figure 2E). One day after culture, most cells had still not divided (CD49b^{-}, 96%; CD49b^{+}, 89%), consistent with the quiescent nature of both subsets (Figure 2B). However, after 2 days, 60% of CD49b^{+} cells had already undergone ≥ 1 cell division, compared with only 36% of CD49b^{-} cells, indicating that CD49b^{+} cells entered the cell cycle faster. By 4 days, the numbers of cell divisions were comparable between the subsets.

Collectively, these findings suggested that, although both CD49b^{-} and CD49b^{+} subsets were highly quiescent, CD49b^{+} cells had a higher proliferation rate.

CD49b^{-} and CD49b^{+} HSCs are the most durable subsets

To evaluate the functional significance of the CD49b subfractions, we performed a competitive transplantation assay, in which five cells of the CD49b^{-}, CD49b^{+}, CD150^{int}, or CD150^{-} subsets were transplanted. Donor HSC subsets were purified from the *Gata-1* eGFP mouse strain to detect platelets and erythrocytes (Figure S2A) (Drissen et al., 2016). Although the transplanted HSC subsets differed in total leukocyte contribution, they repopulated all blood cells (Figures 3A, 3B, and S2B). While the CD49b^{-}, CD49b^{+}, and CD150^{int} subsets could stably generate all blood lineages for 6 months, CD150^{-} transplanted mice exhibited transient myeloid repopulation but sustained high B and T cell repopulation over time, consistent with previous studies (Kent et al., 2009; Morita et al., 2010). Furthermore, donor-derived phenotypic HSCs were detected in most mice from the CD49b^{-} (92%) and CD49b^{+} (90%) groups, in 42% of mice from CD150^{int}, but in only 7% of mice from CD150^{-} (Figures 3C and S2C). Mice from the CD49b^{-} and CD49b^{+} groups generated all types of mature BM cells, progenitor cells, and phenotypic HSCs at 5–6 months post-transplantation (Figures 3C and S2D–S2F). Collectively, these findings indicated that CD49b^{-} and CD49b^{+} subsets had the highest self-renewal potential.

CD49b^{-} and CD49b^{+} HSCs reconstitute all blood lineages, but at different ratios

To evaluate the HSC behavior at the clonal level, we performed single-cell transplantation experiments. A total of 139 mice were transplanted with single CD49b^{-} (61 mice) or CD49b^{+} (78 mice) cells, and 48% of the CD49b^{-} and 28% of the CD49b^{+} cells reconstituted the

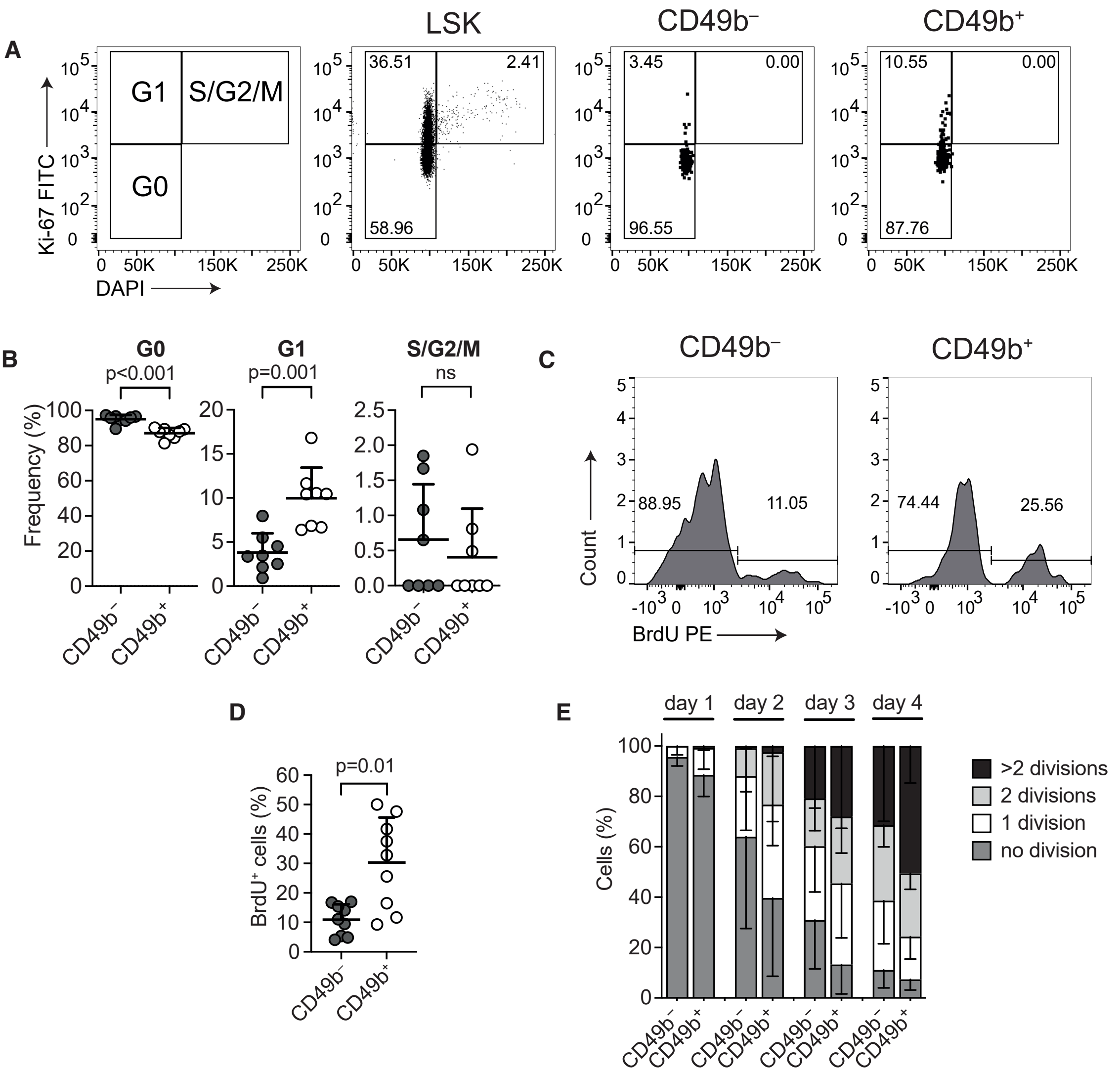

Figure 2. CD49b^{-} and CD49b^{+} subsets have different cell-cycle and cell-proliferation kinetics

(A) Cell-cycle analysis of CD117-enriched BM cells. The frequency of G0, G1, and S/G2/M cells in a representative mouse is shown.
(B) Frequency of CD49b^{-} and CD49b^{+} HSCs in G0, G1, and S/G2/M (n = 8 mice, two independent experiments). ns, not significant.
(C) BrdU analysis of CD117-enriched BM cells. The frequency of BrdU^{-} and BrdU^{+} cells in a representative mouse is shown.
(D) Frequency of BrdU^{+} CD49b^{-} and CD49b^{+} HSCs (n = 9 mice, three independent experiments).
(E) Cell divisions from cultured single cells on days 1–4 ($n_{CD49b^{-}}$ = 347 cells, $n_{CD49b^{+}}$ = 370 cells, five replicates, three independent experiments). Mean ± SD is shown in (B), (D), and (E).

recipients (Figure S3). Both subsets were able to repopulate all leukocyte, platelet, and erythrocyte lineages for 6 months (Figures 4A and 4B). The repopulation profiles of individual mice revealed five major patterns from CD49b^{-} cells where the dominant groups were characterized by high platelet (P), erythroid (E), and myeloid contributions (M > L) and multilineage contributions (M-L) (Figures S3 and S4A). Notably, mice with only P or PE repopulation patterns were found only in CD49b^{-} cells (Figures S3E, S3F, and S4A). Conversely, CD49b^{+} cells could be grouped into four patterns with predominantly higher B lymphoid reconstitution (L > M) or ST/transient repopulation patterns (Figure S4B). Thus, we assessed the lineage bias by analyzing the proportional contribution to B, T, natural killer (NK), and myeloid cells in donor leukocytes (Figures 4C and 4D). Single-cell-transplanted CD49b^{-} mice showed a high myeloid contribution, and CD49b^{+} mice exhibited predominantly lymphoid contribution and some lymphoid-restricted patterns. Based on blood profiles from unmanipulated mice, a lineage-balanced

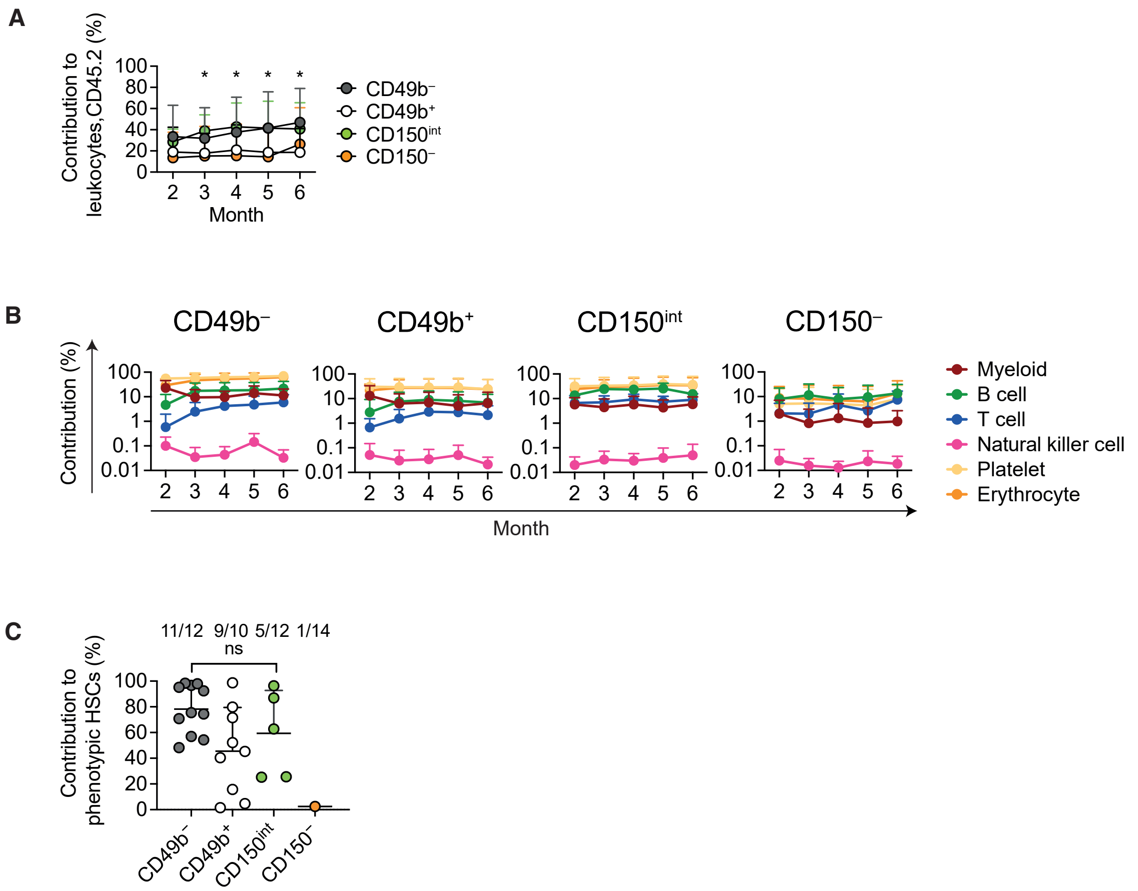

Figure 3. CD49b$^-$ and CD49b$^+$ HSCs are the most durable subsets

(A and B) Total donor contribution (A) and donor contribution to platelets, erythrocytes, and myeloid, B, T, and natural killer cells (B) in peripheral blood (PB) from five cell transplantations (n_{CD49b^-} = 28 mice, n_{CD49b^+} = 22 mice, $n_{CD150^{int}}$ = 12 mice, n_{CD150^-} = 13 mice, three independent experiments).

(C) Donor contribution to phenotypic HSCs (LSKFlt-3$^-$CD48$^-$CD150$^+$ or LSKCD48$^-$CD150$^+$), 5–6 months after five cell transplantations (n_{CD49b^-} = 12 mice, n_{CD49b^+} = 10 mice, $n_{CD150^{int}}$ = 12 mice, n_{CD150^-} = 13 mice, three independent experiments). The numbers of reconstituted mice out of all mice analyzed are indicated. ns, not significant. Asterisks indicate statistically significant differences: $p = 0.028$, CD49b$^-$ versus CD150$^-$, and $p = 0.05$, CD150^{int} versus CD150$^-$, month 2; $p = 0.032$, CD49b$^-$ versus CD150$^-$, and $p = 0.006$, CD150^{int} versus CD150$^-$, month 3; $p = 0.015$, CD49b$^-$ versus CD150$^-$, and $p = 0.005$, CD150^{int} versus CD150$^-$, month 4; $p = 0.005$, CD49b$^-$ versus CD150$^-$, and $p = 0.013$, CD150^{int} versus CD150$^-$, month 5; and $p = 0.016$, CD49b$^-$ versus CD49b$^+$, month 6. Mean ± SD is shown in (A–C). See also Figure S2.

pattern can be categorized by the ratio of lymphoid to myeloid cell contribution (L/M) of 6.0 ± 2.0 (Figure S4C) (Müller-Sieburg et al., 2002). Thus, we categorized the repopulation patterns from the CD49b$^-$ and CD49b$^+$ single-cell-transplanted mice as myeloid-biased (L/M < 4), lymphoid-biased (L/M > 8), or lineage-balanced (L/M ≥ 4 and ≤ 8). CD49b$^-$-transplanted mice were predominantly classified as myeloid-biased (M-bi; 78%), whereas the most common classification in CD49b$^+$-transplanted mice was lymphoid-biased (L-bi; 46%, Figure 4E). Furthermore, CD49b$^-$ generated more myeloid cells in the BM compared with CD49b$^+$, consistent with the M-bi repopulation pattern in peripheral blood (PB), while the lymphoid cell contributions in CD49b$^-$ and CD49b$^+$ cells were comparable (Figure S4D). These findings suggested that both CD49b$^-$ and CD49b$^+$ subsets were multipotent but with different lineage biases.

CD49b$^-$ HSCs are the most durable stem cells

An L-bi repopulation pattern is generally associated with declining myeloid repopulation and limited self-renewal ability due to a lack of LT HSC activity (Kent et al., 2009; Morita et al., 2010). We therefore assessed the LT (≥5–6 months) and ST (<5–6 months) repopulating ability of CD49b$^-$ and CD49b$^+$ subsets based on the presence of myeloid cells or platelets and erythrocytes in PB.

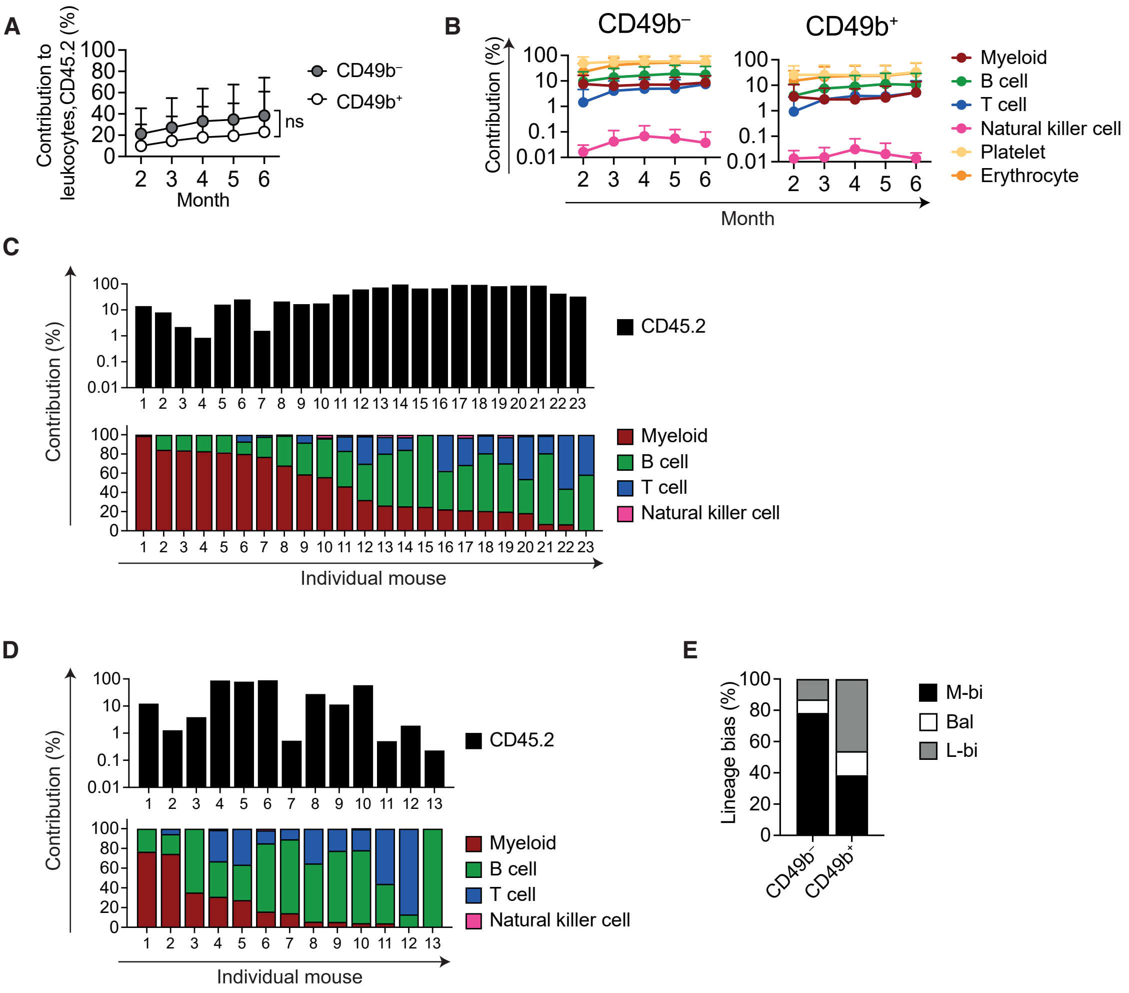

Figure 4. CD49b$^-$ and CD49b$^+$ HSCs reconstitute all blood lineages, but at different ratios

(A and B) Total donor contribution (A) and donor contribution to platelets, erythrocytes, and myeloid, B, T, and natural killer cells (B) in PB from single-cell transplantation (n_{CD49b^-} = 28 mice, n_{CD49b^+} = 18 mice, five independent experiments). ns, not significant.

(C and D) Total donor contribution (top) and relative contribution to myeloid, B, T, and natural killer cells (bottom) in PB of CD49b$^-$ (C) or CD49b$^+$ (D) single-cell-transplanted mice, 5–6 months post-transplantation (n_{CD49b^-} = 23 mice, n_{CD49b^+} = 13 mice, five independent experiments).

(E) Lineage bias categorization of single-cell-transplanted mice 5–6 months post-transplantation (n_{CD49b^-} = 23 mice, n_{CD49b^+} = 13 mice, five independent experiments). M-bi, myeloid-biased; Bal, balanced; L-bi, lymphoid-biased. Mean ± SD is shown (A and B). See also Figures S3–S5.

Eighty-nine percent of reconstituted mice transplanted with single CD49b$^-$ cells exhibited LT repopulating ability, of which 82% were M-bi. In contrast, 61% of CD49b$^+$-reconstituted mice showed LT activity, of which 36% were L-bi (Figures 5A and 5B). As expected, no CD49b$^+$ cells with lymphoid-restricted patterns and LT activity were found (Figures 4D and 5A). Furthermore, both CD49b$^-$ and CD49b$^+$ subsets generated phenotypic HSCs and downstream BM progenitor populations, but less efficiently in CD49b$^+$ compared with CD49b$^-$ cells (Figures 5C, S4E, and S4F). These data showed that CD49b$^-$ cells were more durable than CD49b$^+$ cells.

To assess whether LT repopulating activity decreased over time, we followed a group of single-cell-transplanted mice to 9 months. Both the repopulation level and the pattern were similar to the results at 5–6 months post-transplantation (Figures 4B–4D, 5D, and 5E), suggesting no decline of self-renewal potential in CD49b$^-$ and CD49b$^+$ subsets over time.

To conclusively compare the self-renewal ability of CD49b$^-$ and CD49b$^+$ cells, mice with phenotypic HSC repopulation were secondarily transplanted. Overall, the repopulation pattern in PB was preserved from primary to secondary recipients (Figures 5F and S5A). The repopulation

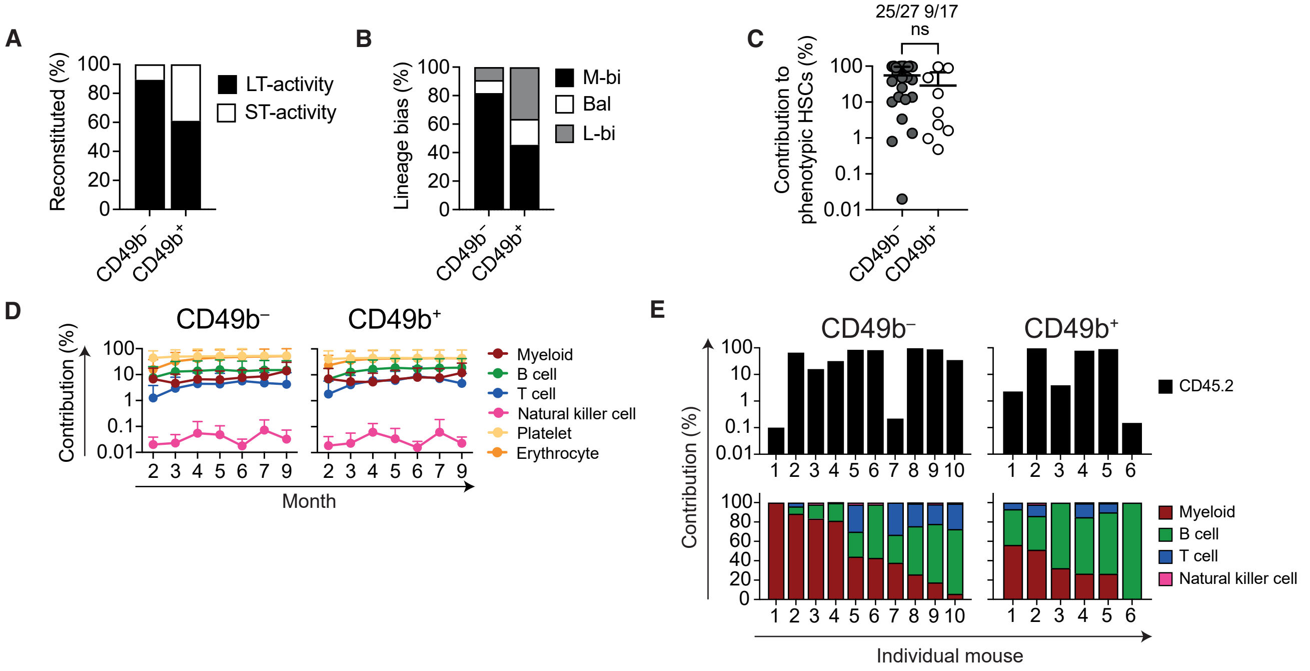

F

	1° transplantation (5-12 months)			2° transplantation (5-6 months)			
	Lin bias 1° mice	LT PB (M$^+$ or P$^+$ and Ery$^+$)	Phenotypic HSCs	Transplanted into 2°	LT PB (M$^+$ or P$^+$ and Ery$^+$)	Lin bias 2° mice	Phenotypic HSCs
CD49b$^-$	M-bi	19	19	17	17	14 M-bi<br>2 Bal<br>1 L-bi	13<br>2<br>1
	Bal	2	2	2	2	2 L-bi	2
	L-bi	2	2	2	0	0	0
	PE-only	2	2	–	–	–	–
	Total	***25***	***25***	***21***	***19***		***18***
CD49b$^+$	M-bi	4	4	4	4	1 M-bi<br>1 M-bi + 1 Bal<br>1 L-bi	1<br>1<br>1
	Bal	2	1	1	1	1 M-bi + 1 Bal	1
	L-bi	4	4	2	1	1 Bal	1
	Total	***10***	***9***	***7***	***6***		***5***
Number of primary transplanted mice is indicated for each section							

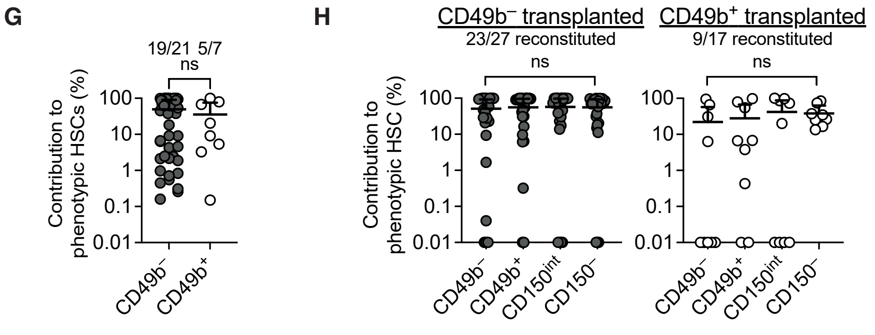

Figure 5. CD49b$^-$ HSCs are the most durable stem cells

(A) Short-term (ST) or long-term (LT) HSC activity of reconstituted single-cell-transplanted mice (n_{CD49b^-} = 28 mice, n_{CD49b^+} = 18 mice, five independent experiments). LT defined as myeloid (M)$^+$ or platelet (P)$^+$ and erythrocyte (Ery)$^+$ in PB, 5–6 months post-transplantation.

(B) Lineage bias distribution of single-cell-transplanted mice with LT activity in (A) (n_{CD49b^-} = 25 mice, n_{CD49b^+} = 11 mice, five independent experiments).

(C) Donor contribution to phenotypic HSCs (LSKFlt-3$^-$CD48$^-$CD150$^+$ or LSKCD48$^-$CD150$^+$) 5–12 months after single-cell transplantation (n_{CD49b^-} = 27 mice, n_{CD49b^+} = 17 mice, five independent experiments). ns, not significant.

(legend continued on next page)

efficiency of CD49b$^-$-transplanted mice was high, with all reconstituted mice with LT activity (Figure 5A) showing LT repopulation in PB and BM (25/25). Most of these mice further repopulated secondary recipients in PB and BM (18/21). In contrast, the repopulation efficiency of CD49b$^+$ cells in both primary (9/10) and secondary (5/7) transplantation was lower, but they were still capable of multilineage repopulation (Figures 5F, 5G, S5A, and S5B).

An M-bi classification was highly correlated with positive and robust repopulation. In contrast, an L-bi classification was correlated with declining repopulation efficiency from primary to secondary transplantation. Although these findings indicated that L-bi cells were capable of propagating through serial transplantation with multilineage repopulation (Figures 5F, 5G, S5A, and S5B), our results nonetheless suggested that such cells were infrequent.

Given the difference in durability and lineage bias, we evaluated the hierarchical relationship between the CD49b subsets by assessing their ability to form phenotypic HSC subsets (CD49b$^-$, CD49b$^+$, CD150^{int}, and CD150$^-$). While CD49b$^-$ cells were potent in generating all phenotypically defined HSC subsets, CD49b$^+$ cells were less efficient, but nevertheless able to produce the same subsets (Figures 5H and S5C). Secondary transplantation confirmed that most of the phenotypic donor-derived CD49b$^-$ and CD49b$^+$ cells were functional HSCs (Figures S5D and S5E).

Collectively, these results showed that both CD49b$^-$ and CD49b$^+$ cells could sustain LT multilineage hematopoiesis with preserved lineage bias but at different efficiencies.

The CD49b$^-$ and CD49b$^+$ subsets are transcriptionally similar but epigenetically distinct

To assess the molecular basis of the diverse functional properties of CD49b subsets, we performed RNA sequencing (RNA-seq) on stem- and progenitor cell subsets and downstream lymphoid-primed MPPs (LMPPs) and granulocyte-monocyte progenitors (GMPs) (Table S1; Figures S6A and S6B). The investigated cells expressed the expected population-associated genes. Principal components analysis (PCA) showed that CD150$^+$ (CD49b$^-$, CD49b$^+$, and CD150^{int}), CD150$^-$, LMPP, and GMP populations were well separated (Figures 6A and S6C), but CD150$^+$ subsets did not cluster distinctly, suggesting high transcriptional overlap. Indeed, few significant differences were found between the CD49b$^-$ and the CD49b$^+$ subsets (Figure 6B). However, genes involved in preserving HSC dormancy and stemness properties, such as *Gfi1* (Hock et al., 2004) and *Dlk1-Meg3* (Qian et al., 2016), were upregulated in CD49b$^-$ cells, consistent with their superior self-renewal potential. Only five genes, including *Itga2* (*CD49b*), were significantly upregulated in CD49b$^+$ cells (Figure 6B). Consistent with bulk RNA-seq data, uniform manifold approximation and projection (UMAP) analysis of single-cell RNA-seq (scRNA-seq) could not resolve the subsets, and only eight genes, including *Gfi1*, were differentially expressed between CD49b$^-$ and CD49b$^+$ cells (Figures 6C, S6D, and S7A).

To identify epigenetic changes associated with gene expression differences, we analyzed open chromatin using assay for transposase-accessible chromatin sequencing (ATAC-seq, Table S2, Figures S6E and S6F) (Corces et al., 2017). Chromatin accessibility at population-specific genes followed the expected patterns (Figures S6G and S6H). PCA revealed distinct LMPP and GMP clusters, whereas CD150$^+$ and CD150$^-$ populations clustered closely together, but, in contrast to RNA-seq data, formed distinguishable groups (Figure 6D), indicating that transcriptionally similar CD49b$^-$ and CD49b$^+$ subsets differ on the chromatin level.

To assess chromatin accessibility changes from HSCs to progenitors, we performed pairwise comparisons between the CD150$^+$, the LMPP, and the GMP populations, which created five clusters with significant differences (Figure 6E). Most regions with high accessibility in CD150$^+$ populations (clusters 1–2) became inaccessible at progenitor stages. Conversely, regions accessible in LMPPs (cluster 3) and GMPs (clusters 4–5) were mainly inaccessible in the CD150$^+$ group. Gene ontology (GO) analysis showed that high-accessibility regions in progenitors (clusters 3–5) were associated with myeloid and immune cell regulation,

(D) Donor contribution to platelets, erythrocytes, and myeloid, B, T, and natural killer cells in PB from single-cell transplantation (n_{CD49b^-} = 10 mice, n_{CD49b^+} = 6 mice, two independent experiments).
(E) Total donor contribution (top) and relative contribution to myeloid, B, T, and natural killer cells (bottom) in PB of CD49b$^-$ and CD49b$^+$ single-cell-transplanted mice, 9 months post-transplantation (n_{CD49b^-} = 10 mice, n_{CD49b^+} = 6 mice, two independent experiments).
(F) Overview of CD49b$^-$ and CD49b$^+$ primary reconstituted single-cell-transplanted mice with LT activity from (A). The categorized lineage bias and number of mice with donor contribution to PB and phenotypic HSCs (LSKFlt-3$^-$CD48$^-$CD150$^+$ or LSKCD48$^-$CD150$^+$) 5–12 months post-transplantation, in primary and secondary transplantation, are indicated.
(G) Donor contribution to phenotypic HSCs (LSKFlt-3$^-$CD48$^-$CD150$^+$ or LSKCD48$^-$CD150$^+$) in secondary transplantation with whole BM cells, 5–6 months post-transplantation (n_{CD49b^-} = 50, n_{CD49b^+} = 8 mice, five independent experiments). ns, not significant.
(H) Donor contribution to phenotypic lineage-biased HSC subsets, 5–12 months post-transplantation, from single-cell-transplanted mice (n_{CD49b^-} = 27 mice, n_{CD49b^+} = 17 mice, five independent experiments). The number of reconstituted primary donor mice out of all mice analyzed is indicated in (C), (G) and (H). Mean ± SD is shown (C, D, G, H). ns, not significant. See also Figures S4 and S5.

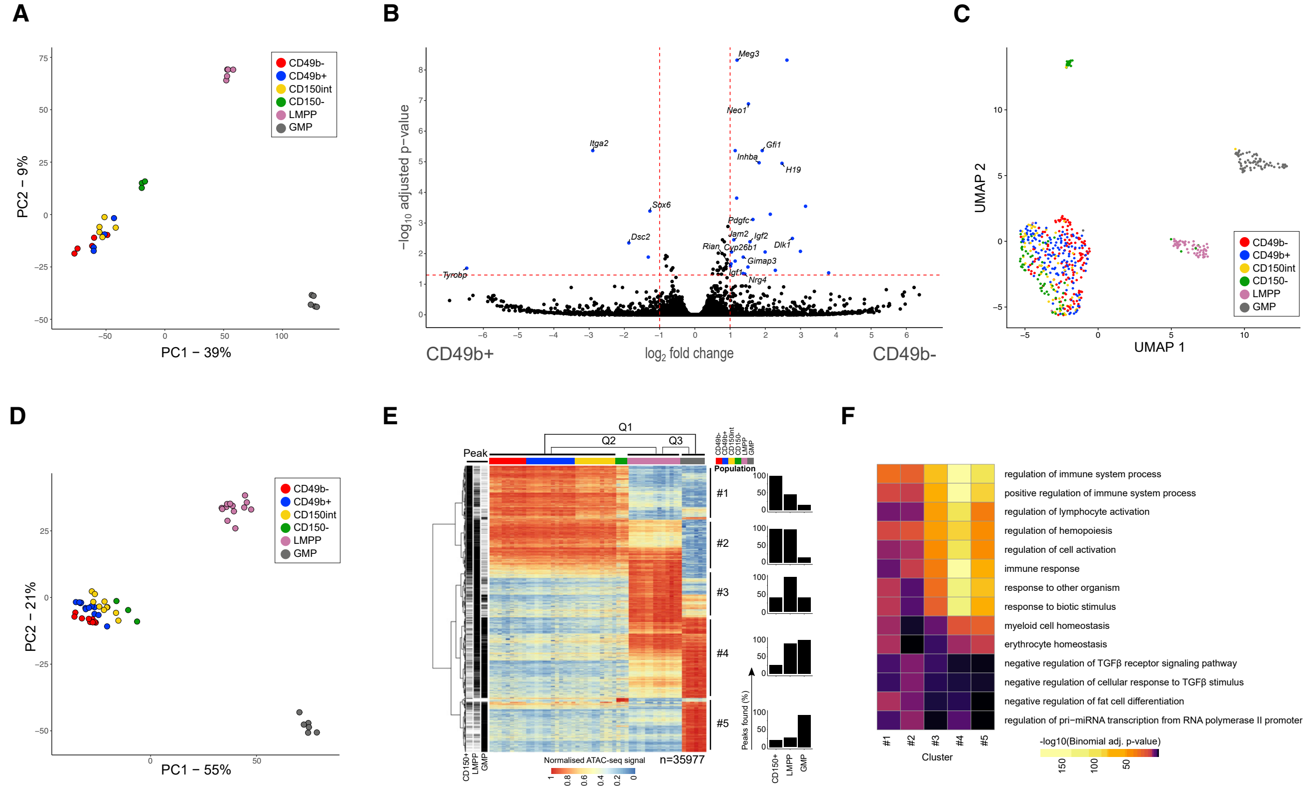

Figure 6. The CD49b$^-$ and CD49b$^+$ subsets are transcriptionally similar but epigenetically distinct

(A) PCA of bulk RNA-seq data ($n_{CD49b^-} = 5$, $n_{CD49b^+} = 4$, $n_{CD150^{int}} = 5$, $n_{CD150^-} = 3$, $n_{LMPP} = 5$, $n_{GMP} = 5$ replicates, five independent experiments).

(B) Volcano plot of differential expression between CD49b$^-$ and CD49b$^+$ subsets. Differentially expressed genes ($p_{adj} < 0.05$, |fold change| > 2) are marked in blue, with selected genes annotated.

(C) UMAP visualization of single-cell RNA-seq data colored by cell population ($n_{CD49b^-} = 135$, $n_{CD49b^+} = 146$, $n_{CD150^{int}} = 59$, $n_{CD150^-} = 77$, $n_{LMPP} = 57$, $n_{GMP} = 74$ cells, two independent experiments).

(D) PCA of ATAC-seq data ($n_{CD49b^-} = 9$, $n_{CD49b^+} = 12$, $n_{CD150^{int}} = 10$, $n_{CD150^-} = 3$, $n_{LMPP} = 13$, $n_{GMP} = 6$ replicates, seven independent experiments).

(E) Heatmap (left) of row-normalized chromatin accessibility for differential regions ($p_{adj} < 1 \times 10^{-4}$, |fold change| > 2) in pairwise comparisons between CD150$^+$, LMPP, and GMP populations. Regions are divided into five clusters based on hierarchical clustering. Bar graphs (right) show how many peaks were called in each cluster.

(F) GO enrichment analysis of clusters 1–5. The top three significantly enriched terms for each cluster from the GO biological process are shown. See also Figures S6 and S7.

whereas regions in the CD150$^+$ group (clusters 1–2) were associated with transforming growth factor β signaling, involved in regulation of HSC quiescence (Figure 6F) (Wang et al., 2018). These findings suggested that generation of downstream progenitors from CD150$^+$ HSCs is associated with extensive chromatin remodeling.

The CD49b$^-$ and CD49b$^+$ subsets exhibit distinct epigenetic changes

Since the CD150$^+$ subsets formed distinct but closely related clusters (Figure 6D), we assessed the overlap of open chromatin. Most accessible regions were shared between the populations (87%; Figure 7A). Differentially accessible regions were preferentially localized in non-promoter regions (Figure 7B). There was a general increase in open chromatin regions in CD49b$^+$ compared with CD49b$^-$ cells (Figure 7C). Most regions with higher accessibility in CD49b$^-$ cells had reduced accessibility in CD49b$^+$ and downstream populations. Furthermore, the overall chromatin accessibilities in CD49b$^+$, CD150^{int}, and CD150$^-$ populations were similar, indicating that the CD49b$^-$ subset is developmentally upstream of the other populations. Consistent with RNA-seq data (Figure 6B), regions with increased accessibility in the CD49b$^-$ cells were found proximal to *Gfi1* (Hock et al., 2004) and *Dlk1* (Qian et al., 2016) (Figure S7B). Conversely, in CD49b$^+$ cells we

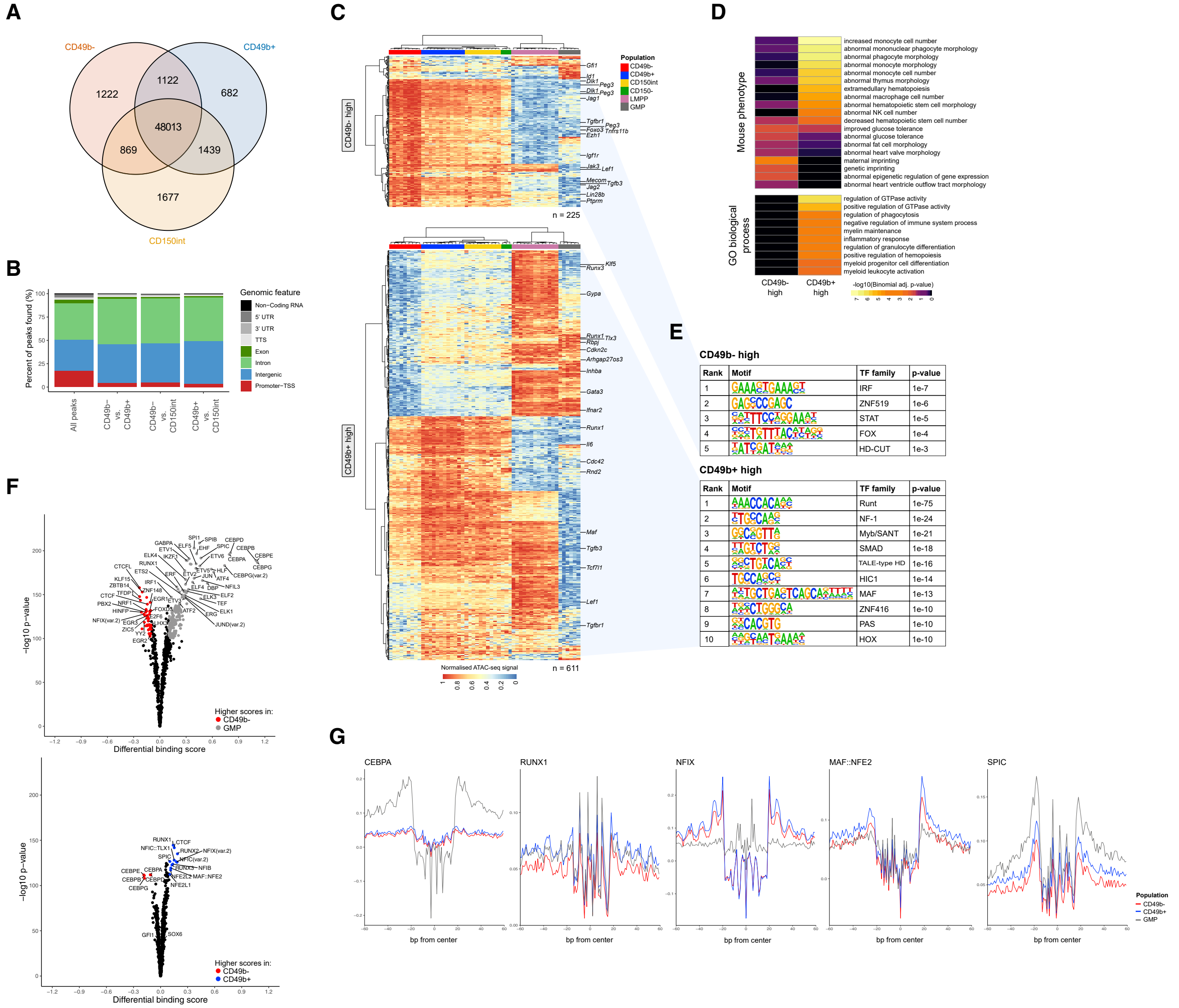

Figure 7. The CD49b^{-} and CD49b^{+} subsets exhibit distinct epigenetic changes

(A) Overlap of open chromatin regions between CD150^{+} populations. Peaks with a normalized read count of >1.5 in more than one-third of samples were considered found.

(B) Genomic feature distribution of regions with differential accessibility (p_{adj} < 0.01) and all regions as a reference ("all peaks").

(C) Heatmap of row-normalized chromatin accessibility for regions with differential accessibility (p_{adj} < 0.01) between CD49b^{-} (top; n = 225 peaks) and CD49b^{+} (bottom; n = 611 peaks) subsets. For selected regions the nearest gene is indicated.

(D) GO enrichment analysis of regions with differential accessibility between CD49b^{-} and CD49b^{+} subsets. The top 10 significantly enriched terms from the mouse phenotype and GO biological process are shown.

(E) Transcription factor (TF) families with enriched binding motifs (q <0.01, top 10) in regions with increased accessibility in CD49b^{-} (top) or CD49b^{+} (bottom) cells.

(F) Volcano plots of differential TF binding. Transcription factors with differential binding activity (differential binding score >0.1, $p < 1 \times 10^{-100}$) are colored and selectively annotated.

(G) Aggregated footprint plots for TFs with differential binding. See also Figure S7.

found regions with higher accessibility adjacent to the hematopoietic regulators *Runx1*, *Runx3*, and *Maf* (Mevel et al., 2019; Sarrazin et al., 2009), but their expression was not significantly changed (Figures 7C and S7B). Gene ontology analysis (Figure 7D) showed that regions with increased accessibility in CD49b^{-} were associated with pathways

involved in genetic imprinting, which regulates HSC quiescence (Qian et al., 2016). In contrast, processes regulating hematopoietic cell numbers, differentiation, activation, and GTPase activity (Mulloy et al., 2010) were associated with regions with higher accessibility in CD49b$^+$. These findings are consistent with the cell-cycle active and proliferative nature of CD49b$^+$ cells compared with the more quiescent CD49b$^-$ cells.

To identify potential transcription factors (TFs) responsible for chromatin accessibility differences between the CD49b subsets, we performed motif enrichment analysis (Figure 7E). TF binding sites (TFBSs) associated with the FOX (Tothova and Gilliland, 2007) and STAT (Dorritie et al., 2014) families were among the most enriched motifs in CD49b$^-$. Conversely, in CD49b$^+$ cells, HOX (Alharbi et al., 2013) and RUNX (Mevel et al., 2019) family TFBSs were enriched (Figure 7E). Moreover, we performed a genome-wide analysis of TF occupancy by footprinting analysis (Figures 7F and 7G). Differential TF binding plots showed large differences in TF binding between CD49b$^-$ and GMPs, which included the myeloid-associated C/EBP-family and SPI1 (PU.1) TFs (Tenen et al., 1997). In contrast, CD49b$^-$ and CD49b$^+$ had few TF binding differences. Consistent with the motif enrichment analysis, RUNX (RUNX1–3) (Mevel et al., 2019), NF-1 (including NFIB, NFIC, NFIX) (Harris et al., 2015), SPIC (Li et al., 2015), and MAF:NFE2 (Gasiorek and Blank, 2015) sites had significantly higher binding scores in CD49b$^+$ cells (Figures 7F and 7G). Altogether, our findings indicated that differential functions of CD49b$^-$ and CD49b$^+$ cells may to a large extent be regulated by the same set of TFs.

DISCUSSION

It is well recognized that the HSC population is functionally diverse, with subtypes differing in propensity of blood cell differentiation and in self-renewal ability and lifespan (Eaves, 2015; Wilkinson et al., 2020). Although HSC heterogeneity is recognized, insight into the molecular mechanisms underlying HSC diversity is lacking due to limitations in purifying homogeneous HSC subtypes. Here, we have reassessed the expression of cell-surface markers suggested to define HSCs to explore functional heterogeneity. We identified CD49b as a candidate marker to subfractionate the phenotypic LSKCD34$^-$CD48$^-$CD150^{hi} compartment, enriched for M-bi and functional HSCs (Morita et al., 2010; Wilkinson et al., 2020). The CD49b$^-$ subset greatly improved the purity of M-bi cells and cells with the highest self-renewal activity. However, the L-bi phenotype was most common in CD49b$^+$ cells, with a subset exhibiting multilineage LT HSC activity. Notably, the CD49b$^+$ L-bi HSCs in this study are distinct from the previously described CD150$^-$ L-bi HSCs and γ and δ cells, which all showed a lymphoid-dominant repopulation pattern and limited self-renewal ability consistent with loss of LT HSC activity (Dykstra et al., 2007; Kent et al., 2009; Morita et al., 2010). Within the CD49b$^+$ fraction such lymphoid dominant cells were categorized as ST/transient cells. Furthermore, CD49b$^+$ cells described here were potent in generating platelets, erythrocytes, and lymphoid cells, but had low myeloid contribution, resulting in a lymphoid bias in the leukocyte compartment. Of note, the decline in platelet and erythrocyte reconstitution was associated with ST activity in lymphoid-dominant cells. There are several studies with findings compatible with the existence of multipotent LT L-bi HSCs (Challen et al., 2010; Dykstra et al., 2011; Oguro et al., 2013; Yamamoto et al., 2013). Our results, however, suggested that LT L-bi HSCs are infrequent in young adult mice, and although CD49b$^+$ cells could sustain LT repopulation, they are less durable than CD49b$^-$ cells.

CD49b has previously been used to identify and characterize ST (Wagers and Weissman, 2006) and IT HSCs (Benveniste et al., 2010) with finite self-renewal ability, indicating that CD49b expression is associated with reduced durability. Paradoxically, it has also been used to distinguish between reserve (CD49b$^-$) and primed (CD49b$^+$) HSCs (Zhao et al., 2019). While the previous studies showed opposing results in self-renewal ability of CD49b$^+$ cells, lineage bias was not investigated. We show that the contradictory findings could partly be explained by the lack of CD150 in the immunophenotyping strategies, which greatly enriches for LT HSCs (Kiel et al., 2005). Although there is a degree of overlap, the CD49b$^+$ cells in this study, which were identified from the primitive LSKCD34$^-$CD48$^-$CD150^{hi} compartment, exhibit distinct differences compared with the CD49b$^+$ cells in previous studies (Benveniste et al., 2010; Zhao et al., 2019), particularly in lineage bias. Our findings show that CD49b$^+$ are mainly defining L-bi cells and reconcile previous studies by showing that CD49b marks both HSCs and ST/transient cells.

The CD49b$^-$ subset was able to efficiently generate all other HSC subsets. The CD49b$^+$ subset was less effective in generating both stem- and progenitor cells, but was nevertheless still capable of giving rise to all phenotypically defined HSC subsets. This suggested that CD49b$^-$ are hierarchically superior to CD49b$^+$ cells, but also that a degree of interconversion may occur, which remains to be confirmed through functional analysis.

Our data indicated that a degree of functional heterogeneity remains, especially within the CD49b$^+$ subset. Although we were unable to detect any significant functional differences *in vitro* with CD41 and CD229 subfractionation, it remains to be determined whether these subfractions can resolve the residual functional heterogeneity *in vivo*.

Insights into the molecular mechanisms underlying HSC heterogeneity are largely lacking. Genome-wide expression analysis of the HSC population has previously shown heterogeneity among phenotypic HSCs, suggesting that transcriptional profiling may distinguish functionally diverse HSC subsets (Challen et al., 2010; Haas et al., 2018; Wilson et al., 2015). Surprisingly, we observed high transcriptional overlap on both the bulk and the single-cell level between functionally different CD49b^{-} and CD49b^{+} cells. These findings suggested that functional differences between the HSC subsets may be determined by only a few genes or that RNA-seq could not reveal combinatorial consequences of small gene expression changes. To investigate the epigenetic changes associated with the subtle gene expression differences, we surveyed the genome-wide chromatin accessibility landscape of CD49b^{-} and CD49b^{+} cells. We observed distinct profiles, which, in agreement with previous studies, differed predominantly in promoter distal regions (Martin et al., 2021; Yu et al., 2017). We found a general increase in open chromatin associated with processes of an activated and proliferative cellular state in CD49b^{+}. Conversely, in CD49b^{-} cells, open chromatin regions were associated with processes involved in quiescence and dormancy. These results implied that CD49b^{-} and CD49b^{+} cells may have different epigenetic configurations priming them for the distinct *in vivo* functional behavior. These findings highlight the need to unfold specific regulators and epigenetic mechanisms that directly affect HSC function and diversity.

Collectively, we have shown that CD49b can be used to further enrich LT M-bi HSCs to high purity, by segregating a subset of multipotent CD49b^{+} cells with lymphoid bias. Although L-bi cells were commonly associated with finite self-renewal, a small number of them could sustain LT, which correlated with the persistence of platelet and erythroid repopulation. Despite diverse functional characteristics, CD49b^{-} and CD49b^{+} HSCs were transcriptionally similar but epigenetically different. Overall, our studies highlight the different facets of the complex structure of the HSC compartment, composed of diverse HSCs with distinct functional behaviors that are likely regulated through epigenetic mechanisms as they sustain life-long hematopoiesis.

EXPERIMENTAL PROCEDURES

See supplemental information for details.

Animals

Female and male C57BL/6J mice (8–17 weeks) were used. Gata-1 eGFP (Drissen et al., 2016) mice were backcrossed more than eight generations to a C57BL/6J background. All experiments were approved by the regional ethics committee.

Hematopoietic cell preparation

BM cell suspensions were prepared by bone crushing. Cells were Fc-blocked and stained with antibodies against cell-surface antigens (Table S3). For HSC detection, BM cells were enriched by CD117 immunomagnetic separation (Miltenyi Biotec) before antibody staining. Platelets, erythrocytes, and leukocytes were isolated from PB samples followed by antibody staining as described previously (Carrelha et al., 2018; Luc et al., 2016). See Table S4 for immunophenotypes.

Transplantation experiments

Test cells were intravenously injected into lethally irradiated CD45.1 mice with 200,000–250,000 BM CD45.1^{+} support cells. PB analysis was periodically done 6–9 months post-transplantation.

In vitro assays

Myeloid and B cell potential was evaluated with OP9 co-culture assay (Luc et al., 2012). Megakaryocyte potential was evaluated by manually plating one cell/well into 60-well plates and assessed after 13 days. Erythroid potential was evaluated by plating 30 HSCs in complete methylcellulose (GF M3434; STEMCELL Technologies) and evaluated after 12 days with 2,7-diaminofluorene staining (Merck) (Luc et al., 2012). Cell division kinetics was assessed by tracking cell divisions of single cells on days 1–4 post-sorting (Luc et al., 2016). See Table S5 for culture conditions.

Cell-cycle and proliferation assays

Ki-67 staining was done with a Cytofix/Cytoperm Kit (BD Biosciences). One dose of BrdU was given by intraperitoneal injection (50 μg/g body weight, BD), followed by oral administration (800 μg/mL, Merck) for 3 days. BrdU visualization was performed with a BrdU Flow Kit (BD Biosciences).

RNA-seq and ATAC-seq

Bulk RNA-seq was performed with 250–500 sorted cells. Libraries were paired-end sequenced (2 × 41 cycles) on NextSeq 500 (Illumina). scRNA-seq, with SmartSeq2, was performed by sorting single cells into 384-well plates, and libraries were sequenced on HiSeq3000 (Illumina) using dual indexing and single 50-bp reads as previously described (Vanlandewijck and Betsholtz, 2018).

ATAC-seq was performed with 500 sorted cells using the Omni-ATAC protocol with modifications (Corces et al., 2017). Libraries were paired-end sequenced (2 × 41 cycles) using NextSeq 550 (Illumina).

Statistical methods

Statistical analysis was done in GraphPad Prism v.9.2.0. Non-parametric tests were performed using the Mann-Whitney test or the Kruskal-Wallis with Dunn's multiple comparison test. The means ± SD are shown.

Data availability

RNA-seq and ATAC-seq data have been deposited in the European Nucleotide Archive (ENA). The accession number for the RNA-seq and ATAC-seq data reported in this paper is ENA: PRJEB47791.

SUPPLEMENTAL INFORMATION

Supplemental information can be found online at https://doi.org/10.1016/j.stemcr.2022.05.014.

AUTHOR CONTRIBUTIONS

E.S., A.-S.J., Ö.D., and S.L. performed cell and mouse experiments. P.K. performed ATAC-seq. N.S.F. and C.G. performed RNA-seq. G.M. and M.V. performed and analyzed scRNA-seq. J.H. performed bioinformatics analysis. T.-Y.S., H.B., and C.L.T. assisted in experiments. S.L. designed the project and wrote the paper together with R.M., E.S., and J.H. S.H.O., R.M., and S.L. supervised the work. All authors reviewed the manuscript before submission.

ACKNOWLEDGMENTS

The authors thank Prof. S.E. Jacobsen and Dr. P. Woll, Karolinska Institutet (KI), for helpful discussions and Prof. C. Nerlov, University of Oxford, for the Gata-1 eGFP mouse strain. The authors thank Prof. J. Dillner, the Bioinformatics and Expression Analysis core facility, and the Single Cell core facility of Flemingsberg Campus, KI, for sequencing support and the Preclinical Laboratory, Karolinska University Hospital, and KI MedH Flow Cytometry Core Facility for their services. The computations and data storage were enabled by resources provided by the Swedish National Infrastructure for Computing (SNIC) at Uppmax, partially funded by the Swedish Research Council (2018-05973). S.L. is supported by a Wallenberg Academy Fellow award (2016.0131) and by the Swedish Childhood Cancer Fund (TJ2017-0074, PR2017-0047). S.H.O. is an investigator of the Howard Hughes Medical Institute (HHMI), J.H. and T.-Y.S. are supported by KI Doctoral Education grants. This work was funded by the European Hematology Association, the Swedish Cancer Society (CAN2017/583, 20 1062 PjF), the Swedish Research Council (2016-02331), the Strategic Research Area (SFO) in Stem Cell and Regenerative Medicine, Åke Olsson Foundation, Åke Wiberg Foundation, and King Gustav V Jubilee Fund.

CONFLICTS OF INTERESTS

The authors declare no competing interests.

Received: January 9, 2022
Revised: May 19, 2022
Accepted: May 19, 2022
Published: June 16, 2022

REFERENCES

Adolfsson, J., Borge, O.J., Bryder, D., Theilgaard-Mönch, K., Åstrand-Grundström, I., Sitnicka, E., Sasaki, Y., and Jacobsen, S.E.W. (2001). Upregulation of Flt3 expression within the bone marrow Lin−Sca1+c-kit+ stem cell compartment is accompanied by loss of self-renewal capacity. Immunity *15*, 659–669. https://doi.org/10.1016/s1074-7613(01)00220-5.

Alharbi, R.A., Pettengell, R., Pandha, H.S., and Morgan, R. (2013). The role of HOX genes in normal hematopoiesis and acute leukemia. Leukemia *27*, 1000–1008. https://doi.org/10.1038/leu.2012.356.

Arai, F., Hirao, A., Ohmura, M., Sato, H., Matsuoka, S., Takubo, K., Ito, K., Koh, G.Y., and Suda, T. (2004). Tie2/Angiopoietin-1 signaling regulates hematopoietic stem cell quiescence in the bone marrow niche. Cell *118*, 149–161. https://doi.org/10.1016/j.cell.2004.07.004.

Balazs, A.B., Fabian, A.J., Esmon, C.T., and Mulligan, R.C. (2006). Endothelial protein C receptor (CD201) explicitly identifies hematopoietic stem cells in murine bone marrow. Blood *107*, 2317–2321. https://doi.org/10.1182/blood-2005-06-2249.

Benveniste, P., Frelin, C., Janmohamed, S., Barbara, M., Herrington, R., Hyam, D., and Iscove, N.N. (2010). Intermediate-term hematopoietic stem cells with extended but time-limited reconstitution potential. Cell Stem Cell *6*, 48–58. https://doi.org/10.1016/j.stem.2009.11.014.

Carrelha, J., Meng, Y., Kettyle, L.M., Luis, T.C., Norfo, R., Alcolea, V., Boukarabila, H., Grasso, F., Gambardella, A., Grover, A., et al. (2018). Hierarchically related lineage-restricted fates of multipotent haematopoietic stem cells. Nature *554*, 106–111. https://doi.org/10.1038/nature25455.

Challen, G.A., Boles, N.C., Chambers, S.M., and Goodell, M.A. (2010). Distinct hematopoietic stem cell subtypes are differentially regulated by TGF-β1. Cell Stem Cell *6*, 265–278. https://doi.org/10.1016/j.stem.2010.02.002.

Corces, M.R., Trevino, A.E., Hamilton, E.G., Greenside, P.G., Sinnott-Armstrong, N.A., Vesuna, S., Satpathy, A.T., Rubin, A.J., Montine, K.S., Wu, B., et al. (2017). An improved ATAC-seq protocol reduces background and enables interrogation of frozen tissues. Nat. Methods *14*, 959–962. https://doi.org/10.1038/nmeth.4396.

Dorritie, K.A., McCubrey, J.A., and Johnson, D.E. (2014). STAT transcription factors in hematopoiesis and leukemogenesis: opportunities for therapeutic intervention. Leukemia *28*, 248–257. https://doi.org/10.1038/leu.2013.192.

Drissen, R., Buza-Vidas, N., Woll, P., Thongjuea, S., Gambardella, A., Giustacchini, A., Mancini, E., Zriwil, A., Lutteropp, M., Grover, A., et al. (2016). Distinct myeloid progenitor differentiation pathways identified through single cell RNA sequencing. Nat. Immunol. *17*, 666–676. https://doi.org/10.1038/ni.3412.

Dykstra, B., Kent, D., Bowie, M., McCaffrey, L., Hamilton, M., Lyons, K., Lee, S.-J., Brinkman, R., and Eaves, C. (2007). Long-term propagation of distinct hematopoietic differentiation programs in vivo. Cell Stem Cell *1*, 218–229. https://doi.org/10.1016/j.stem.2007.05.015.

Dykstra, B., Olthof, S., Schreuder, J., Ritsema, M., and de Haan, G. (2011). Clonal analysis reveals multiple functional defects of aged murine hematopoietic stem cells. J. Exp. Med. *208*, 2691–2703. https://doi.org/10.1084/jem.20111490.

Eaves, C.J. (2015). Hematopoietic stem cells: concepts, definitions, and the new reality. Blood *125*, 2605–2613. https://doi.org/10.1182/blood-2014-12-570200.

Gasiorek, J.J., and Blank, V. (2015). Regulation and function of the NFE2 transcription factor in hematopoietic and non-hematopoietic cells. Cell. Mol. Life Sci. *72*, 2323–2335. https://doi.org/10.1007/s00018-015-1866-6.

Haas, S., Trumpp, A., and Milsom, M.D. (2018). Causes and consequences of hematopoietic stem cell heterogeneity. Cell Stem Cell *22*, 627–638. https://doi.org/10.1016/j.stem.2018.04.003.

Harris, L., Genovesi, L.A., Gronostajski, R.M., Wainwright, B.J., and Piper, M. (2015). Nuclear factor one transcription factors: divergent functions in developmental versus adult stem cell populations. Dev. Dynam. *244*, 227–238. https://doi.org/10.1002/dvdy.24182.

Hock, H., Hamblen, M.J., Rooke, H.M., Schindler, J.W., Saleque, S., Fujiwara, Y., and Orkin, S.H. (2004). Gfi-1 restricts proliferation and preserves functional integrity of haematopoietic stem cells. Nature *431*, 1002–1007. https://doi.org/10.1038/nature02994.

Karlsson, G., Rörby, E., Pina, C., Soneji, S., Reckzeh, K., Miharada, K., Karlsson, C., Guo, Y., Fugazza, C., Gupta, R., et al. (2013). The tetraspanin CD9 affords high-purity capture of all murine hematopoietic stem cells. Cell Rep. *4*, 642–648. https://doi.org/10.1016/j.celrep.2013.07.020.

Kent, D.G., Copley, M.R., Benz, C., Wöhrer, S., Dykstra, B.J., Ma, E., Cheyne, J., Zhao, Y., Bowie, M.B., Zhao, Y., et al. (2009). Prospective isolation and molecular characterization of hematopoietic stem cells with durable self-renewal potential. Blood *113*, 6342–6350. https://doi.org/10.1182/blood-2008-12-192054.

Kiel, M.J., Yilmaz, Ö.H., Iwashita, T., Yilmaz, O.H., Terhorst, C., and Morrison, S.J. (2005). SLAM family receptors distinguish hematopoietic stem and progenitor cells and reveal endothelial niches for stem cells. Cell *121*, 1109–1121. https://doi.org/10.1016/j.cell.2005.05.026.

Li, S.K.H., Solomon, L.A., Fulkerson, P.C., and DeKoter, R.P. (2015). Identification of a negative regulatory role for spi-C in the murine B cell lineage. J. Immunol. *194*, 3798–3807. https://doi.org/10.4049/jimmunol.1402432.

Luc, S., Luis, T.C., Boukarabila, H., Macaulay, I.C., Buza-Vidas, N., Bouriez-Jones, T., Lutteropp, M., Woll, P.S., Loughran, S.J., Mead, A.J., et al. (2012). The earliest thymic T cell progenitors sustain B cell and myeloid lineage potential. Nat. Immunol. *13*, 412–419. https://doi.org/10.1038/ni.2255.

Luc, S., Huang, J., McEldoon, J.L., Somuncular, E., Li, D., Rhodes, C., Mamoor, S., Hou, S., Xu, J., and Orkin, S.H. (2016). Bcl11a deficiency leads to hematopoietic stem cell defects with an aging-like phenotype. Cell Rep. *16*, 3181–3194. https://doi.org/10.1016/j.celrep.2016.08.064.

Mann, M., Mehta, A., de Boer, C.G., Kowalczyk, M.S., Lee, K., Haldeman, P., Rogel, N., Knecht, A.R., Farouq, D., Regev, A., and Baltimore, D. (2018). Heterogeneous responses of hematopoietic stem cells to inflammatory stimuli are altered with age. Cell Rep. *25*, 2992–3005.e5. https://doi.org/10.1016/j.celrep.2018.11.056.

Martin, E.W., Krietsch, J., Reggiardo, R.E., Sousae, R., Kim, D.H., and Forsberg, E.C. (2021). Chromatin accessibility maps provide evidence of multilineage gene priming in hematopoietic stem cells. Epigenet. Chromatin *14*, 2. https://doi.org/10.1186/s13072-020-00377-1.

Mevel, R., Draper, J.E., Lie-a-Ling, M., Kouskoff, V., and Lacaud, G. (2019). RUNX transcription factors: orchestrators of development. Development *146*, dev148296. https://doi.org/10.1242/dev.148296.

Morita, Y., Ema, H., and Nakauchi, H. (2010). Heterogeneity and hierarchy within the most primitive hematopoietic stem cell compartment. J. Exp. Med. *207*, 1173–1182. https://doi.org/10.1084/jem.20091318.

Müller-Sieburg, C.E., Cho, R.H., Thoman, M., Adkins, B., and Sieburg, H.B. (2002). Deterministic regulation of hematopoietic stem cell self-renewal and differentiation. Blood *100*, 1302–1309. https://doi.org/10.1182/blood.v100.4.1302.h81602001302_1302_1309.

Muller-Sieburg, C.E., Cho, R.H., Karlsson, L., Huang, J.-F., and Sieburg, H.B. (2004). Myeloid-biased hematopoietic stem cells have extensive self-renewal capacity but generate diminished lymphoid progeny with impaired IL-7 responsiveness. Blood *103*, 4111–4118. https://doi.org/10.1182/blood-2003-10-3448.

Mulloy, J.C., Cancelas, J.A., Filippi, M.-D., Kalfa, T.A., Guo, F., and Zheng, Y. (2010). Rho GTPases in hematopoiesis and hemopathies. Blood *115*, 936–947. https://doi.org/10.1182/blood-2009-09-198127.

Oguro, H., Ding, L., and Morrison, S.J. (2013). SLAM family markers resolve functionally distinct subpopulations of hematopoietic stem cells and multipotent progenitors. Cell Stem Cell *13*, 102–116. https://doi.org/10.1016/j.stem.2013.05.014.

Qian, P., He, X.C., Paulson, A., Li, Z., Tao, F., Perry, J.M., Guo, F., Zhao, M., Zhi, L., Venkatraman, A., et al. (2016). The dlk1-Gtl2 locus preserves LT-HSC function by inhibiting the PI3K-mTOR pathway to restrict mitochondrial metabolism. Cell Stem Cell *18*, 214–228. https://doi.org/10.1016/j.stem.2015.11.001.

Sanjuan-Pla, A., Macaulay, I.C., Jensen, C.T., Woll, P.S., Luis, T.C., Mead, A., Moore, S., Carella, C., Matsuoka, S., Jones, T.B., et al. (2013). Platelet-biased stem cells reside at the apex of the haematopoietic stem-cell hierarchy. Nature *502*, 232–236. https://doi.org/10.1038/nature12495.

Sarrazin, S., Mossadegh-Keller, N., Fukao, T., Aziz, A., Mourcin, F., Vanhille, L., Kelly Modis, L., Kastner, P., Chan, S., Duprez, E., et al. (2009). MafB restricts M-CSF-dependent myeloid commitment divisions of hematopoietic stem cells. Cell *138*, 300–313. https://doi.org/10.1016/j.cell.2009.04.057.

Shimazu, T., Iida, R., Zhang, Q., Welner, R.S., Medina, K.L., Alberola-lla, J., and Kincade, P.W. (2012). CD86 is expressed on murine hematopoietic stem cells and denotes lymphopoietic potential. Blood *119*, 4889–4897. https://doi.org/10.1182/blood-2011-10-388736.

Sudo, T., Yokota, T., Oritani, K., Satoh, Y., Sugiyama, T., Ishida, T., Shibayama, H., Ezoe, S., Fujita, N., Tanaka, H., et al. (2012). The endothelial antigen ESAM monitors hematopoietic stem cell status between quiescence and self-renewal. J. Immunol. *189*, 200–210. https://doi.org/10.4049/jimmunol.1200056.

Tenen, D.G., Hromas, R., Licht, J.D., and Zhang, D.-E. (1997). Transcription factors, normal myeloid development, and leukemia. Blood *90*, 489–519. https://doi.org/10.1182/blood.v90.2.489.489_489_519.

Tothova, Z., and Gilliland, D.G. (2007). FoxO transcription factors and stem cell homeostasis: insights from the hematopoietic system. Cell Stem Cell *1*, 140–152. https://doi.org/10.1016/j.stem.2007.07.017.

Vanlandewijck, M., and Betsholtz, C. (2018). Single-cell mRNA sequencing of the mouse brain vasculature. Methods Mol. Biol. *1846*, 309–324. https://doi.org/10.1007/978-1-4939-8712-2_21.

Wagers, A.J., and Weissman, I.L. (2006). Differential expression of α2 integrin separates long-term and short-term reconstituting lin−/loThy1.1loc-kit+ sca-1+ hematopoietic stem cells. Stem Cell. *24*, 1087–1094. https://doi.org/10.1634/stemcells.2005-0396.

Wang, X., Dong, F., Zhang, S., Yang, W., Yu, W., Wang, Z., Zhang, S., Wang, J., Ma, S., Wu, P., et al. (2018). TGF-β1 negatively regulates the number and function of hematopoietic stem cells. Stem Cell Rep. *11*, 274–287. https://doi.org/10.1016/j.stemcr.2018.05.017.

Wilkinson, A.C., Igarashi, K.J., and Nakauchi, H. (2020). Haematopoietic stem cell self-renewal in vivo and ex vivo. Nat. Rev. Genet. *21*, 541–554. https://doi.org/10.1038/s41576-020-0241-0.

Wilson, N.K., Kent, D.G., Buettner, F., Shehata, M., Macaulay, I.C., Calero-Nieto, F.J., Sánchez Castillo, M., Castillo, M.S., Oedekoven, C.A., Diamanti, E., et al. (2015). Combined single-cell functional and gene expression analysis resolves heterogeneity within stem cell populations. Cell Stem Cell *16*, 712–724. https://doi.org/10.1016/j.stem.2015.04.004.

Yamamoto, R., Morita, Y., Ooehara, J., Hamanaka, S., Onodera, M., Rudolph, K.L., Ema, H., and Nakauchi, H. (2013). Clonal analysis unveils self-renewing lineage-restricted progenitors generated directly from hematopoietic stem cells. Cell *154*, 1112–1126. https://doi.org/10.1016/j.cell.2013.08.007.

Yamamoto, R., Wilkinson, A.C., Ooehara, J., Lan, X., Lai, C.-Y., Nakauchi, Y., Pritchard, J.K., and Nakauchi, H. (2018). Large-scale clonal analysis resolves aging of the mouse hematopoietic stem cell compartment. Cell Stem Cell *22*, 600–607.e4. https://doi.org/10.1016/j.stem.2018.03.013.

Yu, X., Wu, C., Bhavanasi, D., Wang, H., Gregory, B.D., and Huang, J. (2017). Chromatin dynamics during the differentiation of long-term hematopoietic stem cells to multipotent progenitors. Blood Adv. *1*, 887–898. https://doi.org/10.1182/bloodadvances.2016003384.

Zhao, M., Tao, F., Venkatraman, A., Li, Z., Smith, S.E., Unruh, J., Chen, S., Ward, C., Qian, P., Perry, J.M., et al. (2019). N-Cadherin-Expressing bone and marrow stromal progenitor cells maintain reserve hematopoietic stem cells. Cell Rep. *26*, 652–669.e6. https://doi.org/10.1016/j.celrep.2018.12.093.

Stem Cell Reports, Volume *17*

Supplemental Information

CD49b identifies functionally and epigenetically distinct subsets of lineage-biased hematopoietic stem cells

Ece Somuncular, Julia Hauenstein, Prajakta Khalkar, Anne-Sofie Johansson, Özge Dumral, Nicolai S. Frengen, Charlotte Gustafsson, Giuseppe Mocci, Tsu-Yi Su, Hugo Brouwer, Christine L. Trautmann, Michael Vanlandewijck, Stuart H. Orkin, Robert Månsson, and Sidinh Luc

Figure S1, related to Figure 1

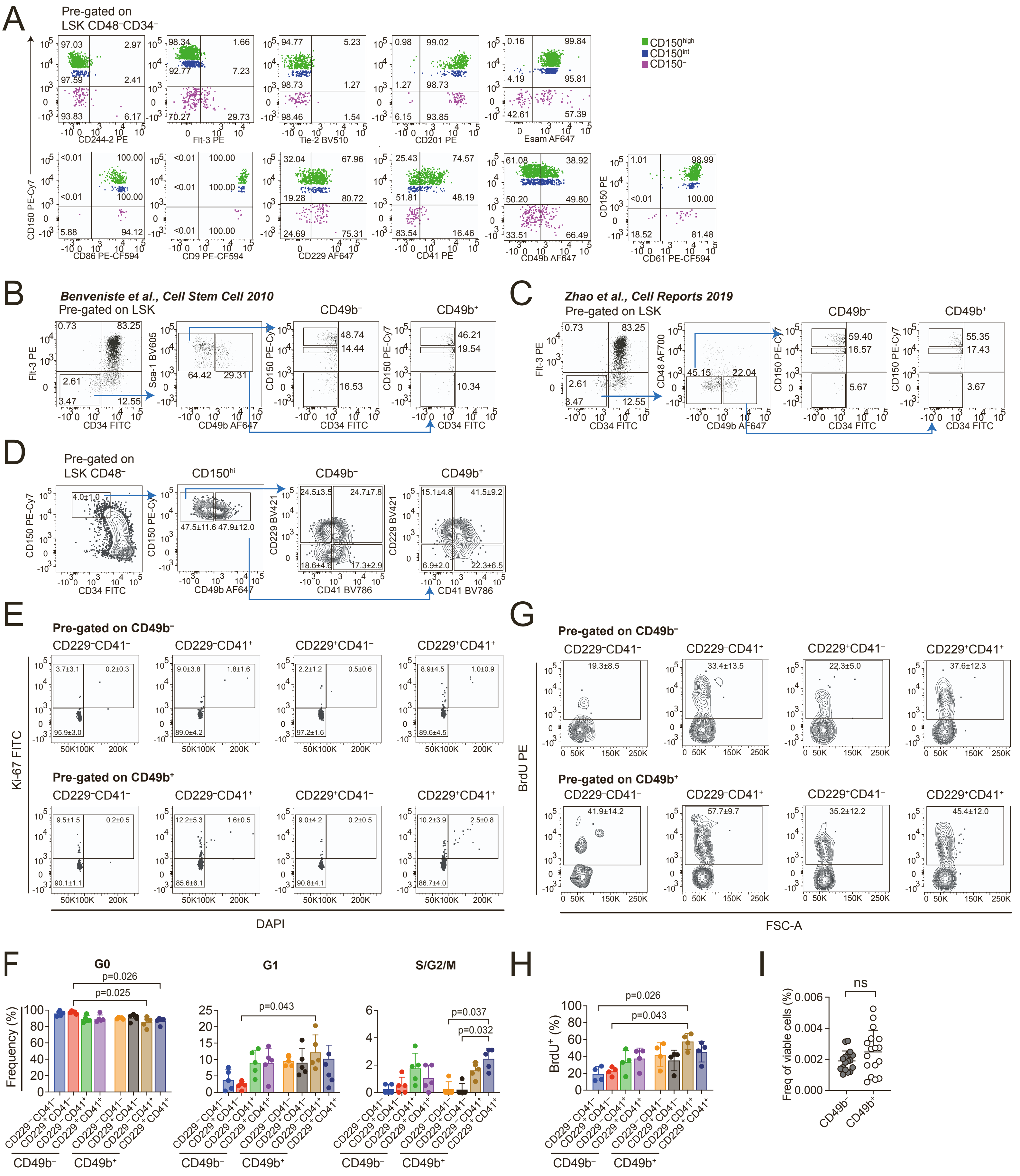

Figure S1, related to Figure 1

The hematopoietic stem cell compartment can be phenotypically subfractionated with CD49b

(A) Flow cytometry profiles of cell surface markers tested for further separation of phenotypic hematopoietic stem cell subsets defined as $Lineage^{-}Sca\text{-}1^{+}c\text{-}Kit^{+}$ (LSK) $CD48^{-}CD34^{-}CD150^{high}$ ($CD150^{hi}$), $LSKCD48^{-}CD34^{-}CD150^{intermediate}$ ($CD150^{int}$) and $LSKCD48^{-}CD34^{-}CD150^{-}$ ($CD150^{-}$), from c-Kit-enriched BM cells. All plots have been pre-gated on LSK $CD48^{-}CD34^{-}$ cells. The tested markers are shown on the x-axis. Frequencies of parent gates are shown.

(B) Flow cytometry profiles and gating strategy as shown in Benveniste *et al.* of long-term HSCs ($Lin^{-}Sca\text{-}1^{+}c\text{-}Kit^{+}CD34^{-}Flt\text{-}3^{-}CD49b^{-}$) and intermediate-term HSCs ($Lin^{-}Sca\text{-}1^{+}c\text{-}Kit^{+}CD34^{-}Flt\text{-}3^{-}CD49b^{+}$) and the subsequent analysis of CD150 expression.

(C) Flow cytometry profiles and gating strategy as shown in Zhao *et al.* of reserve HSCs ($Lin^{-}Sca\text{-}1^{+}c\text{-}Kit^{+}CD34^{-}Flt\text{-}3^{-}CD48^{-}CD49b^{-}$) and primed HSCs ($Lin^{-}Sca\text{-}1^{+}c\text{-}Kit^{+}CD34^{-}Flt\text{-}3^{-}CD48^{-}CD49b^{+}$) and the subsequent analysis of CD150 expression.

(D) Flow cytometry profiles showing further separation of $CD49b^{-}$ and $CD49b^{+}$ subsets with CD229 and CD41. Frequencies from parent gates are shown as mean $\pm$ SD from two experiments.

(E) Flow cytometry profiles of cell cycle analysis by Ki-67 staining. Frequencies are shown as mean $\pm$ SD from 5 biological replicates.

(F) Frequency of cells in G0, G1 and S/G2/M. Data collected from 5 biological replicates, one experiment.

(G) Flow cytometry profiles of BrdU labeling. Frequencies are shown as mean $\pm$ SD from 4 biological replicates.

(H) Frequency of proliferating ($BrdU^{+}$) cells. Frequencies are shown as mean $\pm$ SD from 4 biological replicates, one experiment.

(I) Total frequency of $CD49b^{-}$ and $CD49b^{+}$ HSCs in unfractionated BM (n = 17 mice, from 3 independent experiments). Data is presented as means $\pm$ SD. The statistical analysis was performed with Kruskal-Wallis with Dunn's multiple comparison test in (C) and (E) and Mann-Whitney test in (I). ns = not significant.

Figure S2, related to Figure 3

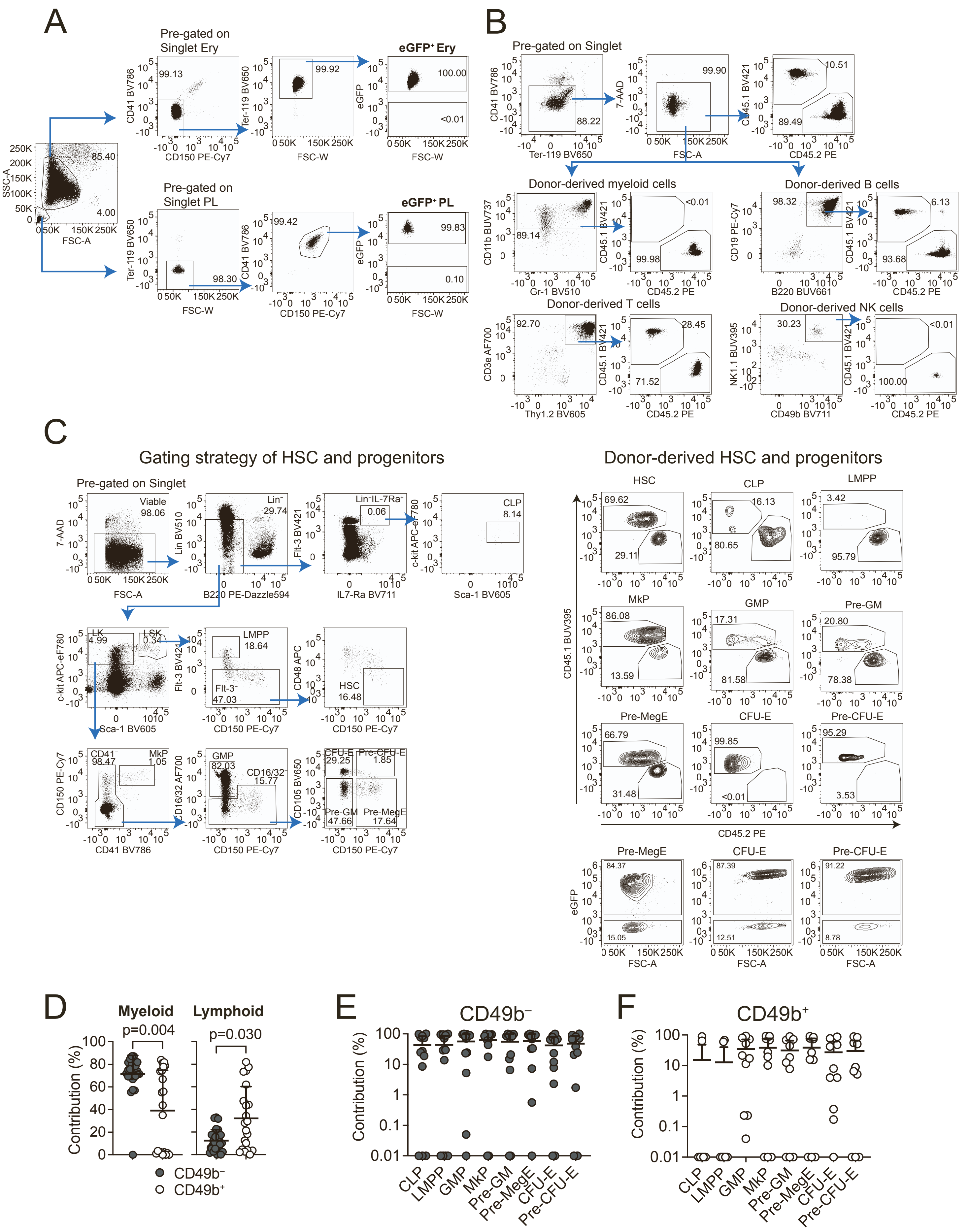

Figure S2, related to Figure 3

Analysis of hematopoietic populations from CD49b^{-} and CD49b^{+} transplanted mice

(A) Flow cytometry profiles and gating strategy for platelet-erythrocyte analysis from a representative peripheral blood sample. Donor-derived platelets (PL) were phenotypically defined as Ter-119^{-}CD41^{+}CD150^{+}*Gata-1*-eGFP^{+} and erythrocytes (Ery) as CD41^{-}CD150^{-}Ter-119^{+}*Gata-1*-eGFP^{+}. Frequencies of parent gates are shown.

(B) Flow cytometry profiles and gating strategy for mature blood lineage analysis in peripheral blood and BM. A representative peripheral blood sample is shown. Total donor-derived cells were phenotypically defined as Ter-119^{-}CD41^{-}CD45.2^{+}, donor-derived myeloid cells as Ter-119^{-}CD41^{-}B220^{-}CD19^{-}NK1.1^{-}CD49b^{-}CD3e^{-}Thy1.2^{-}CD11b^{+}CD45.2^{+}, donor-derived B cells as Ter-119^{-}CD41^{-}CD11b^{-}Gr-1^{-}CD3e^{-}Thy1.2^{-}NK1.1^{-}CD49b^{-}B220^{+}CD19^{+}CD45.2^{+}, donor-derived T cells as Ter-119^{-}CD41^{-}CD11b^{-}Gr-1^{-}B220^{-}CD19^{-}NK1.1^{-}CD49b^{-}CD3e^{+}Thy1.2^{+} CD45.2^{+} and donor-derived NK cells as Ter-119^{-}CD41^{-}CD11b^{-}Gr-1^{-}B220^{-}CD19^{-}CD3e^{-} Thy1.2^{-}NK1.1^{+}CD49b^{+}CD45.2^{+}. Frequencies of parent gates are shown.

(C) Flow cytometry profiles and gating strategy for HSC and progenitor analysis from two representative unfractionated BM samples. A representative mouse showing gating strategy and donor reconstitution with CD45.2^{+}, and a second mouse demonstrating donor reconstitution of Pre-Meg-E, and Pre-CFU-E, CFU-E with Gata-1 eGFP^{+} expression. Donor-derived common lymphoid progenitor (CLP) were phenotypically defined as Lin^{-} B220^{low}Sca-1^{+}c-Kit^{+}Flt-3^{+}IL-7Ra^{+}CD45.2^{+}, lymphoid-primed multipotent progenitor (LMPP) as Lin^{-}Sca-1^{+}c-Kit^{+} (LSK) Flt-3^{hi}CD45.2^{+}, megakaryocyte progenitor (MkP) as Lin^{-}Sca-1^{-}c-Kit^{+} (LK) CD150^{+}CD41^{+}CD45.2^{+}, granulocyte-monocyte progenitor (GMP) as LKCD41^{-}CD150^{-}CD16/32^{+} CD45.2^{+}, Pre-granulocyte-monocyte (Pre-GM) as LKCD41^{-}CD16/32^{-}CD150^{-}CD105^{-}CD45.2^{+}, Pre-megakaryocyte-erythrocyte (Pre-MegE) as LKCD41^{-}CD16/32^{-}CD150^{+}CD105^{-}CD45.2^{+} or alternatively as LKCD41^{-}CD16/32^{-}CD150^{+}CD105^{-}eGFP^{+}, Pre-colony forming unit erythroid (Pre-CFU-E) as LKCD41^{-}CD16/32^{-}CD150^{+}CD105^{+}CD45.2^{+} or alternatively as LKCD41^{-}CD16/32^{-}CD150^{+}CD105^{+} eGFP^{+}, Colony forming unit erythroid (CFU-E) as LKCD41^{-}CD16/32^{-}CD150^{-}CD105^{+}CD45.2^{+} or alternatively as LKCD41^{-}CD16/32^{-}CD150^{-}CD105^{+}eGFP^{+}, Hematopoietic stem cell (HSC) as LSKFlt-3^{-} CD48^{-}CD150^{+}CD45.2^{+} or alternatively LSKCD48^{-}CD150^{+}CD45.2^{+}. Frequencies of parent gates are shown.

(D) Frequency of donor contribution to myeloid cells and lymphoid cells (B cells, T cells and NK cells) in the BM, 5-6 months following transplantation of 5 cells of CD49b^{-} and CD49b^{+} HSC populations ($n_{CD49b^{-}}$ = 27 mice and $n_{CD49b^{+}}$ = 21 mice, from 3 experiments).

(E-F) Frequency of donor contribution to progenitor cells, CLP, LMPP, GMP, MkP, PreGM, PreMegE, CFU-E and Pre-CFU-E in the BM, 5-6 months following transplantation of 5 cells of CD49b^{-} (E) and CD49b^{+} (F) HSC populations ($n_{CD49b^{-}}$ = 12 mice and $n_{CD49b^{+}}$ = 10 mice from 2 experiments).

Data in (D-F) are presented as means ± SD. The statistical analysis was performed with Mann-Whitney test in (D).

Figure S3, related to Figure 4

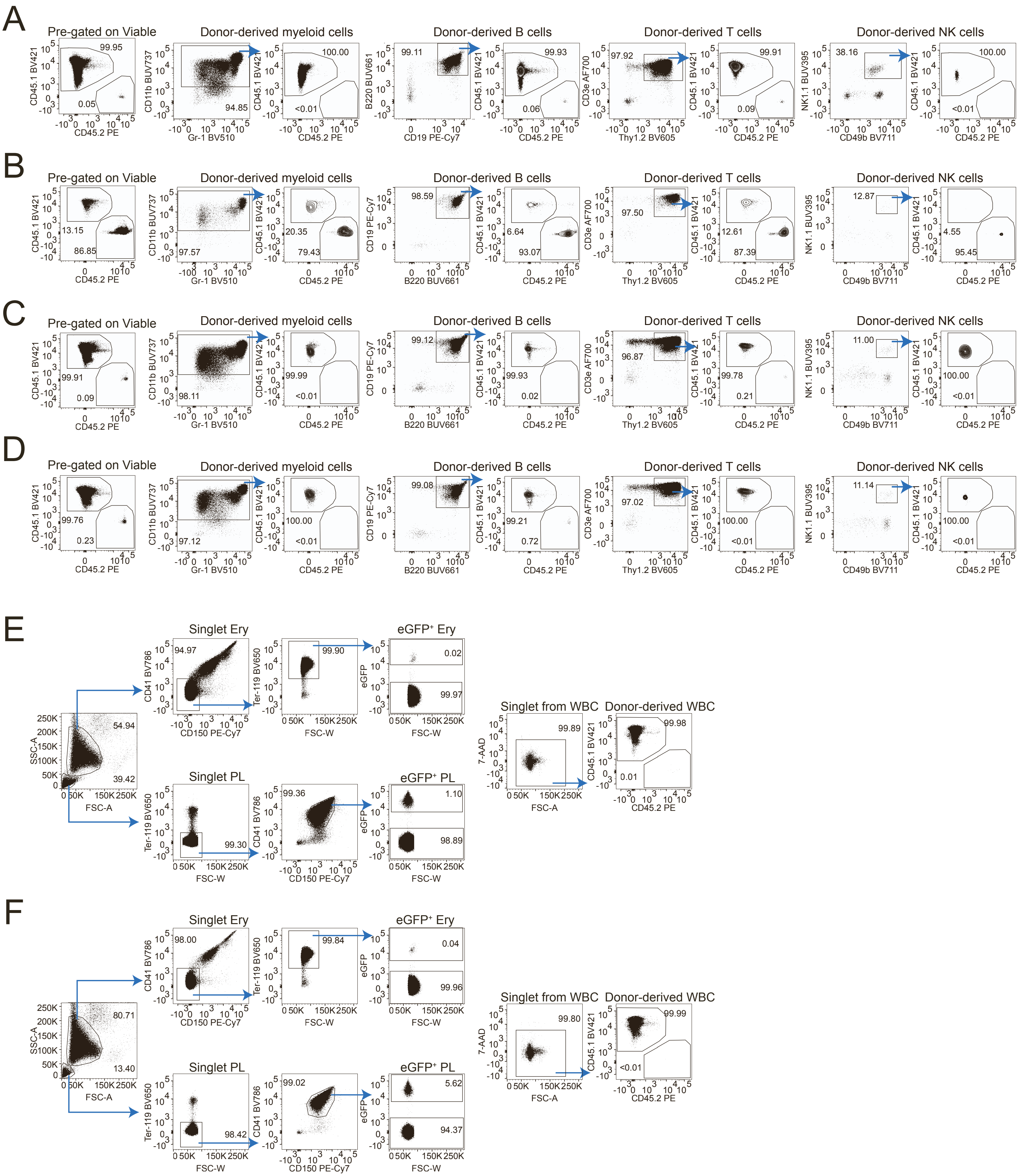

Figure S3, related to Figure 4

Examples of positively reconstituted mice in in single cell transplantation analyses

(A-D) Flow cytometry profiles of mature blood lineage analysis in peripheral blood showing representative single cell transplanted mice, defined as positively reconstituted but with different reconstitution profiles.

(A) A transplanted mouse with low reconstitution level to only B and T cells (see mouse ID = 014_013 in Figure S4B).

(B) A transplanted mouse with high reconstitution level to myeloid cells, B cells, T cells and NK cells (see mouse ID = 036_004 in Figure S4A).

(C) A transplanted mouse with low reconstitution level to only T cells (see mouse ID = 036_019 in Figure S4B).

(D) A transplanted mouse with low reconstitution level to only B cells, (see mouse ID = 036_044 in Figure S4B).

(E-F) Flow cytometry profiles of platelet-erythrocyte and white blood cell (WBC) analysis showing single cell transplanted mice, which were positively reconstituted for platelets and erythrocytes only. Mouse ID = 033_005 (E) and mouse ID = 033_037 (F) from Figure S4A are shown.

Total donor-derived cells were phenotypically defined as Ter-119^{-}CD41^{-}CD45.2^{+}, donor-derived B cells as Ter-119^{-}CD41^{-}CD11b^{-}Gr-1^{-}CD3e^{-}Thy1.2^{-}NK1.1^{-}CD49b^{-}B220^{+}CD19^{+} CD45.2^{+}, donor-derived NK cells as Ter-119^{-}CD41^{-}CD11b^{-}Gr-1^{-}B220^{-}CD19^{-}CD3e^{-}Thy1.2^{-} NK1.1^{+}CD49b^{+}CD45.2^{+}, donor-derived T cells as Ter-119^{-}CD41^{-}CD11b^{-}Gr-1^{-}B220^{-}CD19^{-} NK1.1^{-}CD49b^{-}CD3e^{+}Thy1.2^{+}CD45.2^{+} and donor-derived myeloid cells as Ter-119^{-}CD41^{-} B220^{-}CD19^{-}NK1.1^{-}CD49b^{-}CD3e^{-}Thy1.2^{-}CD11b^{+} CD45.2^{+}. Donor-derived platelets (PL) were phenotypically defined as Ter-119^{-}CD41^{+}CD150^{+} *Gata-1*-eGFP^{+} and erythrocytes (Ery) as CD41^{-}CD150^{-}Ter-119^{+}*Gata-1*-eGFP^{+}. Frequencies of parent gates are shown.

Figure S4, related to Figure 4 and 5

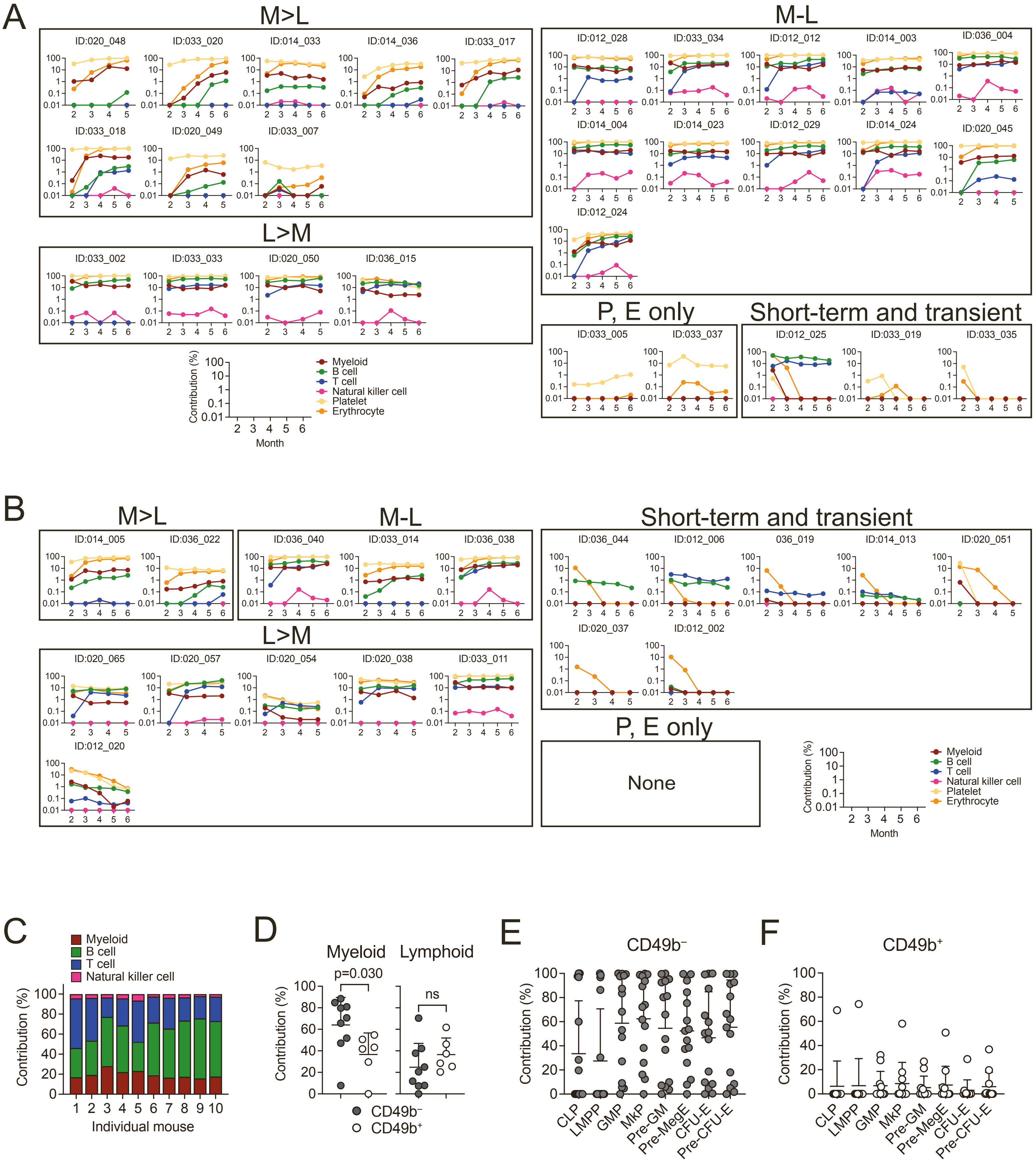

Figure S4, related to Figures 4 and 5

Repopulation profiles of single cell transplanted mice

(A-B) Repopulation patterns of single cell transplanted mice with LSKCD48^{-}CD34^{-}CD150^{hi} CD49b^{-} (CD49b^{-}) cells in (A) and LSKCD48^{-}CD34^{-}CD150^{hi}CD49b^{+} (CD49b^{+}) cells in (B), showing frequency of total donor contribution to myeloid, B cell, T cell, natural killer cell, platelet, and erythrocyte lineages in peripheral blood up to 6 months post-transplantation. Single cell transplanted mice were grouped according to their repopulation profiles, into myeloid reconstitution higher than lymphoid reconstitution (M>L), similar reconstitution levels of myeloid and lymphoid (M-L), lymphoid reconstitution higher than myeloid reconstitution (L>M), platelet and erythrocyte only reconstitution (P, E only) and short-term and transient reconstitution.

(C) Blood cell composition in the peripheral blood of 12-18-week-old unmanipulated mice (n = 10 mice from 7 experiments).

(D) Frequency of donor contribution to myeloid cells and lymphoid cells (B cells, T cells and NK cells) in the BM, 5-6 months following single cell transplantation of CD49b^{-} and CD49b^{+} cells ($n_{CD49b^{-}}$ = 9 mice and $n_{CD49b^{+}}$ = 6 mice, from 2 independent experiments).

(E-F) Frequency of donor contribution to progenitor cells, CLP, LMPP, GMP, MkP, PreGM, PreMegE, CFU-E and Pre-CFU-E in the BM, 5-6 months following single cell transplantation of CD49b^{-} (E) and CD49b^{+} (F) cells ($n_{CD49b^{-}}$ = 15 mice and $n_{CD49b^{+}}$ = 11 mice, from 3 independent experiments).

Data in (D-F) are presented as means ± SD. Frequencies of parent gates are shown in FACS plots. Statistical analysis was performed with a Mann-Whitney test in (D). ns = not significant.

Figure S5, related to Figure 4 and 5

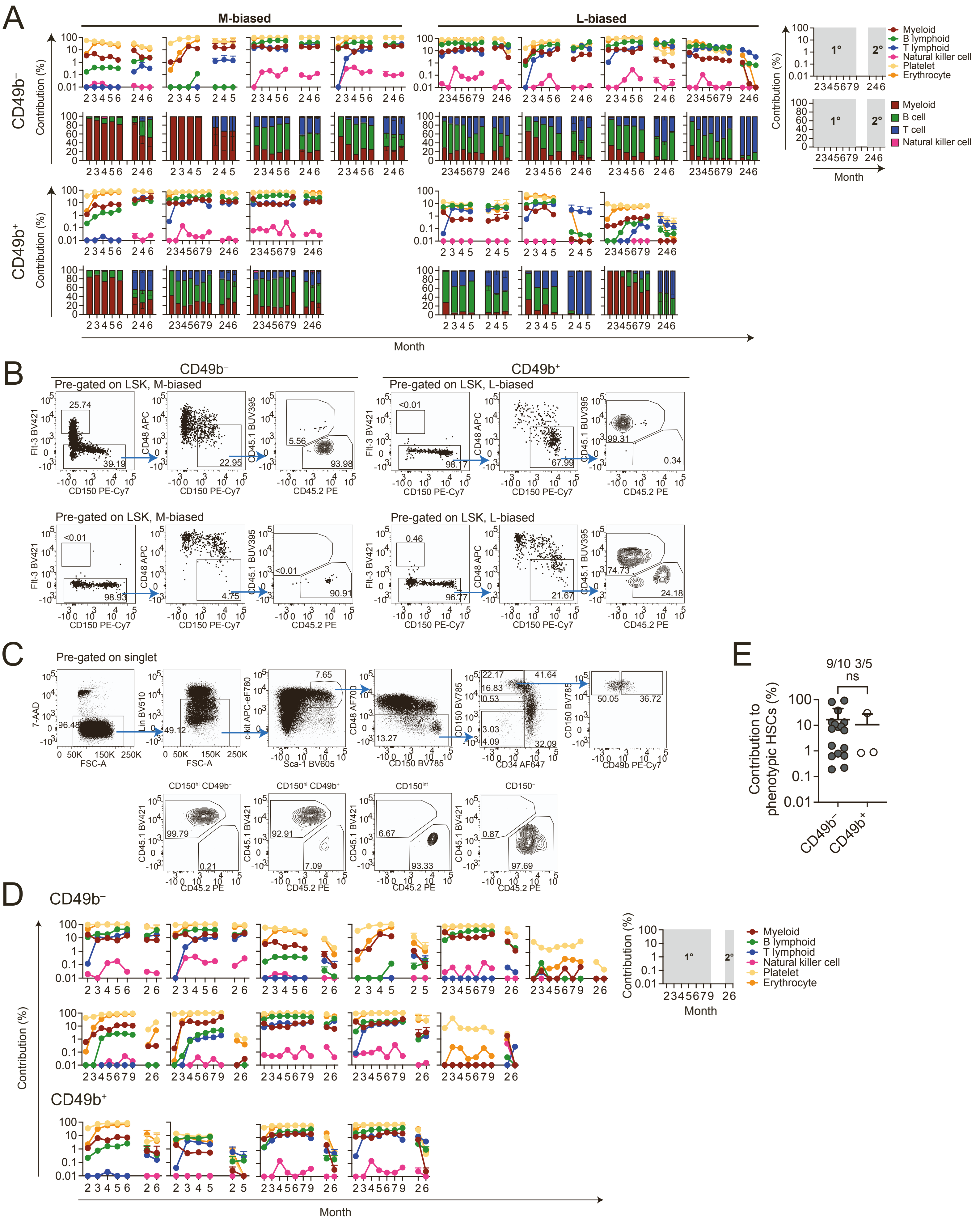

Figure S5, related to Figures 4 and 5

Repopulation of blood and bone marrow hematopoietic cells in primary and secondary single cell transplantations

(A) Frequency of total donor contribution to myeloid, B cell, T cell, natural killer cell, platelet and erythrocyte lineages in peripheral blood (top) and their corresponding relative contribution to M, B, T and NK within the donor leukocyte fraction (bottom), up to 5-9 months following single-cell transplantation of $CD49b^-$ and $CD49b^+$ populations, as well as secondary transplantation. Secondary transplantation was done with 10 million whole BM cells from the primary donor, transplanted into 1-5 secondary recipients per primary donor. Each graph represents data from one individual mouse in primary transplantation, with myeloid bias (M-biased) or lymphoid bias (L-biased) as indicated.

(B) Flow cytometry profiles of donor-derived phenotypic hematopoietic stem cells (HSC) in BM, from representative M-biased or L-biased secondary transplanted mice. Donor-derived phenotypic HSCs were defined as LSKFlt-3^-CD48^-CD150^+CD45.2^+. FACS plots were pre-gated on LSK. Frequencies of parent gates are shown. On average 2 million events were recorded per sample and ≥10 events were used to determine whether mice were positively reconstituted for HSCs.

(C) Flow cytometry profiles and gating strategy for phenotypic lineage-biased HSC subsets, from a representative c-Kit-enriched BM sample. Donor-derived HSC subsets were phenotypically defined as LSKCD48^-CD34^-CD150^{hi}CD$49b^-$CD45.2^+, LSKCD48^-CD34^-CD150^{hi}CD$49b^+$CD45.2^+, LSKCD48^-CD34^-CD150^{int}CD45.2^+ and LSKCD48^-CD34^-CD150^- CD45.2^+.

(D) Frequency of total donor contribution to myeloid, B cell, T cell, natural killer cell, platelet and erythrocyte lineages in peripheral blood, in single-cell transplantation of $CD49b^-$ and $CD49b^+$ populations, as well as secondary transplantation, up to 5-9 months post-transplantation. Secondary transplantation was done with 30-100 sorted donor-derived $CD49b^-$ or $CD49b^+$ cells per secondary recipient and 1-5 secondary recipients per primary donor. Each graph represents data from one individual mouse from primary transplantation.

(E) Donor contribution to phenotypic HSCs (LSKCD48^-CD150^+) in secondary transplantation of 30-100 sorted $CD49b^-$ and $CD49b^+$ cells, 5-6 months post-transplantation (n_{CD49b^-} = 17, n_{CD49b^+} = 3 mice, 5 independent experiments). The number of reconstituted primary donor mice out of all mice analyzed is indicated above the graph.

Data in (A, top), (D) and (E) are presented as means ± SD. Frequencies of parent gates are shown in FACS plots. Statistical analysis was performed with a Mann-Whitney test in (E). ns = not significant.

Figure S6, related to Figure 6

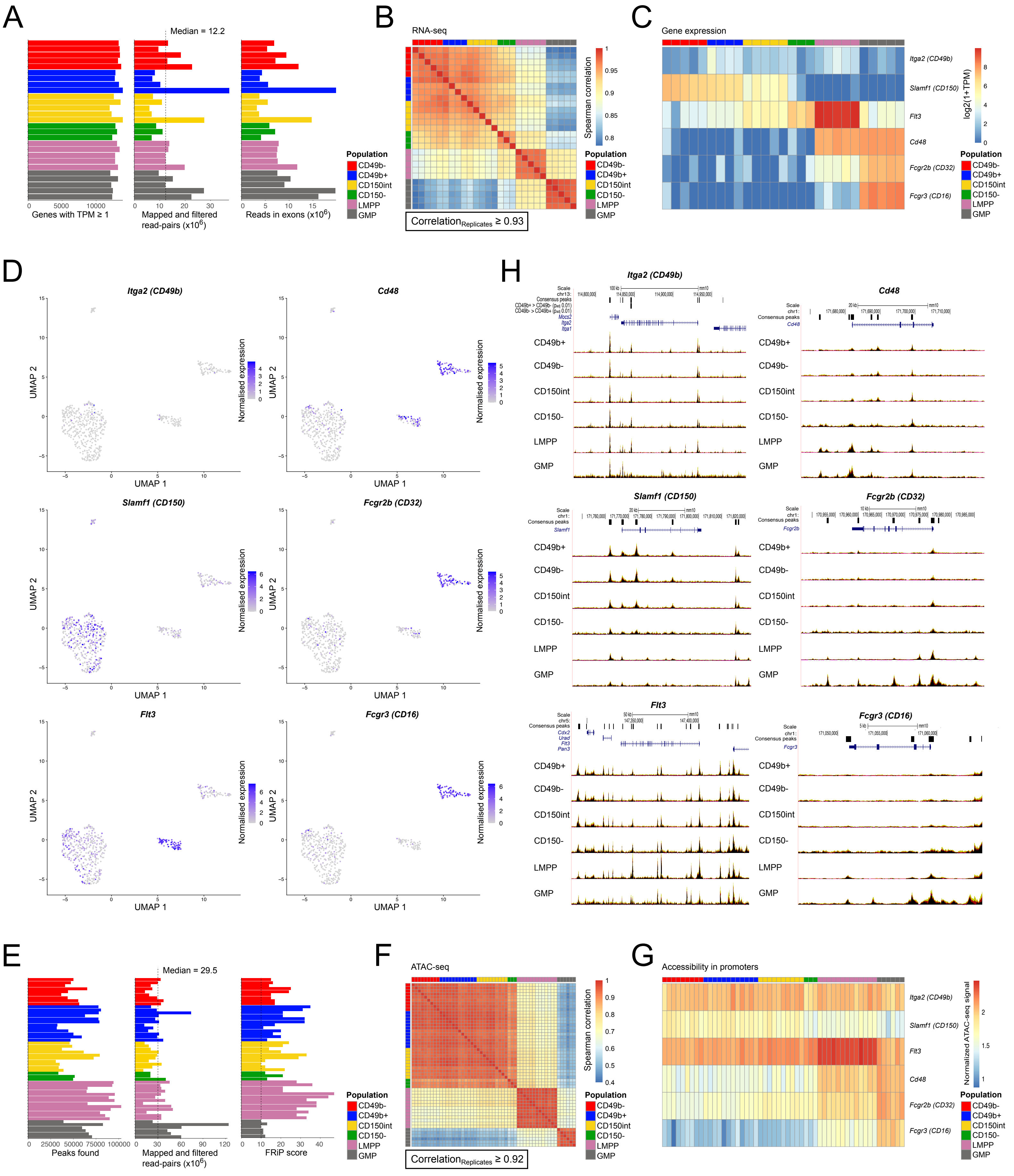

Figure S6, related to Figure 6

RNA- and ATAC-sequencing samples, and gene expression and chromatin accessibility of selected genes

(A) Distribution of number of expressed genes (≥1 TPM), number of mapped and filtered read-pairs and number of reads in exons among individual RNA-seq samples.

(B) Spearman correlation between RNA-seq samples. Expressed genes with ≥1 TPM in at least 3 samples are included. Correlation is calculated on $\log_{10}$ transformed and quantile normalized data.

(C) Heatmap of gene expression for selected, characteristic surface markers for the studied cell populations by bulk RNA-seq analysis.

(D) UMAP visualization of gene expression of selected, characteristic surface markers by single cell RNA-seq. The color intensity represents the expression level.

(E) Distribution of number of found consensus peaks, number of mapped and filtered read-pairs and fraction of reads in peaks (FRiP) score among individual ATAC-seq samples.

(F) Spearman correlation between ATAC-seq samples. Peaks with a normalized read count >1.5 in more than one third of any population are included. Correlation is calculated on $\log_{10}$ transformed and quantile normalized data.

(G) Heatmap of chromatin accessibility in the promoter (based on the GENCODE VM23 Comprehensive Transcript Set) of selected, characteristic surface markers for the studied cell populations. Signal corresponds to $\log_{10}$ transformed and quantile normalized read counts.

(H) UCSC browser tracks of ATAC-seq signal for selected, characteristic surface markers for the studied cell populations. Signal was scaled to 1 million mapped reads in peaks and for each cell population tracks of the median signal (± SD) are shown.

Figure S7, related to Figure 6 and 7

A

Gene name	Up in	log_2 fold change	adjusted p-value
Slc16a1	CD49b+	-2.09	0.002
Iqgap3	CD49b+	-1.91	0.007
Galnt6	CD49b+	-1.04	0.007
Mxd4	CD49b+	-2.36	0.020
6330416G13Rik	CD49b+	-3.38	0.046
Gfi1	CD49b-	3.21	0.025
Rrp12	CD49b-	3.12	0.037
Lrrc20	CD49b-	1.93	0.040

B

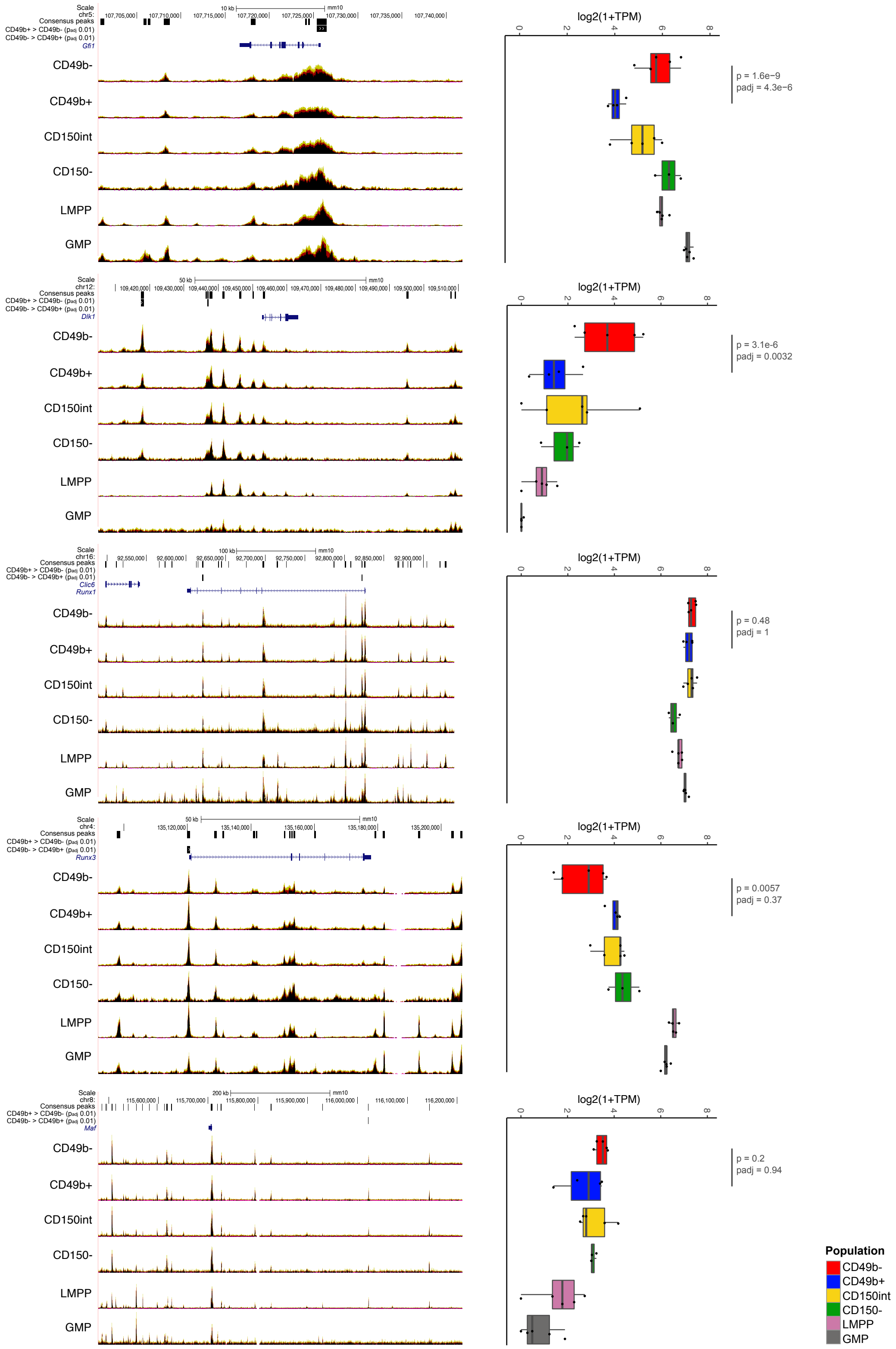

Figure S7, related to Figures 6 and 7

Differential gene expression and chromatin accessibility of select genes

(A) Differential expression analysis between $CD49b^-$ and $CD49b^+$ cells using the likelihood-ratio test for scRNA-seq data (logFC > 1, padj < 0.05).

(B) UCSC browser tracks of ATAC-seq signal for selected regions with differential accessibility between $CD49b^-$ and $CD49b^+$ cells (left) and RNA expression of the genes proximal to the differential regions (right). RNA expression in individual samples is shown as dots and boxplots show the distribution in each population.

Table S3. Antibody list, related to Figures 1-7 and Experimental procedures

ANTIBODIES	SOURCE	IDENTIFIER
Anti-mouse CD105 (Endoglin) BV650 (clone MJ7/18)	BD Biosciences	Cat#: 740609
Anti-mouse CD117 (c-kit) APC-eF780 (clone 2B8)	ThermoFisher Scientific	Cat#: 47-1171-82
Anti-mouse CD11b (Mac-1) BUV395 (clone M1/70)	BD Biosciences	Cat#: 563553
Anti-mouse CD11b (Mac-1) BUV737 (clone M1/70)	BD Biosciences	Cat#: 564443
Anti-mouse/Human CD11b (Mac-1) BV510 (clone M1/70)	BioLegend	Cat#: 101263
Anti-mouse CD11b (Mac-1) eF450 (clone M1/70)	ThermoFisher Scientific	Cat#: 48-0112-82
Anti-mouse CD11b (Mac-1) PE (clone M1/70)	BD Biosciences	Cat#: 553311
Anti-mouse CD127 (IL7-Ra) BV711 (clone A7R34)	BioLegend	Cat#: 135035
Anti-mouse CD135 (Flt-3) PE (clone A2F10)	BioLegend	Cat#: 135306
Anti-mouse CD135 (Flt-3) BV421 (clone A2F10.1)	BD Biosciences	Cat#: 562898
Anti-mouse CD150 (SLAM) BV785 (clone TC15-12F12.2)	BioLegend	Cat#: 115937
Anti-mouse CD150 (SLAM) PE (clone TC15-12F12.2)	BioLegend	Cat#: 115904
Anti-mouse CD150 (SLAM) PE-Cy7 (clone TC15-12F12.2)	BioLegend	Cat#: 115914
Anti-mouse CD16/32 AF700 (clone 93)	ThermoFisher Scientific	Cat#: 56-0161-82
Anti-mouse CD16/32 (Fc-block) Purified (clone 2.4G2)	BD Biosciences	Cat#: 553142
Anti-mouse CD19 PE-Cy7 (clone 6D5)	BioLegend	Cat#: 115520
Anti-mouse CD19 PerCP-Cy5.5 (clone 1D3)	ThermoFisher Scientific	Cat#: 45-0193-82
Anti-mouse CD201 (EPCR) PE (clone eBio1560)	ThermoFisher Scientific	Cat#: 12201282
Anti-mouse CD202b (Tie-2) Biotin (clone TEK4)	BioLegend	Cat#: 124006
Anti-mouse CD229 (Ly9) APC (clone Ly9ab3)	BioLegend	Cat#: 122907
Anti-mouse CD244.2 (2B4) PE (clone eBio244F4)	ThermoFisher Scientific	Cat#: 12-2441-82
Anti-mouse CD34 FITC (clone RAM34)	ThermoFisher Scientific	Cat#: 11-0341-85
Anti-mouse CD34 AF647 (clone RAM34)	BD Biosciences	Cat#: 560230
Anti-mouse CD3e AF700 (clone 500A2)	BD Biosciences	Cat#: 557984
Anti-mouse CD3e BV510 (clone 145-2C11)	BioLegend	Cat#: 100353
Anti-mouse CD3e BUV395 (clone 145-2C11)	BD Biosciences	Cat#: 563565
Anti-mouse CD3e eF450 (clone 145-2C11)	ThermoFisher Scientific	Cat#: 48-0031-82
Anti-mouse CD4 BUV395 (clone RM4-5)	BD Biosciences	Cat#: 740208
Anti-mouse CD41 BV786 (clone MWReg30)	BD Biosciences	Cat#: 740903
Anti-mouse CD41 PE (clone eBioMWReg30)	ThermoFisher Scientific	Cat#: 12-0411-83
Anti-mouse CD45.1 (Ly5.1) BV421 (clone A20)	BD Biosciences	Cat#: 563983
Anti-mouse CD45.1 (Ly5.1) BUV395 (clone A20)	BD Biosciences	Cat#: 565212
Anti-mouse CD45.2 (Ly5.2) PE (clone 104)	BioLegend	Cat#: 109808
Anti-mouse CD45.2 (Ly5.2) APC-Fire750 (clone 104)	BioLegend	Cat#: 109852
Anti-mouse CD45R/B220 BUV395 (clone RA3-6B2)	BD Biosciences	Cat#: 563793
Anti-mouse CD45R/B220 BUV661 (clone RA3-6B2)	BD Biosciences	Cat#: 565077
Anti-mouse CD45R/B220 eF450 (clone RA3-6B2)	ThermoFisher Scientific	Cat#: 48-0452-82
Anti-mouse CD45R/B220 FITC (clone RA3-6B2)	BD Biosciences	Cat#: 553088
Anti-mouse/Human CD45R/B220 PE-Dazzle 594 (clone RA3-6B2)	BioLegend	Cat#: 103258

Anti-mouse/Human CD45R/B220 BV510 (clone RA3-6B2)	BioLegend	Cat#: 103248
Anti-mouse CD48 AF700 (clone HM48-1)	BioLegend	Cat#: 103426
Anti-mouse CD48 APC (clone HM48-1)	BioLegend	Cat#: 103412
Anti-mouse CD48 PerCP-Cy5.5 (clone HM48-1)	BioLegend	Cat#: 103422
Anti-mouse CD49b PE-Cy7 (clone HMα2)	BioLegend	Cat#: 103518
Anti-mouse CD49b AF647 (clone HMα2)	BioLegend	Cat#: 103511
Anti-mouse CD49b Biotin (clone HMa2)	ThermoFisher Scientific	Cat#: 13-0491-85
Anti-mouse CD49b BV711 (clone HMα2)	BD Biosciences	Cat#: 740704
Anti-mouse CD5 BV510 (clone 53-7.3)	BioLegend	Cat#: 100627
Anti-mouse CD5 BUV395 (clone 53-7.3)	BD Biosciences	Cat#: 740206
Anti-mouse CD5 eF450 (clone 53-7.3)	ThermoFisher Scientific	Cat#: 48-0051-82
Anti-mouse CD61 Biotin (clone 2C9.G2)	BD Biosciences	Cat#: 553345
Anti-mouse CD86 Biotin (clone GL-1)	BioLegend	Cat#: 105003
Anti-mouse CD8a BUV395 (clone 53-6.7)	BD Biosciences	Cat#: 563786
Anti-mouse CD9 Biotin (clone MZ3)	BioLegend	Cat#: 124803
Anti-mouse Esam APC (clone 1G8/ESAM)	BioLegend	Cat#: 136207
Anti-mouse F4/80 APC (clone BM8)	BioLegend	Cat#: 123116
Anti-mouse F4/80 APC (clone BM8)	ThermoFisher Scientific	Cat#: 17-4801-82
Anti-mouse Gr-1 BV510 (clone RB6-8C5)	BioLegend	Cat#: 108437
Anti-mouse Gr-1 BUV395 (clone RB6-8C5)	BD Biosciences	Cat#: 563849
Anti-mouse Gr-1 (Ly-6G and Ly-6C) APC (clone RB6-8C5)	BioLegend	Cat#: 108412
Anti-mouse Gr-1 (Ly-6G) eF450 (clone RB6-8C5)	ThermoFisher Scientific	Cat#: 48-5931-82
Anti-mouse Gr-1 (Ly-6G/Ly-6C) PE (clone RB6-8C5)	BD Biosciences	Cat#: 553128
Anti-mouse NK1.1 BUV395 (clone PK136)	BD Biosciences	Cat#: 564144
Anti-mouse NK1.1 FITC (clone PK136)	ThermoFisher Scientific	Cat#: 11-5941-82
Anti-mouse Sca-1 (Ly-6A/E) BV605 (clone D7)	BioLegend	Cat#: 108134
Anti-mouse Sca-1 (Ly-6A/E) AF700 (clone D7)	ThermoFisher Scientific	Cat#: 56-5981-82
Anti-mouse Sca-1 (Ly-6A/E) PE-Cy7 (clone E13-161.7)	BioLegend	Cat#: 122514
Anti-mouse Ter-119 BV510 (clone TER-119)	BD Biosciences	Cat#: 563995
Anti-mouse Ter-119 BV650 (clone TER-119)	BD Biosciences	Cat#: 747739
Anti-mouse Ter-119 BUV395 (clone TER-119)	BD Biosciences	Cat#: 563827
Anti-mouse Ter-119 eF450 (clone TER-119)	ThermoFisher Scientific	Cat#: 48-5921-82
Anti-mouse Thy1.2 BV605 (clone 53-2.1)	BioLegend	Cat#: 140318
Anti-mouse Streptavidin BV570	BioLegend	Cat#: 405227
Anti-mouse Streptavidin PE-TexasRed	BD Biosciences	Cat#: 551487
7-AAD	BD Biosciences	Cat#: 559925
DAPI	ThermoFisher Scientific	Cat#: D3571
Sytox Blue	ThermoFisher Scientific	Cat#: S34857
Ki-67 FITC	BD Biosciences	Cat#: 556026
BrdU PE	BD Biosciences	Cat#: 556029

Table S4. Immunophenotype of hematopoietic populations, related to Figures 1-7

**OP9 readout analysis**	
B cells	$CD19^{+}B220^{+}$
Myeloid cells	$CD11b^{+}Gr\text{-}1^{+}$ and/or $CD11b^{+}F4/80^{+}$
**Peripheral blood analysis**	
B cells	$CD41^{-}$ $Ter119^{-}$ $CD3e^{-}$ $Thy1.2^{-}$ $NK1.1^{-}$ $CD49b^{-}$ $CD11b^{-}$ $Gr\text{-}1^{-}$ **$B220^{+}$ $CD19^{+}$**
Myeloid cells	$CD41^{-}$ $Ter119^{-}$ $CD3e^{-}$ $Thy1.2^{-}$ $NK1.1^{-}$ $CD49b^{-}$ $B220^{-}$ $CD19^{-}$ **$CD11b^{+}$**
T cells	$CD41^{-}$ $Ter119^{-}$ $NK1.1^{-}$ $CD49b^{-}$ $CD11b^{-}$ $Gr\text{-}1^{-}$ $B220^{-}$ $CD19^{-}$ **$CD3e^{+}$ $Thy1.2^{+}$**
NK cells	$CD41^{-}$ $Ter119^{-}$ $B220^{-}$ $CD19^{-}$ $CD11b^{-}$ $Gr\text{-}1^{-}$ $CD3e^{-}$ $Thy1.2^{-}$ **$NK1.1^{+}$ $CD49b^{+}$**
Platelets	$Ter119^{-}$ **$CD41^{+}$ $CD150^{+}$**
Erythrocytes	$CD41^{-}$ $CD150^{-}$ **$Ter\text{-}119^{+}$**
**Bone marrow stem- and progenitor analysis**	
Lymphoid-primed multipotent progenitor (LMPP)	Lin^{-} $Sca\text{-}1^{+}$ $c\text{-}kit^{+}$ (LSK) $Flt3^{hi}$
Common lymphoid progenitor (CLP)	Lin^{-} $B220^{low}$ $Sca\text{-}1^{low}$ $c\text{-}kit^{low}$ $Flt\text{-}3^{hi}$ $IL\text{-}7Ra^{+}$
Megakaryocyte progenitor (MkP)	Lin^{-} $Sca\text{-}1^{-}$ $c\text{-}kit^{+}$ (LK) $CD150^{+}$ $CD41^{+}$
Granulocyte-monocyte progenitor (GMP)	LK $CD41^{-}$ $CD150^{-}$ $CD16/32^{+}$
Pre-granulocyte-monocyte (Pre-GM)	LK $CD41^{-}$ $CD16/32^{-}$ $CD150^{-}$ $CD105^{-}$
Pre-megakaryocyte-erythrocyte (Pre-MegE)	LK $CD41^{-}$ $CD16/32^{-}$ $CD150^{+}$ $CD105^{-}$
Pre-colony forming unit erythroid (Pre-CFU-E)	LK $CD41^{-}$ $CD16/32^{-}$ $CD150^{+}$ $CD105^{+}$
Colony forming unit erythroid (CFU-E)	LK $CD41^{-}$ $CD16/32^{-}$ $CD150^{-}$ $CD105^{+}$
Hematopoietic stem cell (HSC)	LSK $Flt\text{-}3^{-}$ $CD48^{-}$ $CD150^{+}$ or LSK $CD48^{-}$ $CD150^{+}$
**Hematopoietic stem cell analysis**	
$CD49b^{-}$ subset	LSK $CD48^{-/low}$ $CD34^{-/low}$ $CD150^{hi}$ $CD49b^{-}$
$CD49b^{+}$ subset	LSK $CD48^{-/low}$ $CD34^{-/low}$ $CD150^{hi}$ $CD49b^{+}$
$CD150^{hi}$ subset	LSK $CD48^{-/low}$ $CD34^{-/low}$ $CD150^{hi}$
$CD150^{int}$ subset	LSK $CD48^{-/low}$ $CD34^{-/low}$ $CD150^{int}$
$CD150^{-}$ subset	LSK $CD48^{-/low}$ $CD34^{-/low}$ $CD150^{-}$

Table S5. Cell culture conditions for in vitro assays, related to Figures 1-2

**Megakaryocyte liquid culture assay**	**Company**	**Catalogue number**	**Final concentration**
x-vivo15 with Gentamicin and L-glutamine	Lonza	BE02-060F	
BIT 9500	Stem Cell Technologies	09500	20% v/v
Fetal bovine serum (FBS)	Cytiva Hyclone	SH30071	10% v/v
β-mercaptoethanol	Merck	M7154-100ML	0.1mM
rmSCF	R&D Systems	455-MC	25 ng/ml
rmFlt3-L	Peprotech	250-31L	25 ng/ml
rmTpo	R&D Systems	488-TO	25 ng/ml
rmIL-3	R&D Systems	403-ML	10 ng/ml
**Cell division assay**	**Company**	**Catalogue number**	**Final concentration**
x-vivo15 with Gentamicin and L-glutamine	Lonza	BE02-060F	
Fetal bovine serum (FBS)	Cytiva Hyclone	SH30071	10% v/v
β-mercaptoethanol	Merck	M7154-100ML	0.1mM
rmSCF	Peprotech	250-03	50 ng/ml
rhFlt3-L	Peprotech	300-19	50 ng/ml
rhTpo	Peprotech	300-18	50 ng/ml
rmIL-3	Peprotech	213-13	20 ng/ml
**OP9 co-culture assay**	**Company**	**Catalogue number**	**Final concentration**
Opti-MEM with GlutaMAX	Gibco	51985-026	
Fetal bovine serum (FBS)	Cytiva Hyclone	SH30071	10% v/v
β-mercaptoethanol	Merck	M7154-100ML	0.1mM
Penicillin-Streptomycin	Gibco	15140122	1% v/v
rmSCF	R&D Systems or Peprotech	455-MC or 250-03	25 ng/ml
rmFlt3-L or rhFlt3-L	Peprotech	250-31L or 300-19	25 ng/ml
rmIL-7 or rhIL-7	R&D Systems or Peprotech	407-ML or 200-07	20 ng/ml

SUPPLEMENTAL EXPERIMENTAL PROCEDURES

Experimental animals

Animals were bred and maintained at the Preclinical Laboratory, Karolinska University Hospital and all experiments were approved by the regional ethical committee, Linköping ethical committee (ethical number 882). Females and males between 8-17 weeks old were used and were on a C57BL/6J background. B6.SJL-*Ptprc*^{a}*Pepc*^{b}/BoyCrl and B6.SJL-*Ptprc*^{a}*Pepc*^{b}/BoyJ mice (CD45.1) were used as primary and secondary recipients in transplantation experiments. Gata-1 eGFP[1] mice (CD45.2) were backcrossed >8 generations to a C57BL/6J background and were used as donor mice in transplantation experiments.

Hematopoietic cell preparation and staining

Bone marrow (BM) cell suspensions were prepared by crushing forelimbs, hindlimbs and hip bones into Phosphate-Buffered Saline (PBS, Gibco) with 5% FCS (Gibco) and 2 mM Ethylenediaminetetraacetic acid (EDTA, Merck).

Unfractionated BM cells were either Fc-blocked with purified CD16/32 (BD Biosciences) or pre-stained with fluorophore-conjugated CD16/32 antibody and subsequently stained with antibodies against cell surface marker antigens (Table S3). For detection of hematopoietic stem cells (HSCs), unfractionated BM cells were enriched using CD117 MicroBeads (Miltenyi Biotec) followed by immunomagnetic separation of the cells and subsequently stained with antibodies against cell-surface markers. Immunophenotype definitions of hematopoietic populations are described in Table S4.

Peripheral blood (PB) was collected from the tail vein into lithium heparin coated microvette tubes (Sarstedt) followed by platelet and erythrocyte isolation as performed in Carrelha *et al*[2]. Leukocytes were subsequently isolated from PB samples and stained with antibodies against cell surface antigens as described previously[3].

Flow cytometry experiments

Cell sorting experiments were performed using FACSAria™ Fusion or BD FACSAria™ III cell sorters (BD Biosciences) with a mean cell sorting purity of 95.3%±2.3%. Flow cytometry analysis was performed on LSR Fortessa™ or FACSymphony™ A5. Fluorescence minus one (FMO) controls were included in all flow cytometry experiments. Gates were set using FMO controls, backgating of the populations of interest or using internal controls (known negative and positive populations for the markers). Post-acquisition data analyses were done using the FlowJo software version 10 (BD Biosciences).

In single cell transplantation and single cell *in vitro* differentiation experiments, verification of single cell deposition into 96-well or 60-well plates was performed by sorting fluorescent beads.

Transplantation experiments

In all transplantation experiments, single cells or 5 cells were intravenously injected into lethally irradiated CD45.1 mice. The full irradiation dose was given as two split doses (2 x 600cGy). Donor and recipient mice were sex matched. Single cell transplantation experiments were performed as described in Carrelha *et al*[2]. In primary transplantations, single or five HSCs were transplanted with 200,000-250,000 BM support cells (CD45.1). Secondary transplantations were performed with reconstituted primary recipient mice using 10×10^{6} unfractionated BM or 30-100 sorted CD49b^{-} or CD49b^{+} HSCs with 200,000 BM support cells. Cells from one primary donor mouse were transplanted into 1-5 lethally irradiated secondary recipients. Peripheral blood analyses from transplanted mice were periodically performed between 2- and 6-months post-transplantation and up to 9 months in extended long-term experiments.

Reconstitution threshold levels and lineage bias

The total donor reconstitution level was calculated based on the frequency of CD45.2^{+} events in total white blood cells (CD45.1 + CD45.2 cells). The transplanted mice were considered reconstituted when the total donor contribution in white blood cells (CD45.2^{+}) or in platelets (Gata-1 eGFP^{+}) in the peripheral blood was $\geq$0.1% and represented by $\geq$10 events in the donor gate. The single cell transplantation efficiency was calculated based on the number of reconstituted mice at 2 months post-transplantation and the total number of mice that was transplanted. Mice that were found dead or sacrificed due to animal welfare reasons before 2 months post-transplantation were excluded from the total number of mice that was transplanted.

The reconstitution of B, T, NK and myeloid cells was calculated based on the frequency of CD45.2^{+} events within each blood cell lineage. The reconstitution of platelets and erythrocytes was calculated based

on the frequency of eGFP$^+$ cells within the blood lineages (Figure S2). Mice were considered positive for specific blood cell lineages when donor lineage reconstitution was ≥0.01% and represented by ≥10 events in the donor gate. Long-term (LT; ≥5-6 months) or short-term (ST; <5-6 months) repopulating activity was based on the presence or absence of myeloid or platelet and erythrocyte repopulation in the peripheral blood (≥0.01% CD45.2$^+$ CD11b$^+$ or eGFP$^+$CD41$^+$CD150$^+$ and eGFP$^+$Ter-119$^+$), 5-6 months following transplantation.

Relative donor reconstitution levels were calculated based on the frequency of B, T, NK and myeloid cells within CD45.2$^+$ cells. Lineage bias from single cell transplanted mice was categorized according to the ratio of lymphoid (L; including B, T and NK cells) to myeloid (M) cell contribution (L/M) in the peripheral blood at 5-6 months post-transplantation. A lineage-balanced pattern has a L/M ratio of 6.0±2.0[4]. Lineage-bias was therefore classified as myeloid-biased (L/M <4), lymphoid-biased (L/M >8) or lineage-balanced (L/M ≥4 and ≥8).

In HSC repopulation analyses on average 2 million events were recorded per sample and ≥10 events were used to determine whether mice were positively reconstituted for HSCs (Figures S2C and S5B).

In vitro assays

To assess myeloid and B cell lineage potentials of HSC subsets *in vitro,* OP9 co-culture experiments were performed with the OP9 cell line as described previously[5], and assessed after 3 weeks of culture.

Megakaryocyte and erythrocyte potentials of HSC subsets were evaluated as previously described[5]. Briefly, megakaryocyte potential was evaluated by manually plating 1 cell/well into 60-well MicroWell MiniTrays (Nunc, ThermoScientific) and assessed after 13 days of culture by an inverted microscope for the presence or absence of megakaryocytic cells in the cultures. Erythroid potential was evaluated by plating 30 HSCs in complete methylcellulose (GF M3434; StemCell Technologies). Cultures were evaluated for erythroid colonies after 12 days with 2,7-diaminofluorene staining (Merck) as previously described[5].

Cell division kinetics of single cell sorted HSC subsets were performed as previously described[3]. Briefly, single cells were sorted directly into 60-well MicroWell MiniTrays. The number of cells and their cell divisions in the wells were regularly scored using an inverted microscope at 24-, 48-, 72- and 96-hours post-sorting.

Cell culture conditions for different *in vitro* assays are described in Table S5.

Cell cycle and cell proliferation assays

Cell cycle analysis by Ki-67 staining was performed with the BD Cytofix/Cytoperm Kit (BD Biosciences). Cell proliferation analysis was performed by the 5-Bromo-2'-deoxyuridine (BrdU) incorporation assay[3] with one dose of intraperitoneal injection of BrdU (50ug/g bodyweight), followed by administration of BrdU (Merck) in the drinking water (800ug/ml) for three days following the intraperitoneal injection. BrdU visualization was performed with the BD BrdU Flow Kit (BD Biosciences) according to the manufacturer's instructions.

RNA-sequencing

For RNA-sequencing, 250 or 500 cells from CD45.1 mice were FACS-sorted into 5 or 10 ul of Single cell lysis solution containing DNAse I (Single cell lysis kit, Invitrogen/ThermoFisher Scientific) and after 15 minutes incubation the reaction was stopped by adding stop solution according to the manufacturer's protocol. cDNA synthesis for strand specific RNA-sequencing libraries was done on RNA from 250-500 cells using NEBNext Ultra II RNA First Strand Synthesis (New England BioLabs) and NEBNext Ultra II Directional RNA Second Strand Synthesis (New England BioLabs) modules, in combination with QIAseq FastSelect rRNA HMR kit (Qiagen) for rRNA block. Custom made Tn5 (transposase) and replacement index primers were used for library preparation. Libraries were pooled and paired-end sequenced (2 x 41 cycles) using the NextSeq 500 system (Illumina, San Diego, CA). Paired-end reads were mapped to the mm10 reference genome using STAR (v.2.5.2b)[6]. Data from technical replicates, derived from the same biological sample, were merged when available. Quantification of reads in exons was done using HOMER[7]. Data was normalized for sequencing depth by converting to transcripts per million (TPM) in R. Genes with ≥1 TPM in ≥3 samples were considered expressed and used for analysis. For PCA analysis and visualization of gene expression in heatmaps and boxplots $\log_2$(TPM+1) values were used. Correlation between samples was calculated using $\log_{10}$ transformed and quantile normalized data. Differential

expression analysis was done using edgeR[8], basing statistical significance on the absolute fold change and Benjamini-Hochberg adjusted p-value (p_{adj}) from edgeR's exact test.

Single-cell cDNA libraries were prepared according to the previously described Smart Seq2 protocol[9]. In brief, mRNA was transcribed into cDNA using oligo(dT) primer and SuperScript II reverse transcriptase (ThermoFisher Scientific). Second strand cDNA was synthetized using a template switching oligo. The synthetized cDNA was then amplified by polymerase chain reaction (PCR) for 25–26 cycles. Purified cDNA was quality controlled (QC) by fragment analysis on a 2100 Bioanalyzer with a DNA High Sensitivity chip (Agilent Biotechnologies). When the sample passed the QC, the cDNA was fragmented and tagged (tagmented) using Tn5 transposase, and each single well was uniquely indexed using the Illumina Nextera XT index kits (Set A–D). Thereafter, the uniquely indexed cDNA libraries from one 384-well plate were pooled into one sample to be sequenced on one lane of a HiSeq3000 sequencer (Illumina), using dual indexing and single 50 base-pair reads. Reads were demultiplexed, aligned to the mm10 reference genome using Tophat2 and deduplicated using samtools. Subsequent analysis was done in R using the Seurat package (v.4.1.0). Cells with an RNA count < 50,000 or >750,000 reads, with >10% mitochondrial reads, or with >10% contribution from the ERCC spike-in controls were excluded from analysis. Genes were filtered to have a read count >150 across all included cells. Data was then normalized using Seurat´s LogNormalize method with a scale factor of 500,000. PCA was run on the 2,000 most variable genes and the top 10 principal components were used as input to create UMAP plots. The likelihood-ratio test was used for differential analysis.

ATAC-sequencing

ATAC-seq was performed using the Omni-ATAC protocol with modifications[10]. Five hundred cells from CD45.1 mice were FACS sorted into tubes containing 12.5ul 2x TD buffer (Illumina), 5.75ul PBS and 2.5ul of water. After sorting 0.25µl each of digitonin and TWEEN 20 (Merck) was added, followed by 1.25ul of Tn5 transposase (Illumina). Samples were then incubated for 30 minutes at 37°C with agitation at 300 rpm and purified using a MinElute PCR Purification Kit (Qiagen). Library fragments were amplified using 1xNEBnext PCR master mix (New England BioLabs) and 0.1 µM of custom PCR primers. Libraries were pooled and paired-end sequenced (2 x 41 cycles) using the NextSeq 550 system (Illumina, San Diego, CA). The nf-core ATAC-sequencing pipeline (v.1.0.0)[11] was used to align reads to the mm10 reference genome, filter aligned reads and for quality control. Data from technical replicates, derived from the same biological sample, was merged when available. For visualization, read coverage was normalized to 10^6 mapped reads in peaks and tracks with the median read coverage ± standard deviation was created for each population to allow for visualization using the UCSC genome browser[12]. Peak calling, quantification of reads in consensus peaks and peak annotation were done using HOMER[7], with options "-region -size 100 -minDist 200" for peak calling. Read counts were $\log_{10}$ transformed and quantile normalized in R. Peaks were considered to be found in a population if they had a normalized read count >1.5 in more than one third of the samples. Peaks were filtered to exclude those not found in any analyzed population. For specific analysis of the CD150$^+$ populations, peaks not found in any CD150$^+$ population were additionally excluded. Log_{10} transformed and quantile normalized data was used for PCA analysis, visualization of accessibility in heatmaps and calculation of correlation between samples. DESeq2[13] was used for differential analysis on the filtered peak sets, basing statistical significance on the absolute fold change and Benjamini-Hochberg adjusted p-value (p_{adj}) from the nbinomWaldTest. Enriched binding motifs in peaks with increased accessibility were identified with the HOMER program findMotifGenome.pl using a custom background of peaks with decreased accessibility in the respective population. Motifs found in less than 10% of peaks were excluded. Gene ontology analysis GREAT[14] (v.4.0.4) was used with online default settings. For footprinting analysis, the aligned and filtered samples of each population were down sampled to the same depth and merged, and the TOBIAS framework[15] was used for all further analysis with default settings and the JASPAR 2020 CORE vertebrate collection as TF motif database[16].

Statistical methods

Statistical analysis not explicitly described above was done in GraphPad Prism v.9.2.0 for Mac OS. Non-parametric tests were performed using the Mann-Whitney test or the Kruskal-Wallis with Dunn's multiple comparison test. Generally, at least 3 biological replicates were included in each test group, and the mean ± SD are shown. A biological replicate constitutes cells from one mouse or from a pool of mice. Statistically significant differences are indicated in all figures with exact P-values, unless stated otherwise.

Supplemental references

1. Drissen R, Buza-Vidas N, Woll P, Thongjuea S, Gambardella A, Giustacchini A, et al. Distinct myeloid progenitor differentiation pathways identified through single cell RNA sequencing. Nat Immunol. 2016 Jun;17(6):666–76.

2. Carrelha J, Meng Y, Kettyle LM, Luis TC, Norfo R, Alcolea V, et al. Hierarchically related lineage-restricted fates of multipotent haematopoietic stem cells. Nature. 2018 Feb 1;554(7690):106–11.

3. Luc S, Huang J, McEldoon JL, Somuncular E, Li D, Rhodes C, et al. Bcl11a Deficiency Leads to Hematopoietic Stem Cell Defects with an Aging-like Phenotype. Cell Reports. 2016 Sep 20;16(12):3181–94.

4. Müller-Sieburg CE, Cho RH, Thoman M, Adkins B, Sieburg HB. Deterministic regulation of hematopoietic stem cell self-renewal and differentiation. Blood. 2002 Aug 15;100(4):1302–9.

5. Luc S, Luis TC, Boukarabila H, Macaulay IC, Buza-Vidas N, Bouriez-Jones T, et al. The Earliest Thymic T Cell Progenitors Sustain B Cell and Myeloid Lineage Potentials. Nat Immunol. 2012 Apr;13(4):412–9.

6. Dobin A, Davis CA, Schlesinger F, Drenkow J, Zaleski C, Jha S, et al. STAR: ultrafast universal RNA-seq aligner. Bioinformatics. 2013;29(1):15–21.

7. Heinz S, Benner C, Spann N, Bertolino E, Lin YC, Laslo P, et al. Simple Combinations of Lineage-Determining Transcription Factors Prime cis-Regulatory Elements Required for Macrophage and B Cell Identities. Mol Cell. 2010;38(4):576–89.

8. Robinson MD, McCarthy DJ, Smyth GK. edgeR: a Bioconductor package for differential expression analysis of digital gene expression data. Bioinformatics. 2010;26(1):139–40.

9. Picelli S, Björklund ÅK, Faridani OR, Sagasser S, Winberg G, Sandberg R. Smart-seq2 for sensitive full-length transcriptome profiling in single cells. Nat Methods. 2013;10(11):1096–8.

10. Corces MR, Trevino AE, Hamilton EG, Greenside PG, Sinnott-Armstrong NA, Vesuna S, et al. An improved ATAC-seq protocol reduces background and enables interrogation of frozen tissues. Nat Methods. 2017 Aug 28;14(10):959–62.

11. Ewels PA, Peltzer A, Fillinger S, Patel H, Alneberg J, Wilm A, et al. The nf-core framework for community-curated bioinformatics pipelines. Nat Biotechnol. 2020;38(3):276–8.

12. Kent WJ, Sugnet CW, Furey TS, Roskin KM, Pringle TH, Zahler AM, et al. The Human Genome Browser at UCSC. Genome Res. 2002;12(6):996–1006.

13. Love MI, Huber W, Anders S. Moderated estimation of fold change and dispersion for RNA-seq data with DESeq2. Genome Biol. 2014;15(12):550.

14. McLean CY, Bristor D, Hiller M, Clarke SL, Schaar BT, Lowe CB, et al. GREAT improves functional interpretation of cis-regulatory regions. Nat Biotechnol. 2010;28(5):495–501.

15. Bentsen M, Goymann P, Schultheis H, Klee K, Petrova A, Wiegandt R, et al. ATAC-seq footprinting unravels kinetics of transcription factor binding during zygotic genome activation. Nat Commun. 2020;11(1):4267.

16. Fornes O, Castro-Mondragon JA, Khan A, Lee R van der, Zhang X, Richmond PA, et al. JASPAR 2020: update of the open-access database of transcription factor binding profiles. Nucleic Acids Res. 2020;48(D1):D87–92.
